# Efficient enumeration of monocyclic chemical graphs with given path frequencies

**DOI:** 10.1186/1758-2946-6-31

**Published:** 2014-05-30

**Authors:** Masaki Suzuki, Hiroshi Nagamochi, Tatsuya Akutsu

**Affiliations:** 1 Department of Applied Mathematics and Physics, Graduate School of Informatics, Kyoto University Yoshida, 606-8501 Kyoto, Japan; 2 Bioinformatics Center, Institute for Chemical Research, Kyoto University, Gokasho, Uji, 611-0011 Kyoto, Japan

**Keywords:** Chemical graphs, Enumeration, Monocyclic structure, Feature vector

## Abstract

**Background:**

The enumeration of chemical graphs (molecular graphs) satisfying given constraints is one of the fundamental problems in chemoinformatics and bioinformatics because it leads to a variety of useful applications including structure determination and development of novel chemical compounds.

**Results:**

We consider the problem of enumerating chemical graphs with monocyclic structure (a graph structure that contains exactly one cycle) from a given set of feature vectors, where a feature vector represents the frequency of the prescribed paths in a chemical compound to be constructed and the set is specified by a pair of upper and lower feature vectors. To enumerate all tree-like (acyclic) chemical graphs from a given set of feature vectors, Shimizu et al. and Suzuki et al. proposed efficient branch-and-bound algorithms based on a fast tree enumeration algorithm. In this study, we devise a novel method for extending these algorithms to enumeration of chemical graphs with monocyclic structure by designing a fast algorithm for testing uniqueness. The results of computational experiments reveal that the computational efficiency of the new algorithm is as good as those for enumeration of tree-like chemical compounds.

**Conclusions:**

We succeed in expanding the class of chemical graphs that are able to be enumerated efficiently.

## Introduction

The enumeration of chemical structures satisfying given constraints is an important topic in chemoinformatics [1-3]. Applications of the enumeration of chemical structures include structure determination using mass-spectrum and/or NMR-spectrum [4,5], virtual exploration of the chemical universe [6,7], reconstruction of molecular structures from their signatures [8,9], and classification of chemical compounds [10]. The enumeration problem is also important for development of novel chemical compounds because virtual exploration of chemical universe and reconstruction of molecular structures from their signatures are considered to be important elementary technologies. The enumeration of chemical structures has a long history. Cayley [11] considered the enumeration of structural isomers of alkanes in the 19th century. The seminal work of Pólya on counting the number of isomers using group theory is also famous [12].

**Figure 1:**
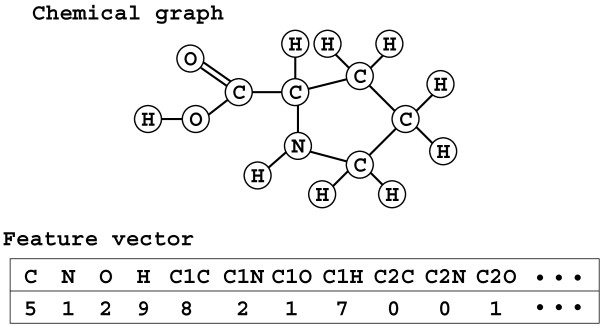
**Example of a chemical structure and its feature vector.** In this example, a feature vector consists of the number of occurrences of each atom type and each bond type (e.g., C2C denote the double bond between two carbon atoms). Note that the entry of C1C is 8 because each bond is counted for both directions. Since there may exist multiple structures with the same feature vector, enumeration of such structures is required.

In this paper, we consider the problem of enumerating chemical structures having monocyclic graph structures satisfying a given constraint, where a *monocyclic graph* is an undirected connected graph containing exactly one cycle (a graph is connected if there exists a path connecting every pair of vertices), and a constraint is given in the form of a set of *feature vectors* (i.e., a set of descriptors). We assume that each feature vector specifies the number of occurrences of each labeled path of length up to a given constant *K*, where a labeled path is an alternating sequence of atom names and bond types (see Figure 1 for an example of a feature vector). We also assume that a set of feature vectors is given by specifying the minimum and maximum numbers of occurrences of each labeled path. We develop an efficient algorithm for this enumeration problem by extending existing algorithms [13,14] for enumerating tree-like chemical structures (i.e., chemical structures without cycles). In this extension, some novel concepts are introduced and rigorous mathematical analysis is performed in orer to guarantee the correctness of the algorithm.

In order to verify the computational efficiency of our proposed algorithm, we perform computational experiments using a set of some chemical compounds from the KEGG LIGAND database [15]. The results suggest that the proposed algorithm enumerates chemical structures having monocyclic graph structures as nearly efficiently as tree-like chemical graphs have been enumerated.

The rest of this paper is organized as follows. First, we review some mathematical definitions and give a formal definition of the enumeration problem for chemical structures with monocyclic graph structures. Next, we review background and related work. Then, we present the algorithm and the results of computational experiments. Finally, we conclude with future work. Mathematical proofs, pseudocodes for the algorithm, and some details on computational experiments are given in Additional file 1.

## Preliminaries and problem formulation

**Figure 2:**
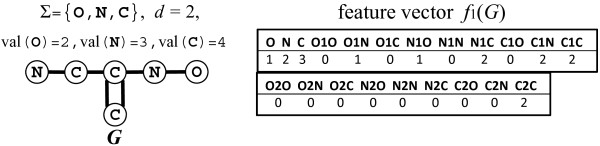
**Example of a *Σ *- *colored * multi-graph *G * and its feature vector*f***_**1**_**( *G *).***G* represents a hydrogen-suppressed chemical graph, where deg(*v*;*G*)<val(*c*(*v*)) holds for some vertex *v*∈*V*(*G*). Since each path is counted for both directions, the entry for C2C is not one, but two.

This section reviews some basic definitions on graphs and formalizes the problem to be addressed in this work. Before providing formal descriptions, we briefly explain the problem definition using an example in Figure 2.

**Figure 3:**
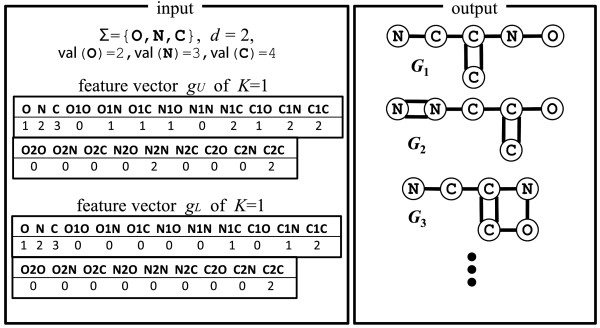
**Example of an input of EULF and part of its output.** The input includes upper and lower feature vectors, and the output includes multi-trees *G*_1_ and *G*_2_ and a 1-augmented tree *G*_3_.

Our basic problem is to enumerate all chemical structures each of which is consistent with a given feature vector and the valence condition. Each coordinate of a feature represents the number of occurrences of vertex- and edge-labeled paths. In order to keep the size of a feature vector moderate, we restrict the length of paths to be no greater than a constant *K*. In the example of Figure 2, we consider paths of lengths 0 and 1, where a path of length 0 corresponds to a single atom and a path of length 1 corresponds to a bond including its endpoint atoms. For example, the columns O, N, and C of feature vector *f*_1_(*G*) mean that each target structure must contain exactly one oxygen, two nitrogen, and three carbon atoms, respectively. The columns N1O, N1C, and C2C mean that each target structure must contain exactly one single bond connecting N and O, two single bonds connecting C and N, and one double bond connecting C and C. It should be noted that one single bond connecting N and O is counted by both O1N and N1O. Then, the chemical structure *G* is consistent with *f*_1_(*G*). However, another chemical structure may be consistent with a given feature vector. For example, the feature vector remains the same even if the double bond (along with the branching carbon atom) is moved into the backbone chain. Therefore, it is desirable to enumerate all chemical structures consistent with a given feature vector and the valence condition (specified by val(…)). On the other hand, there may not exist any consistent chemical structure if *K* is large; thus it may not be appropriate to uniquely specify a feature vector. Therefore, we assume in our target problem that upper and lower bounds of the number of occurrences of each labeled path are given as shown in Figure 3.

**Figure 4:**
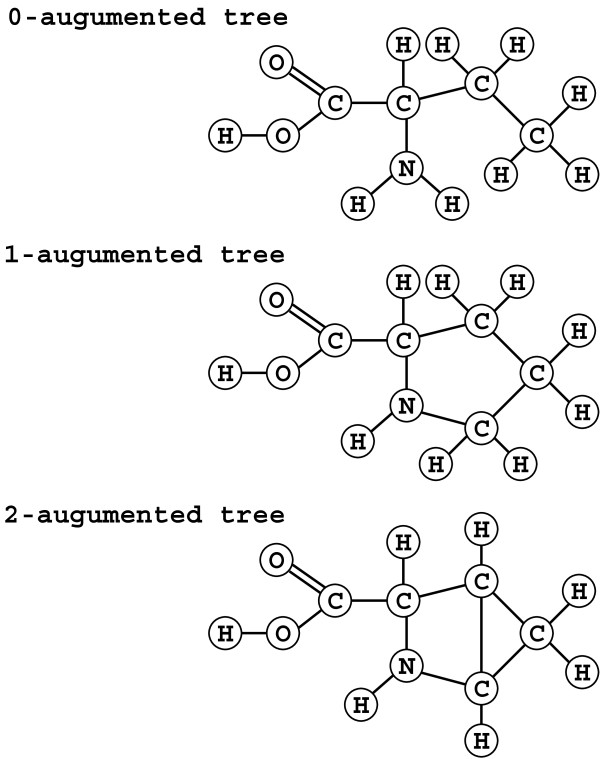
**Examples of *k *-augmented trees.** A *k*-augmented tree is obtained by adding *k* edges to a multi-tree, where a multi-tree is a tree with multiple bonds (precisely, multiple edges between the same pair(s) of vertices).

A multi-graph is a graph that can have multiple edges between the same pair of vertices, where vertices correspond to atoms and multi-edges correspond to double and triple bonds in chemical compounds. We call a connected multi-graph a *k-augmented tree* if the number of adjacent vertex pairs (i.e., vertex pairs connected by edges) minus the number of vertices is *k*−1 (hence a multi-tree is a 0-augmented tree). That is, a *k*-augmented tree is a graph obtained by adding edges to *k* different pairs of nonadjacent vertices in a multi-tree (see Figure 4). The problem considered in this paper is to enumerate all 1-augmented trees satisfying specified upper and lower bound conditions on feature vectors. In the following, we provide the mathematical definition of the problem. We assume that readers have some familiarity with basic concepts in graph theory. For those who are not familiar with graph theory, we suggest referring to an appropriate textbook (e.g., [16]). Readers not interested in mathematical details can skip this part.

A graph is defined to be an ordered pair (*V*,*E*) of a finite set *V* of vertices and a finite set *E* of edges, where an edge is an unordered pair of distinct vertices (thus no self-loop exists), where an edge with two end-vertices *u* and *v* is denoted by *uv*. A graph is called a *multi-graph* when *E* is not necessarily composed of distinct pairs of vertices (thus multiple edges are allowed in a multi-graph and *E* is no longer a set, but a multi-set), and is called a *simple graph* if no multiple edges are allowed. The multiplicity (the number of multiple edges) between two vertices *u* and *v* is denoted by *m*(*u*,*v*). An edge in a multi-graph *G* is called *simple* if its multiplicity in *G* is one. We denote the vertex set and edge set of a graph *G* by *V*(*G*) and *E*(*G*), respectively. For a vertex *v* in a multi-graph *G*, let deg(*v*;*G*) denote the number of edges incident to vertex *v* (i.e., degree of *v*). In this paper, a cycle is a closed path with a length at least three (two edges with the same endvertices are not treated as a cycle), and a connected multi-graph (resp., simple graph) with no cycle is a *multi-tree* (resp., *simple tree*). A *k-augmented tree* is a connected multi-graph such that the number of adjacent vertex pairs (i.e., vertex pairs connected by edges) minus the number of vertices is *k*−1. For two vertices *u* and *v* in a multi-graph *G*, let *G*+*uv* denote the multi-graph obtained by adding a new edge *uv* to *G*; when *uv*∈*E*(*G*), let *G*−*uv* denote the multi-graph obtained by removing *uv* from *G*. Let ${\mathbb{Z}}_{+}$ denote the set of nonnegative integers, and let *Σ* be a set of colors, which correspond to chemical elements such as H, O and C. Let each color *c*∈*Σ* be associated with a *valence*$\text{val}(c)\in {\mathbb{Z}}_{+}$. A multi-graph *G* is said to be *Σ*−*colored* if each vertex *v* has a color *c*(*v*)∈*Σ*. Chemical compounds can be viewed as *Σ*-*colored*, self-loopless connected multi-graphs, where vertices and colors represent atoms and elements, respectively.

Let $d\in {\mathbb{Z}}_{+}$ be a prescribed integer, which corresponds to the maximum multiplicity of chemical graphs, and *Σ*^*k*,*d*^ denote the set of all alternating sequences (*c*_0_,*m*_1_,*c*_1_,…,*m*_*k*_,*c*_*k*_) consisting of colors *c*_0_,*c*_1_,…,*c*_*k*_∈*Σ* and *m*_1_,*m*_2_,…,*m*_*k*_∈{1,2,…,*d*}. We denote the union of *Σ*^0,*d*^, *Σ*^1,*d*^, *Σ*^2,*d*^,…,*Σ*^*k*,*d*^ by *Σ*^≤*k*,*d*^. Let $F_{k}(\Sigma ,d)$ be the set of all mappings *g* from *Σ*^≤*k*,*d*^ to ${\mathbb{Z}}_{+}$, i.e., $F_{k}(\Sigma ,d)=\{g:\Sigma^{\leq k,d}\to {\mathbb{Z}}_{+}\}$.

For a path *P*=(*v*_0_,*m*_1_,*v*_1_,…,*m*_*k*_,*v*_*k*_) such that *V*(*P*)={*v*_0_,*v*_1_,…,*v*_*k*_}, *E*(*P*)={*v*_0_*v*_1_,*v*_1_*v*_2_,…,*v*_*k*−1_*v*_*k*_}, and *m*_*i*_=*m*(*v*_*i*−1_,*v*_*i*_) is the multiplicity of edge *v*_*i*−1_*v*_*i*_, the length of *P* is defined to be *k*=|*V*(*P*)|−1, and the color sequence *c*(*P*) of *P* is defined to be the sequence *c*(*P*)=(*c*(*v*_0_),*m*_1_,*c*(*v*_1_),…,*m*_*k*_,*c*(*v*_*k*_))∈*Σ*^*k*,*d*^.

Given a multi-graph *G* and a sequence *t*∈*Σ*^*k*,*d*^ for some *k*, let *occ*(*t*,*G*) denote the number of paths *P* in *G* such that *c*(*P*)=*t*. For an integer $K\in {\mathbb{Z}}_{+}$, *the feature vectorf*_*K*_(*G*) of level *K* in *G* is defined to be the |*Σ*^≤*K*,*d*^|-dimensional vector *f*_*K*_(*G*) whose value at each entry *t*∈*Σ*^≤*K*,*d*^ is given by *f*_*K*_(*G*)[ *t*]=*occ*(*t*,*G*). In this paper, we treat hydrogen-suppressed chemical graphs with carbon C, nitrogen N or oxygen O, which are represented by *Σ*-colored multi-graphs *G* with color set *Σ*={O, N, C }. Figure 2 illustrates an example of *Σ*-colored multi-graph *G* that represents a hydrogen-suppressed chemical graph and its feature vector *f*_1_(*G*).

Note that in hydrogen-suppressed chemical graph *G*, deg(*v*;*G*)<val(*c*(*v*)) may hold for some vertex *v*∈*V*(*G*). Let us define the *residue degree* res(*v*) of a vertex *v* to be val(*c*(*v*))−deg(*v*;*G*). In a multi-graph *G*, we interpret res(*v*) of a vertex *v* as the number of hydrogen atoms attached to the vertex *v* (in our proposed procedure, we also interpret res(*v*) as the number of new edges/bonds that can be attached to *v* when *G* is being constructed by adding more edges).

For a vector $g\in F_{K}(\Sigma ,d)$ of level *K*≥1, a multi-graph *G* with *f*_*K*_(*G*)=*g* is a multi-graph such that the occurrence of each path *t*=(*c*_0_,*m*_1_,*c*_1_,…,*m*_*p*_,*c*_*p*_) in *G* with length of at most *K* is completely specified by *g*[ *t*], in particular *V*(*G*)={*t*∣*g*[ *t*]≥1,*t*∈*Σ*^0,*d*^=*Σ*} (i.e., *G* has exactly *g*[ *t*] vertices of color *t*), *E*(*G*)={*t*∣*g*[ *t*]≥1,*t*=(*c*,*m*,*c*^′^)∈*Σ*^1,*d*^} (i.e., *G* has exactly *g*[ (*c*,*m*,*c*^′^)] edges of multiplicity *m* that join a vertex of color *c* and a vertex of color *c*^′^).

For two vectors $g_{L},g_{U}\in F_{K}(\Sigma ,d)$ and an integer *k*≥0, let $G_{k}(\;g_{L},g_{U})$ denote the set of all *Σ*-colored *k*-augmented trees *G* such that *g*_*L*_≤*f*_*K*_(*G*)≤*g*_*U*_ (i.e., *f*_*K*_(*G*)=*g*^′^ for some *g* with *g*_*L*_≤*g*^′^≤*g*_*U*_) and deg(*v*;*G*)≤val(*c*(*v*)), *v*∈*V*(*G*).

Our problem is to enumerate all *k*-augmented trees *G* on a given set of atoms each of which is consistent with one of the feature vectors between the lower and upper vectors $g_{U},g_{L}\in F_{K}(\Sigma ,d)$, such that *g*_*L*_≤*g*_*U*_ (where *g*_*L*_[ *t*]=*g*_*U*_[ *t*] for all *t*∈*Σ*^0,*d*^ since the vertex set is fixed for all *G*).

In what follows, we fix a color set *Σ* and an upper bound *d* on multiplicity. We define the problem of enumerating *k*-augmented trees as follows.

Enumerating chemical graphs with given upper and lower path frequency (EULF)

Given a maximum path length $K\in {\mathbb{Z}}_{+}$ and feature vectors $g_{U},g_{L}\in F_{K}(\Sigma ,d)$ such that *g*_*L*_[ *t*]=*g*_*U*_[ *t*] for all *t*∈*Σ*^0,*d*^, enumerate all multi-graphs $G\in G_{k}(\;g_{L},g_{U})$.

Figure 3 illustrates an example of an input of EULF with upper and lower feature vectors *g*_*L*_ and *g*_*U*_ and part of its output, multi-trees $G_{1},G_{2}\in G_{0}(\;g_{L},g_{U})$ and a 1-augmented tree $G_{3}\in G_{1}(\;g_{L},g_{U})$.

For *k*=0, we have developed an efficient algorithm for EULF [13,14]. The purpose of this work is to describe an algorithm for EULF with *k*=1. We assume that the maximum valence is 4 and mainly enumerate a 1-augmented tree such that the cycle contains an edge of multiplicity one (a single bond), since otherwise a 1-augmented tree is a single cycle consisting of edges of multiplicity two, which can be separately handled as a special rare case.

## Background

As mentioned in Introduction, enumeration of chemical structures has a long history and many studies have been done. In the field of machine learning, a similar problem, which is called the *preimage problem*, has been studied [17,18]. In this problem, a desired object is computed as a feature vector in a feature space, and then the feature vector is mapped back to the input space, where this mapped back object is called a preimage. The definition of the feature vectors based on the frequency of labeled paths [19,20] or small fragments [10,21] has been widely used. Akutsu and Fukagawa [22] formulated the graph preimage problem as the problem of inferring graphs from the frequency of paths of labeled vertices and proved that the problem is computationally intractable (NP-hard) even for planar graphs with bounded degrees [22]. Nagamochi [23] proved that a graph determined by the frequency of paths with length one can be found in polynomial time if any exists.

The preimage problem has also been studied in the field of chemoinformatics as a part of inverse QSAR/QSPR (quantitative structure-activity relationship/quantitative structure-property relationship) studies. Indeed, the problem is essentially the same as reconstruction and/or enumeration of molecules from their descriptors in inverse QSAR/QSPR [8,9,24,25], where the descriptors correspond to feature vectors in the preimage problem. Wong and Burkowski developed a practical preimage based method and demonstrated that it actually generated the structure of a new drug candidate [26]. For enumeration of molecules from descriptors, useful tools such as MOLGEN have been developed [27]. However, they are not very efficient if large structures are to be enumerated because many of them treat general graph structures (under the valence constraint).

It might be possible to develop significantly faster algorithms for the preimage problem if we restrict the class of target chemical structures and employ recent techniques for enumeration of graph structures. Fujiwara *et al.*[28] studied enumeration of tree-like chemical graphs that satisfy a given feature vector which specifies frequency of paths of up to a prescribed length *K* in a chemical compound to be constructed. They proposed a branch-and-bound algorithm that consists of a branching procedure based on the tree enumeration algorithm by Nakano and Uno [29,30] and bounding operations designed by properties on path frequency and atom-atom bonds. They showed by means of computational experiments on enumeration of alkane isomers that their algorithm works at least as efficiently as the fastest algorithm while using much less memory space.

To reduce the size of the search space, Ishida *et al*. [31] have introduced a new bounding operation, called the *detachment-cut*, based on the result of Nagamochi [23]. In this problem formulation, it is required that the path frequency of a chemical structure is exactly the same as the specified one. However, there does not exist such a structure in many cases because a mapping between chemical structures and feature vectors is not surjective and thus there are many vectors in a feature space that do not have preimages. To seek solutions effectively in a relaxed constraint, Shimizu *et al*. [13] recently introduced a problem of enumerating tree-like hydrogen-suppressed chemical graphs that satisfy one of a given set of feature vectors which is specified by a pair of upper and lower feature vectors. They proposed a branch-and-bound algorithm for the problem, called 1-Phase algorithm, and afterward Suzuki *et al*. [14] proposed a more efficient and effective algorithm, called 2-Phase algorithm. Implementations of these algorithms [13,14] for enumerating tree-like hydrogen-suppressed/hydrogen-retained chemical graphs with given upper and lower bounds on path frequencies are available on a web server (*http://sunflower.kuicr.kyoto-u.ac.jp/tools/enumol2/*).

As shown by Nakano and Uno [29,30], the class of trees admits a nice scheme for computer representation of their structures (called “left-heavy trees”) which enables us to generate trees significantly faster (in constant time per tree) without executing any explicit test on the uniqueness of structure representations of temporarily generated labeled graphs. Development of algorithms for enumerating chemical graphs with a “non-tree structure” is thereby a challenging task if we still wish to attain high computational efficiency as we have achieved for enumeration of tree-like chemical graphs, because no such effective representation scheme is known for general graphs. It should be noted that although polynomial-time algorithms have been developed for equivalence test and unique representation form problems for bounded degree graphs [32,33] and chemical compounds [34], they are not directly applicable to efficient enumeration of chemical graphs.

In the NCI database (*http://cactus.nci.nih.gov/ncidb2.2/*), the ratio of the number of chemical compounds with *k*-augmented tree structures to that of all registered chemical compounds is approximately 9*%*, 22*%*, 28*%*, 20*%*, and 11*%* for *k*=0,1,2,3, and 4, respectively. This implies that we have been able to treat only 9*%* of all of chemical compounds with high computational efficiency. As the first step toward efficient enumeration of non-tree chemical graphs, we consider the problem of hydrogen-suppressed chemical graphs with 1-augmented tree (monocyclic) structure. If we can solve this problem, we can treat 31*%* (=9*%*+22*%*) of chemical compounds. Although no effective representation scheme is known even to 1-augmented trees, we can create a tree by removing one edge in the unique cycle in a 1-augmented tree (two multiple edges with the same endvertices is not called a cycle in this paper). Additionally, 2-Phase algorithm [14], which enumerates tree-like hydrogen-suppressed chemical graphs, can be used without any major modification to enumerate such trees *T*=*G*−*e* with one edge deficit from 1-augmented trees *G* to be constructed. Thus the main task is to efficiently test the uniqueness of generated labeled 1-augmented trees. To design such a procedure, we use a well-reflected definition of a parent 0-augmented tree *T*=*G*−*e* of a 1-augmented tree *G*. As a result, we can combine the new procedure with 2-Phase algorithm to obtain an algorithm for enumerating hydrogen-suppressed chemical graphs with 1-augmented tree structure from upper and lower bounds on feature vectors.

## Method

Our proposed algorithm is based on existing algorithms to enumerate colored trees [29,30] and colored multi-trees [13,14,28,31]. The basic strategy of our algorithm is to generate a multi-tree first and then extend it to a 1-augmented tree by adding an edge. In enumeration algorithms, it is important not to miss any possible structures and not to duplicate identical structures. In order to efficiently cope with these conditions, the concept of the family tree has been widely employed in various enumeration algorithms. To define a family tree for graphs, we need to define a parent-child relationship between graph structures so that a parent structure is uniquely determined from a child structure, where each child structure is obtained by adding a vertex or an edge to its parent structure. Because extension of a multi-tree to a 1-augmented tree is the core part of our proposed algorithm, we need to provide a proper definition of the parent-child relationship between a multi-tree and a 1-augmented tree. As will be shown later, there may exist multiple possible ways of having a parent structure. How to define the unique parent of a given 1-augmented tree is one of the novel points of our proposed algorithm. Another important issue on generating 1-augmented trees is not to generate identical 1-augmented trees from the same multi-tree. As will be shown later, there is a case in which additions of different edges result in identical structures. How to efficiently prevent this kind of duplicate generation of identical structures is the other novel point of our proposed algorithm. In the following, we give a detailed description of the algorithm including these novel points. Again, readers not interested in mathematical details can skip this part.

### Overview of a new algorithm for 1-Augmented trees

Let $G_{0}^{\prime }$ be the set of 0-augmented trees (multi-trees) *T*=*G*−*e* obtained from each 1-augmented tree $G\in G_{1}(\;g_{L},g_{U})$ by removing a simple edge *e* in the unique cycle of *G*.

Then we have $G_{0}^{\prime }\subseteq G_{0}(\;g_{L}^{\prime },g_{U})$ for a modified lower vector $g_{L}^{\prime }$ in a vector set $g_{L}^{\prime }$. We construct such a vector set $g_{L}^{\prime }$ from *g*_*L*_ as follows: For each *t*∈*Σ*^*k*,*d*^ with *k*≥2, let $g_{L}^{\prime }[\;t]=0$; and for each *t*∈*Σ*^1,*d*^, let

$$\;g_{L}^{\prime }[\;t]=\left\{\begin{array}{ll} \max\{g_{L}[\;t]-1,0\} & \;\text{if}t\text{is symmetric (i.e., it is identical with its reversal)}\\ \max\{g_{L}[\;t]-2,0\} & \;\text{otherwise}. \end{array}\right)$$

Thus our first task is to generate all multi-trees $T\in G_{0}(\;g_{L}^{\prime },g_{U})$ by using fast conventional algorithms such as 2-Phase algorithm.

Our next task is to generate 1-augmented trees *G* from each multi-tree $T\in G_{0}(\;g_{L}^{\prime },g_{U})$ such that no 1-augmented tree in $G\in G_{1}(\;g_{L},g_{U})$ will be duplicated during the entire enumeration over all $T\in G_{0}(\;g_{L}^{\prime },g_{U})$. To attain this objective without storing all generated 1-augmented trees for a comparison with a newly generated 1-augmented tree, we define a mapping $\pi :G_{1}(\;g_{L},g_{U})\to G_{0}(\;g_{L}^{\prime },g_{U})$; the multi-tree *T*=*π*(*G*) for a 1-augmented tree *G* is called the *parent* of *G*. For a multi-tree *T*, a 1-augmented tree *G* with *π*(*G*)=*T* is called a *child* of *T* (possibly *T* has more than one child), and is called a *feasible child* of *T* if $G\in G_{1}(\;g_{L},g_{U})$. Note that any of definition of such a mapping will suffice as long as *π*(*G*) is determined only by the information of an “unlabeled graph” *G* (i.e., topological structure) except for a possible difference in computational efficiency to avoid duplication of solutions.

In the following, we show the 2-Phase algorithm, present details of our definition of parents *π* and design an efficient procedure for generating all children *G* from a given multi-tree $T\in G_{0}(\;g_{L}^{\prime },g_{U})$.

### Summary of 2-Phase algorithm for 0-Augmented tree

**Figure 5:**
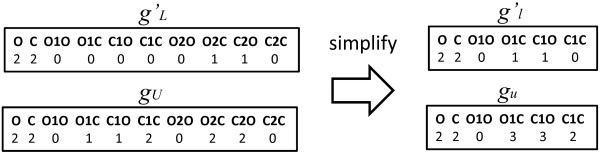
**Illustration of modified feature vectors.** The frequency of O1C and C1O increases after the simplification.

In this section, we summarize 2-Phase Algorithm [14] for generating all multi-trees in $G_{0}(\;g_{L}^{\prime },g_{U})$. In the first phase, we simplify input feature vectors by adding the frequencies of the paths that include multiple edges to the corresponding paths which consist of only simple edges and then enumerate simple trees for the simplified upper and lower feature vectors. Figure 5 illustrates feature vectors *g*_*U*_ and $g_{L}^{\prime }$ and simplified feature vectors *g*_*u*_ and $g_{L}^{\prime }$.

In the second phase, we assign multiplicities of edges for each of the simple trees to satisfy the feature vector constraint and the valence constraint. The inputs and outputs of the first phase and second phase in 2-Phase Algorithm are described as follows:

From the given multi-tree $T\in G_{0}(\;g_{L}^{\prime },g_{U})$, our efficient procedure generates all 1-augmented trees in $G_{1}(\;g_{L},g_{U})$.

### Parent-child relationship

In this section, to avoid duplication of a 1-augmented tree during the entire enumeration over all $T\in G_{0}(\;g_{L}^{\prime },g_{U})$, we introduce a parent-child relationship between a 0-augmented tree and a 1-augmented tree.

#### Signature of rooted multi-trees

To define the parent *π*(*G*) of a 1-augmented tree *G* using only topological structure, we first introduce the concepts of “canonical form” and “signature” for a class of multi-graphs.

We fix the total order of colors in *Σ* arbitrarily, e.g., O <N <C, and regard each color *c*∈*Σ* as a small integer in ${\mathbb{Z}}_{+}$. We define the *lexicographical order* among sequences with elements in $\Sigma \cup {\mathbb{Z}}_{+}$ as follows. A sequence *A*=(*a*_1_,*a*_2_,…,*a*_*p*_) is lexicographically smaller than a sequence *B*=(*b*_1_,*b*_2_,…,*b*_*q*_) (denoted by *A*<*B*) if and only if there is an index *k* such that (i) *a*_*i*_=*b*_*i*_(1≤*i*≤*k*); and (ii) *a*_*k*+1_<*b*_*k*+1_(*k*+1≤ min{*p*,*q*}) or *k*=*p*<*q*; otherwise *A*=*B*, i.e., *p*=*q* and *a*_*i*_=*b*_*i*_(1≤*i*≤*p*), or *B*<*A*. Let *A*≤*B* denote *A*<*B* or *A*=*B*.

A multi-graph is called *labeled* if each vertex has a unique name or an index such as *v*_0_,*v*_1_,…,*v*_*n*−1_, and we usually record a multi-graph as labeled in our computer. Hence, testing isomorphism of two multi-graphs is to find labels for these “unlabeled graphs” such that the two labeled graphs completely match each other including the adjacency between every two vertices. For a class  of multi-graphs, if we have a way of choosing a label for each multi-graph G∈G that is unique up to automorphisms of *G*, then we can test the isomorphism of two graphs directly with their labels. Such a labeling for *G* is called the *canonical form* of *G*. Once such a canonical form is obtained, we can easily encode each multi-graph G∈G into a code *σ*(*G*) (which is an integer or a sequence of integers/colors), called the *signature* of *G*, such that two multi-graphs $G,G^{\prime }\in G$ are isomorphic if and only if *σ*(*G*)=*σ*(*G*^′^). Without loss of generality we assume a total order over {σ(G)∣G∈G} by introducing, if necessary, a total order over all colors and a lexicographical (total) order over all sequences of integers and colors.

A *rooted* graph is a multi-graph in which a vertex is designated as the root, and two rooted graphs are isomorphic if there is an isomorphism that maps their roots onto each other.

Any tree *T* has either a vertex or a pair of adjacent vertices removal of which leaves no component with at least |*V*(*T*)|/2 vertices [35], where the former is called the centroid and the latter is called the bicentroid.

In a rooted multi-tree *T*, the parent vertex of a non-root vertex *v* is denoted by *p*(*v*) and the *depth* of a vertex *v* is denoted by depth(*v*), where the depth of a vertex is its distance to the root. For a vertex *v* in *T*, let *T*_*v*_ denote the subtree induced from *T* by all descendants of *v* including *v*. For an edge *e*=*uv* in *T* (where *u*=*p*(*v*)), let *T*_*e*_ (*T*_*u* *v*_) denote the subtree of *T* that consists of *T*_*v*_ and *u*=*p*(*v*) joined by edge *e*=*uv*.

**Figure 6:**
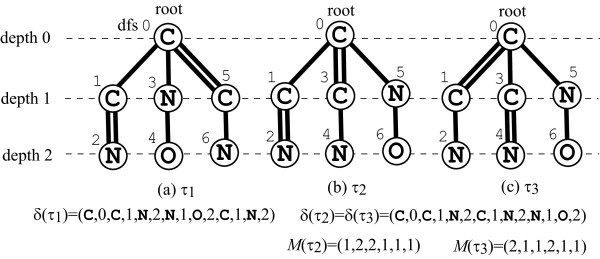
**Illustration of a rooted tree and left-heavy trees.(a)** An ordered tree *τ*_1_ rooted at its centroid; **(b)** a left-heavy tree *τ*_2_; and **(c)** the canonical form *τ*_3_.

For the class of rooted multi-trees, a canonical form of a rooted multi-tree *T* is given by an “ordered tree” *τ* of it (i.e., determination of a total order among children of each vertex). Let dfs(*τ*) denote the total order of vertices in *τ* visited by the depth-first-search order according to the order for children in *τ*. For example, Figure 6 illustrates three ordered trees *τ*_1_, *τ*_2_ and *τ*_3_, which are obtained from the same multi-tree *T* rooted at the centroid, where the number beside each vertex *v* indicates dfs(*v*).

We let *δ*(*τ*) denote the alternating sequence (*c*_0_,*d*_0_,*c*_1_,*d*_1_,…,*c*_*n*−1_,*d*_*n*−1_) such that *c*_*i*_ and *d*_*i*_ denote the color and depth, respectively, of the *i*-th vertex *v*_*i*_ in dfs(*τ*), and *M*(*τ*) denote the sequence (*m*_1_,*m*_2_,…,*m*_*n*−1_) of the multiplicity *m*_*i*_=*m*(*v*_*i*_,*p*(*v*_*i*_)) of the edge joining the *i*-th vertex and its parent *p*(*v*_*i*_) in *T*. For a vertex *v*, let dfs(*v*) denote the labeling number of *v* in dfs(*τ*). For example, Figure 6 illustrates *δ*(*τ*_*i*_) of ordered trees *τ*_*i*_, *i*=1,2,3 and *M*(*τ*_2_) and *M*(*τ*_3_).

A *left-heavy* tree of a rooted multi-tree *T* is an ordered tree *τ* that has the maximum code *δ*(*τ*) among all ordered trees of *T* (hence a left-heavy tree *τ* is a canonical form and *δ*(*τ*) is a signature of it when we ignore the multiplicity of rooted multi-trees). We define the canonical form of a rooted multi-tree *T* to be the left-heavy tree *τ* that has the maximum code *M*(*τ*) among all left-heavy trees of *T*, and let *σ*(*T*) denote a signature of *T* (a code of the canonical form *τ* such as (*δ*(*τ*),*M*(*τ*))). For example, in Figure 6, *τ*_2_ and *τ*_3_ are left-heavy trees of *T*, since they have lexicographically maximum sequences *δ*(*τ*_2_)=*δ*(*τ*_3_) among all ordered trees *τ* of the rooted multi-tree *T*, and *τ*_3_ is the canonical form of *T* and (*δ*(*τ*_3_)=(C,0,C,1,N,2,C,1,N,2,N,1,O,2),*M*(*τ*_3_)=(2,1,1,2,1,1)) is the signature of *T* since it is a left-heavy tree with the lexicographically maximum *M*(*τ*_3_) among all left-heavy trees *τ* of *T*.

Using the canonical form for rooted multi-trees, we can define a canonical form for “unrooted” multi-trees *T* by regarding them as trees rooted at the centroid or bicentroid.

#### Defining parents *π*

We are now ready to define parents *π* for 1-augmented trees (note that there is no root for any 1-augmented tree). Let *G* be a 1-augmented tree with a unique cycle *C* of length *p* which by our assumption contains at least one simple edge. Then there are *p* possible choices *G*−*e*_*i*_, *i*=1,2,…,*p*, for the parent of *G*. We introduce a rule to choose one of them based only on the topological information on *G* and *C*. For each vertex *v* in *C*, let *N*(*v*) denote the set of vertices in *V*−*V*(*C*) adjacent to *v*. Removing an edge *vw* with *v*∈*V*(*C*) and *w*∈*N*(*v*) leaves a multi-tree containing *w*, which we denote by *T*_*w*_. For each vertex *v* in *C*, we encode all multi-trees *T*_*w*_, *w*∈*N*(*v*) into a signature *σ*^∗^(*v*) using the signature *σ* for rooted multi-trees; we set

$$\sigma^{*}(v)=(c(v),\sigma (T_{w_{1}}),\sigma (T_{w_{2}}),\ldots ,\sigma (T_{w_{h}})),$$

such that $\sigma (T_{w_{1}})\geq \sigma (T_{w_{2}})\geq \cdots \geq \sigma (T_{w_{h}})$ holds for *N*(*v*)={*w*_1_,*w*_2_,…,*w*_*h*_}. Note that two vertices *v* and *v*^′^ in *C* have the same color and an identical set of subtrees in *N*(*v*) and *N*(*v*^′^) if and only if *σ*^∗^(*v*)=*σ*^∗^(*v*^′^). For each simple edge *e*=*uv* in *C*, we define a code *c*^∗^(*e*) as follows. We encode the unique path *u*_1_ (=*u*),*u*_2_,…,*u*_*h*_ (=*v*) from *u* to *v* along *C* into *σ*^∗^(*u*,*v*)=(*σ*^∗^(*u*_1_),*m*_1_,*σ*^∗^(*u*_2_),*m*_2_,…,*m*_*h*−1_,*σ*^∗^(*u*_*h*_)), where *m*_*i*_=*m*(*u*_*i*_,*u*_*i*+1_). Symmetrically, we define *σ*^∗^(*v*,*u*) = (*σ*^∗^(*u*_*h*_),*m*_*h*−1_,*σ*^∗^(*u*_*h*−1_),*m*_*h*−2_,…,*m*_1_,*σ*^∗^(*u*_1_)). The code *c*^∗^(*e*) is defined to be lexicographically the maximum one between two sequences *σ*^∗^(*u*,*v*) and *σ*^∗^(*v*,*u*). Furthermore, let *E*^∗^(*C*) be the set of simple edges *e*^∗^ in *C* such that *c*^∗^(*e*^∗^) is lexicographically maximum among *c*^∗^(*e*) for all simple edges *e* in *C*.

We call an edge *vw* with *v*∈*V*(*C*) and *w*∈*N*(*v*) a *heavy edge* if *T*_*w*_ has at least |*V*(*G*)|/2 vertices. We distinguish two cases to define parent *π*. 

**Figure 7:**
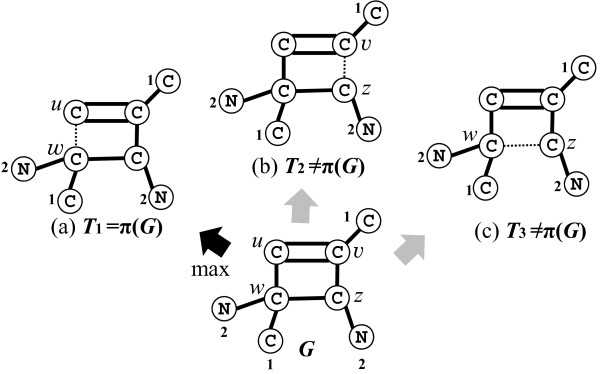
**Illustration of defining the parent*π*(*G*) of a 1-augmented tree*G*.** Three multi-trees *T*_1_=*G*−*uw*, *T*_2_=*G*−*vz* and *T*_3_=*G*−*wz* are obtained from *G* by removing a simple edge in the cycle. Each number on the left side of each vertex *v* in *G* indicates its signature *σ*^∗^(*v*) of {*T*_*w*_∣*w*∈*N*(*v*)}. The code *σ*^∗^ for each pair of adjacent vertices in the cycle of *G* is given by *σ*^∗^(*w*,*u*)=((*C*,2,1),1,(*C*,2),1,(*C*,1),2,(*C*)), *σ*^∗^(*u*,*w*)=((*C*),2,(*C*,1),1,(*C*,2),1,(*C*,2,1)), *σ*^∗^(*v*,*z*)=((*C*,1),2,(*C*),1,(*C*,2,1),1,(*C*,2)), *σ*^∗^(*z*,*v*)=((*C*,2),1,(*C*,2,1),1,(*C*),2,(*C*,1)), *σ*^∗^(*w*,*z*)=((*C*,2,1),1,(*C*),2,(*C*,1),1,(*C*,2)), and *σ*^∗^(*z*,*w*)=((*C*,2),1,(*C*,1),2,(*C*),1,(*C*,2,1)). Then *π*(*G*) is defined to be *T*_1_=*G*−*uw* because *σ*^∗^(*w*,*u*) is maximum over all of these six codes.

● There is no heavy edge around *C*: For an arbitrary edge *e*∈*E*^∗^(*C*), we define *π*(*G*) to be *G*−*e* (note that when |*E*^∗^(*C*)|≥2, *G* is symmetric around *C* and *G*−*e* and *G*−*e*^′^ will be isomorphic for any two edges *e*,*e*^′^∈*E*^∗^(*C*)). Figure 7 illustrates how the parent *π*(*G*) of a 1-augmented tree *G* in Case 1 is determined on these signatures *σ*(*u*,*v*) and *σ*(*v*,*u*) of simple edges *uv* in the cycle *C*.

● There is a heavy edge *v*^∗^*w*^∗^: Note that no other edge can be a heavy edge. Let *e*_1_ and *e*_2_ be the two edges in *C* that are adjacent to *v*^∗^, where at least one of them is a simple edge since deg(*v*)≤4. If exactly one of them, e.g., *e*_1_ is a simple edge, then we define *π*(*G*) to be *G*−*e*_1_. When *e*_1_ and *e*_2_ are simple edges, we choose one of them as follows. We first ignore all trees *T*_*w*_ with *w*∈*N*(*v*^∗^), which are symmetric at the vertex *v*^∗^ commonly shared by *e*_1_ and *e*_2_ and hence useless to construct a signature for distinguishing *e*_1_ and *e*_2_. Without using *T*_*w*_ with *w*∈*N*(*v*^∗^), we construct the code *c*^∗^(*e*_1_) and *c*^∗^(*e*_2_). Finally we choose any edge *e*_*i*_ such that *c*^∗^(*e*_*i*_) is lexicographically maximum between *c*^∗^(*e*_1_) and *c*^∗^(*e*_2_), and define *π*(*G*) to be *G*−*e*_*i*_.

#### Generating children

Recall that our algorithm for enumerating 1-augmented trees consists of two major stages: the first stage enumerates all multi-trees $T\in G_{0}(\;g_{L}^{\prime },g_{U})$ by 2-Phase algorithm, and the second stage generates all feasible children *G* for each $T\in G_{0}(\;g_{L}^{\prime },g_{U})$, i.e., 1-augmented trees $G\in G_{1}(\;g_{L},g_{U})$ with *π*(*G*)=*T*. This section describes a procedure for generating all children *G*=*T*+*e* of a given multi-tree *T* by adding a new edge *e*.

For simplicity, we consider the case where a given multi-tree *T* has the centroid (the case where it is rooted at the bicentroid can be treated with a minor technical modification). In the following, we assume that a given multi-tree *T* is represented as its canonical form (a left-heavy tree) *τ* rooted at its centroid, and that its sequences *δ*(*τ*)=(*c*_1_,*d*_1_,…,*c*_*n*_,*d*_*n*_) and *M*(*τ*)=(*m*_2_,*m*_3_,…,*m*_*n*_) over the labeling dfs(*τ*) have been already computed after the first stage (2-Phase algorithm can deliver not only solutions *T* but also *τ* and these sequences together).

It should be noted that the canonical form of left-heavy trees enjoys the following recursive structure. For any vertex *v* in *T*, the subtree *T*_*v*_ of *T* rooted at *v* induces an ordered tree *τ*_*v*_ from the left-heavy tree *τ* and *τ*_*v*_ is again the canonical form of *T*_*v*_, since dfs(*τ*_*v*_) is a subsequence of dfs(*τ*) with consecutive vertices and its ordered pair (*δ*(*τ*_*v*_),*M*(*τ*_*v*_)) is also lexicographically maximized over all ordered trees of *T*_*v*_.

#### Testing generated 1-Augmented trees

Given the left-heavy tree *τ* of a multi-tree *T*, we add a new edge *xy* for two nonadjacent vertices *x*,*y*∈*V*(*T*)(dfs(*x*)<dfs(*y*)) to obtain *G*=*T*+*xy*. Let *C* denote the cycle created in *G*. We check the following condition to test whether *T* is the parent of *G* or not. 

Case I. *C* contains the root (centroid) of *T*: 

● *σ*^∗^(*xy*) is lexicographically maximum among *σ*^∗^(*e*) for all simple edges *e* in *C*.

Case II. Otherwise: 

● *x* is the ancestor of *y*;

● *σ*^∗^(*xy*)≥*σ*^∗^(*e*) if the edge *e* incident to *x* in *C* is simple.

Then, we have the following lemma, where the proof is given in S1.1 (of the Additional file 1).

##### **Lemma1**.

For a multi-tree *T* and two nonadjacent vertices *x*,*y*∈*V*(*T*), testing whether *T*=*π*(*T*+*xy*) can be done by checking the above condition in *O*(|*V*(*C*)|^2^) time.

### Avoiding duplication of children

**Figure 8:**
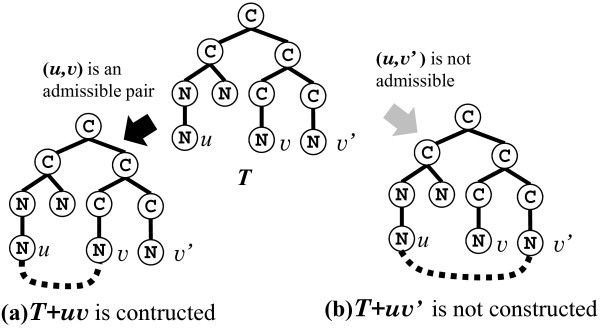
**Illustration of admissible and non-admissible pairs.** A cycle will be created by adding each edge shown by a black dotted line. Two 1-augmented trees *T*+*uv* and *T*+*uv*^′^ are 1-augmented trees obtained from the multi-tree *T* by adding a simple edge such that *T*+*uv* and *T*+*uv*^′^ are isomorphic to each other. **(a)** The vertex pair (*u*,*v*) is an admissible pair, and **(b)** the other is not an admissible pair (at least one admissible pair always exists in every 1-augmented tree). Therefore, *T*+*uv*^′^ is not created (all 1-augmented trees are discarded except for the 1-augmented tree created for admissible pair).

In previous sections, we have showed that all children (i.e., 1-augmented trees) of a given multi-tree *T* can be generated by the definition of the parent-child relationship between multi-tree and 1-augmented tree. However, if *T* has two isomorphic subtrees *T*_*u*_ and *T*_*v*_ then we would have two children *T*+*xy* and *T*+*x*^′^*y*^′^, which are isomorphic to each other. To avoid such duplication, we test if *T*+*xy* is isomorphic to *T*+*x*^′^*y*^′^ for some other vertices *x*^′^ and *y*^′^ when we add an edge *xy* to *T*. In fact, we do not try to find other such pair *x*^′^ and *y*^′^ explicitly. Instead we introduce a rule that we do not generate *T*+*xy* by any edge *xy* that has such an “isomorphic” vertex pair *x*^′^ and *y*^′^ on the left hand side of *x* and *y* in *T*. To detect this situation efficiently, we first compute data on each vertex *v* in *T* that indicates whether the left hand side of *v* contains another vertex *u* such that its subtree *T*_*p*(*u*)*u*_ is isomorphic to *T*_*p*(*v*)*v*_. Using such data, we show that given a vertex pair *x* and *y* whether there is an isomorphic pair *x*^′^ and *y*^′^ in the left hand side can be checked in constant time. In other words, we show an *O*(*n*^2^) time algorithm extracting all the leftmost side vertex pairs from *T*.Among all isomorphic vertex pairs, we call the leftmost one an “admissible pair”, where “isomorphic” means here that the connection of vertices in each pair results in an isomorphic tree. Figure 8 shows an example of a case in which additions of different edges result in identical structures and the admissible pair.

In this section, we show the validity that we only need to add an edge between each admissible pair to avoid duplication and omission of 1-augmented trees generated from one multi-tree *T*. Finally, for a multi-tree, we provide an efficient algorithm extracting all vertex pairs to generate all children of the multi-tree.

#### Admissible pairs

We write *T*+*uv*∼*T*+*u*^′^*v*^′^ if and only if *T*+*uv* and *T*+*u*^′^*v*^′^ are isomorphic. For a tree *T*, let *c*_*T*_ denote its centroid, which is either a vertex (unicentroid) or an edge (bicentroid). Let *T* be a left-heavy tree rooted at its centroid *c*_*T*_. When *c*_*T*_ is a bicentroid *rr*^′^, *r* and *r*^′^ will be the vertices that have no parent in the parent-child relationship in *T*. We shall now introduce “rooted-isomorphism” among 1-augmented trees obtained from *T* by adding a new edge. We regard a 1-augmented tree *G*=*T*+*uv* obtained by adding new edge *uv* between two nonadjacent vertices *u*,*v*∈*V*(*T*) as a graph rooted at *c*_*T*_. When *c*_*T*_ is a vertex *r*, we say that two 1-augmented trees *G*=*T*+*uv* and *G*^′^=*T*+*u*^′^*v*^′^ are *rooted-isomorphic* if they admit an isomorphism *ψ* such that *c*_*T*_ in *G*=*T*+*uv* corresponds to *c*_*T*_ in *G*^′^=*T*+*u*^′^*v*^′^ (i.e., *ψ*(*r*)=*r* when *c*_*T*_ is a vertex *r*, and {*ψ*(*r*),*ψ*(*r*^′^)}={*r*,*r*^′^} when *c*_*T*_ is an edge *rr*^′^). We write $T+\text{uv}\underset{r}{\approx }T+u^{\prime }v^{\prime }$ if and only if *T*+*uv* and *T*+*u*^′^*v*^′^ are rooted-isomorphic with root *r*. Then, the following theorem holds, where the proof is given in Additional file 1: S1.2.

##### **Theorem2**.

Let *T* be a left-heavy tree rooted at its centroid *c*_*T*_ and {*u*,*v*},{*u*^′^,*v*^′^}⊆*V*(*T*) be two pairs of nonadjacent vertices. If *T*+*uv*∼*T*+*u*^′^*v*^′^ then $T+\text{uv}\underset{r}{\approx }T+u^{\prime }v^{\prime }$.

Theorem 2 tells us that two 1-augmented trees *G*=*T*+*uv* and *G*^′^=*T*+*u*^′^*v*^′^ are isomorphic if and only if they are rooted-isomorphic (i.e., *c*_*T*_ in *G* corresponds to *c*_*T*_ in *G*^′^ in the isomorphism *ψ*, where possibly *ψ*(*r*)=*r*^′^ and *ψ*(*r*^′^)=*r* when *c*_*T*_=*rr*^′^).

Now we consider how to generate a set $G_{T}$ of 1-augmented trees *T*+*uv* such that the 1-augmented tree *T*+*uv* for any pair of nonadjacent vertices *u*,*v*∈*V*(*T*) is isomorphic to exactly one 1-augmented tree *G* in the set $G_{T}$. By Theorem 2, we only need to check the rooted-isomorphism among 1-augmented trees *T*+*uv* for all pairs of nonadjacent vertices *u*,*v*∈*V*(*T*). Based on this, we can modify a given tree *T* with bicentroid *c*_*T*_=*rr*^′^ into a tree *T*^′^ with unicentroid *r*^∗^ by inserting a new vertex on the edge *rr*^′^. Since this does not change the rooted-isomorphism among 1-augmented trees *T*^′^+*uv* or the left-heaviness of *T*, we assume in the following that a given tree *T* has a unicentroid *c*_*T*_=*r*.

Let *T* be a left-heavy tree. We shall introduce some terminology. Let *x* be a non-root vertex *x* in *T*. Denote by left(*x*) the immediate left sibling of a non-root vertex *x* (if any). We define data copy as follows.

$$\;\text{copy}(x)=\left\{\begin{array}{ll} 1 & \text{if left}(x)\text{exists and}T_{y}\underset{r}{\approx }T_{x}\text{holds (i.e.,}x\text{is a copy of}y\text{) for}y=\text{left(}x\text{),}\\ 0 & \text{otherwise.} \end{array}\right)$$

Let *u* and *v* be two vertices in *T*. We denote by *P*(*u*,*v*) the unique path in *T* that connects *u* and *v*, where *P*(*u*,*v*)=*P*(*v*,*u*). Let lca(*u*,*v*) denote the least common ancestor of *u* and *v*, i.e., the highest vertex in *P*(*u*,*v*) (where we define lca(*u*,*v*) to be the edge *c*_*T*_=*rr*^′^ when *T* is rooted at the bicentroid *c*_*T*_=*rr*^′^, and *u*∈*V*(*T*_*r*_) and $v\in V(T_{r^{\prime }})$). When dfs(*u*)<dfs(*v*), we define the *greatest uncommon ancestor* gua of *u* and *v* as follows: 

● Let gua(*u*,*v*) denote the child of lca(*u*,*v*) that is closest to *u* in *T*, where gua(*u*,*v*) is an ancestor of *u* (including *u* itself) if lca(*u*,*v*)≠*u*;

● Let gua(*v*,*u*) denote the child of lca(*u*,*v*) that is an ancestor of *v* (including *v* itself), where gua(*u*,*v*)=gua(*v*,*u*) if lca(*u*,*v*)=*u*.

**Figure 9:**
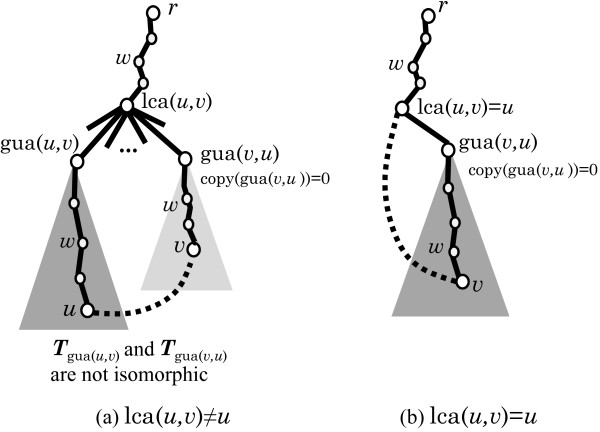
**Illustration of conditions (1) and (2) for admissible pairs.** The black dotted line joins two vertices *u* and *v*, which will be the edge to create a cycle in the 1-augmented tree *T*+*uv*. **(a)** and **(b)** illustrate the case of lca(*u*,*v*)≠*u* (where two rooted subtrees *T*(gua(*u*,*v*)) and *T*(gua(*v*,*u*)) are not isomorphic to each other by copy(gua(*v*,*u*))=0) and the case of lca(*u*,*v*)=*u*, respectively (note that lca(*u*,*v*)≠*v* by dfs(*u*)<dfs(*v*)). Conditions (1) and (2) exclude any ancestor *w* of *u* or *v* such that copy(*w*)=1 (otherwise we can prove that there is a lexicographically smaller pair (*u*^′^,*v*^′^) such that *T*+*u*^′^*v*^′^ is isomorphic to *T*+*uv*).

**Figure 10:**
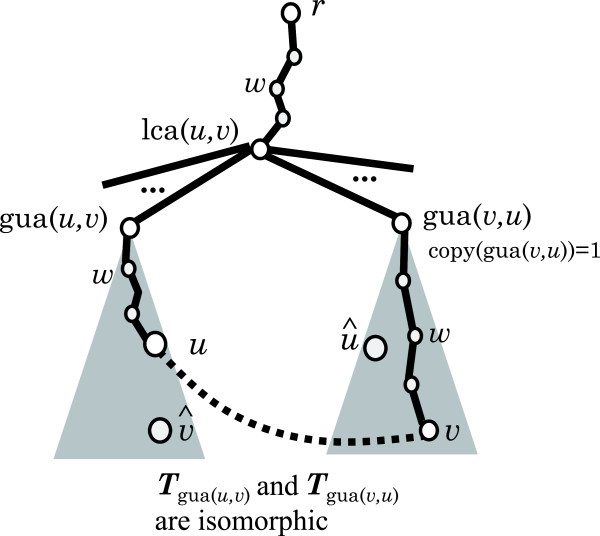
**Illustration of condition (3) for admissible pairs.** The black dotted line joins two vertices *u* and *v*, which will be the edge to create a cycle in the 1-augmented tree *T*+*uv*. Condition (1) and (2) exclude any ancestor *w* of *u* or *v* such that copy(*w*)=1 except for *w*=gua(*v*,*u*). The assumption copy(gua(*v*,*u*))=1 requires the rooted subtree *T*_gua(*u*,*v*)_ to be isomorphic to *T*_gua(*v*,*u*)_ and condition (3)-(i) requires *T*_gua(*u*,*v*)_ to be located immediately on the left of *T*_gua(*v*,*u*)_ among the subtrees rooted at the children of lca(*u*,*v*). Then *T*_gua(*v*,*u*)_ contains a copy û of *u* (i.e., $\text{dfs}(û)=\text{dfs}(u)+|T_{\text{gua}(u,v)}|$). Similarly *T*_gua(*u*,*v*)_ contains a copy $\hat{v}$ of *v* (i.e., $\text{dfs}(\hat{v})=\text{dfs}(v)-|T_{\text{gua}(u,v)}|$). Condition (3)-(ii) requires dfs(û)≤dfs(v) (otherwise we can prove that $\left(\hat{v},û\right)$ is a lexicographically smaller pair such that $T+\hat{v}û$ is isomorphic to *T*+*uv*). Although *T*_gua(*u*,*v*)_ and *T*_gua(*v*,*u*)_ are isomorphic to each other, only paths and nodes relevant for explanation are shown in this figure.

We call a pair of nonadjacent vertices *u*,*v*∈*V*(*T*) with dfs(*u*)<dfs(*v*)*admissible* if it satisfies the following conditions (see Figure 9 for conditions (1) and (2) and Figure 10 for condition (3)): 

● copy(*w*) = 0 for all vertices *w*∈ *V*(*P*(lca(*u*,*v*),*r*))−{*r*};

● copy(*w*)=0 for all vertices *w*∈*V*(*P*(*u*,gua(*u*,*v*)))∪*V*(*P*(*v*,gua(*v*,*u*)))−{lca(*u*,*v*),gua(*v*,*u*)};

● if copy(gua(*v*,*u*))=1 then 

gua(*u*,*v*)=left(gua(*v*,*u*)) (hence *u*≠lca(*u*,*v*)); and

For the copy û of vertex *u* in *T*_gua(*v*,*u*)_, it holds dfs(v)≥dfs(û) (where $\text{dfs}(û)=\text{dfs}(u)+|V(T_{\text{gua}(u,v)})|$).

Note that (3)-(i) implies that copy(gua(*v*,*u*)) in (2) needs to be 0 when lca(*u*,*v*)=*u*.

The next lemma indicates that we only need to add an edge between each admissible pair to avoid duplication of 1-augmented trees, where the proof is given in Additional file 1: S1.3.

##### **Lemma3**.

For a left-heavy tree *T* rooted at its unicentroid *c*_*T*_=*r*, let $G_{T}=\{T+\text{uv}|$ admissible pairs *u*,*v*∈*V*(*T*)}. Then the 1-augmented tree *T*+*uv* for any pair of nonadjacent vertices *u*,*v*∈*V*(*T*) is isomorphic to exactly one 1-augmented tree *G* in $G_{T}$.

#### Algorithm

In this section, we describe an algorithm of the second stage to generate all children of a given multi-tree *T* without duplication and omission, and show the computational complexity of the second stage. To generate all children of *T*, we first find all admissible pairs (*u*,*v*) for *T* and test whether *T* is the parent *π*(*T*+*uv*) of *T*+*uv* or not. Notice that a straightforward method would take *O*(*n*) time to check whether a pair (*u*,*v*) is admissible or not. Since there are at most _*n*_*C*_2_ vertex pairs in a multi-tree *T*, finding all admissible pairs for *T* may take *O*(*n*^3^) time. That is, from Lemma 1, we may need *O*(*n*^3^|*V*(*C*)|^2^)=*O*(*n*^5^) time to generate all children of *T*.

**Figure 11:**
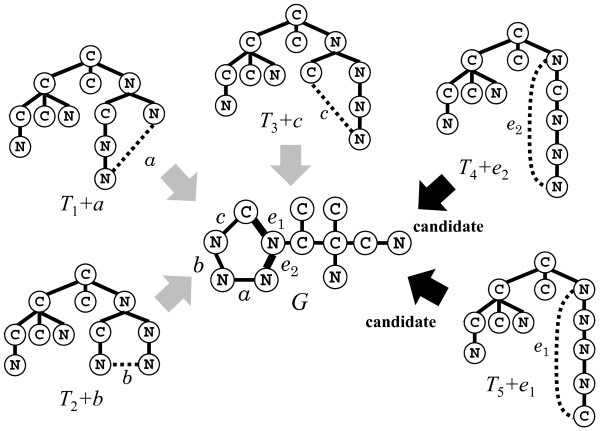
**Illustration of candidate pairs.** A graph *G* will be created by adding each edge shown by a black dotted line. However, no vertex pair for any of edges *a*,*b* and *c* is a candidate pair. We do not have to add edges *a*,*b*, and *c* to *T*_1_,*T*_2_, and *T*_3_, respectively because neither *G*−*a*, *G*−*b*, nor *G*−*c* can be the parent of *G*.

In what follows, we design a faster *O*(*n*^4^)-time algorithm to generate all children of a given multi-tree *T*. For this, we find only a subset of all admissible pairs, called the set of “candidate” pairs defined as follows (see also Figure 11). We see that no pair (*x*,*y*) generates a child *T*+*xy* of *T* if a heavy edge is created in *T*+*xy* and *x* is not an ancestor of *y*, since such (*x*,*y*) does not satisfy any of Cases I and II for generating children of *T*. Hence we are not interested in storing such pairs (*x*,*y*), and call an admissible pair (*x*,*y*) a *candidate* pair when (i) no heavy edge is created in *T*+*xy*; or (ii) *x* is an ancestor of *y*, where (i) (resp., (ii)) is a necessary condition of Case I (resp., Case II). By definition, every candidate vertex pair (*x*,*y*) is admissible, whereas any admissible pair (*x*,*y*) such that *T*+*xy* is a child of *T* is always a candidate pair. Therefore, to generate all children of *T*, we do not need to find all admissible pairs and only have to extract all candidate pairs.

To facilitate this, we examine all vertex pairs (*u*,*v*) (dfs(*u*)<dfs(*v*)) in *T* in a lexicographical order with respect to (dfs(*u*),dfs(*v*)), i.e., we choose each vertex *v*_*i*_ from *v*_0_ to *v*_*n*−1_ as *u* and then choose each vertex *v*_*j*_ from *v*_*i*+1_ to *v*_*n*−1_ as *v*. We call the lexicographical order over vertex pairs a *dfs order*. For each of the generated vertex pairs, we check whether it is a candidate pair or not. Finally, for each candidate pair (*u*,*v*), we test whether *T*+*uv* is a child of *T* in Case I or II.

We can find all candidate pairs for a multi-tree *T* in *O*(*n*^2^) time in total as stated below. The proof is given in Additional file 1: S1.4.

##### **Lemma4**.

For a left-heavy multi-tree *T*, all candidate pairs of *T* can be found in *O*(*n*^2^) time.

Finally, by Lemma 1 and Lemma 4, we can generate all children of a multi-tree *T* in *O*(*n*^4^) time, as stated in the next lemma.

##### **Lemma5**.

Given a left-heavy multi-tree *T*, all children of *T* can be generated in *O*(*n*^4^) time.

##### *Proof*.

For a left-heavy multi-tree *T*, we can find all candidate pairs in *O*(*n*^2^) time by Lemma 4. For each candidate pair (*u*,*v*), we can test whether *T*=*π*(*T*+*uv*) or not in *O*(|*V*(*C*)|^2^) time by Lemma 1. Thus, for a left-heavy multi-tree *T*, all children of *T* can be generated in *O*(*n*^2^|*V*(*C*)|^2^)=*O*(*n*^4^) time. □

## Experimental and results

This section reports experimental results of our algorithm enumerating 1-augmented trees. Tests were carried out on a PC with an Intel Core i5 processor running at 3.20 GHz and the Linux operating system using the C language, employing instances based on chemical compounds selected from the KEGG LIGAND database [15] (*http://www.genome.jp/ligand/*).

(I) First we select four chemical compounds “C00062,” “C03343,” “C03690,” and “C07178” as chemical graphs with 0-augmented tree (acyclic) structure and four chemical compounds “C00095,” “C00270,” “C00645,” and “C00837” as chemical graphs with 1-augmented tree structure (see Additional file 1: Figure S21 for illustrations of these chemical graphs), wherein each benzene ring in chemical compounds “C03343,” “C03690,” and “C07178” is regarded as a virtual atom b of valence 6. These compounds are heuristically selected based on the following criteria: (i) each compound is a 0-augmented tree or 1-augmented tree (except benzene ri ngs), (ii) each compound consists of C,O,H (or, C,O,N,H) atoms, (iii) compounds are not very similar to each other, and (iv) compounds have varying sizes but are not too large.

The virtual atom b is treated as one atom so that we discard all possible regioisomers of benzene. Thus in our experiment, we consider the cycles not caused by benzenes but by other substructures in these 1-augmented trees. We remark that an efficient algorithm has been developed for generating all possible regioisomers of a given 0-augmented tree structure with virtual atoms b by Li *et al*. [36], and an implementation of the algorithm is available on a web server (*http://sunflower.kuicr.kyoto-u.ac.jp/tools/enumol2/*).

To generate problem instances from each of the selected chemical graphs, we define $w\in {\mathbb{Z}}_{+}$ to be a *width* between upper and lower feature vectors. From the feature vector *g*=*f*_*K*_(*G*) of a chemical graph *G* at level *K*, we construct two feature vectors *g*_*U*_ and *g*_*L*_ of width *w* as follows. For each entry *t* with *g*[ *t*]≥1, let *g*_*U*_[ *t*]=*g*[ *t*]+*w* and *g*_*L*_[ *t*]= max{*g*[ *t*]−*w*,0}; and for each entry *t* with *g*[ *t*]=0, let *g*_*U*_[ *t*]=*g*_*L*_[ *t*]=0. See Additional file 1: Figure S23 (resp., Additional file 1: Figure S24) for the lower and upper feature vectors *g*_*L*_ and *g*_*U*_ with *K*=1 and *w*=1 (resp., *K*=2 and *w*=1) created from C00062.

To examine the computational efficiency, we compare the time per output multi-tree/1-augmented tree by our algorithm and by 2-Phase algorithm [14]. Our algorithm enumerates not only the 1-augmented trees in $G_{1}(\;g_{L},g_{U})$ but also the multi-trees in $G_{0}(\;g_{L},g_{U})$. Therefore, if time per output graph of our algorithm is close to that of 2-Phase algorithm, then we can enumerate 1-augmented trees as fast as 2-Phase algorithm enumerates multi-trees.

Table 1 shows the result of the comparison of 2-Phase algorithm and our algorithm for varying *K* with fixed *w*=1, where the meanings of columns are as follows.

**Table 1:** Comparison of 2-Phase algorithm and our algorithm in chemical graphs (I)

**Entry**			**2-Phase Algorithm**			**Our Algorithm**				
**Formula**	***n***	***K***	**Tree**	**Time**	**Time/graph**	**Tree**	**Cycle**	**Time**	**Time/graph**	**Ratio**
		1	388,192	0.288	741.9E-9	388,192	1,786,467	14.056	6.5E-6	8.7
		2	614	0.021	34.2E-6	614	229	0.134	159.0E-6	4.6
C00062		3	95	0.020	210.5E-6	95	0	0.039	410.5E-6	2.0
C_6_*H*_14_N_2_O_4_	12	4	1	0.008	8.0E-3	1	0	0.018	18.0E-3	2.3
		5	1	0.005	5.0E-3	1	0	0.006	6.0E-3	1.2
		6	1	0.006	6.0E-3	1	0	0.006	6.0E-3	1.0
		7	1	0.004	4.0E-3	1	0	0.004	4.0E-3	1.0
		1	1,708	0.007	4.1E-6	1,708	12,626	0.050	3.5E-6	0.9
		2	50	0.004	80.0E-6	50	1,085	0.043	37.9E-6	0.5
C00095		3	0	0.046	–	0	286	0.046	160.8E-6	–
C_6_H_12_O_6_	12	4	0	0.006	–	0	19	0.035	1.8E-3	–
		5	0	0.004	–	0	7	0.030	4.3E-3	–
		6	0	0.004	–	0	5	0.028	5.6E-3	–
		7	0	0.004	–	0	5	0.013	2.6E-3	–
		1	5,446,987	7.690	1.4E-6	5,446,987	31,395,098	217.681	5.9E-6	4.2
		2	373	0.022	59.0E-6	373	71	0.171	385.1E-6	6.5
C03343		3	187	0.023	123.0E-6	187	25	0.071	334.9E-6	2.7
C_16_H_22_O_4_	15	4	101	0.022	217.8E-6	101	9	0.042	381.8E-6	1.8
		5	51	0.022	431.4E-6	51	6	0.059	1.0E-3	2.4
		6	43	0.009	209.3E-6	43	0	0.036	837.2E-6	4.0
		7	28	0.013	464.3E-6	28	0	0.020	714.3E-6	1.5
		1	2,926,382	2.878	983.5E-9	2,926,382	23,965,432	146.669	5.5E-6	5.5
		2	41,468	1.035	25.0E-6	41,468	213,820	37.792	148.0E-6	5.9
C00645		3	491	0.562	1.1E-3	491	4,482	6.281	1.3E-3	1.1
C_8_H_15_NO_6_	15	4	0	0.523	–	0	73	4.168	57.1E-3	–
		5	0	0.374	–	0	5	2.320	464.0E-3	–
		6	0	0.135	–	0	3	0.430	143.3E-3	–
		7	0	0.121	–	0	1	0.328	328.0E-3	–
		1	167,172,180	238.554	1.4E-6	≥ 1,594,520	≥ 33,962,677	T. O.	50.7E-6	35.5
		2	210	1.232	5.9E-3	210	641	1.888	2.2E-3	0.4
C00837		3	0	0.853	–	0	4	1.206	301.5E-3	–
C_8_H_18_N_6_O_4_	18	4	0	0.445	–	0	2	0.523	261.5E-3	–
		5	0	0.389	–	0	1	0.596	596.0E-3	–
		6	0	0.298	–	0	1	0.367	367.0E-3	–
		7	0	0.285	–	0	1	0.395	395.0E-3	–
		1	62,234,720	321.155	5.2E-6	≥ 4,812,773	≥ 40,426,928	T. O.	39.8E-6	7.7
		2	884	0.310	350.7E-6	884	180	1.824	1.7E-3	4.9
C07178		3	22	0.026	1.2E-3	22	4	0.099	3.8E-3	3.2
C_21_H_28_N_2_O_5_	18	4	1	0.004	4.0E-3	1	0	0.005	5.0E-3	1.3
		5	1	0.004	4.0E-3	1	0	0.005	5.0E-3	1.3
		6	1	0.004	4.0E-3	1	0	0.005	5.0E-3	1.3
		7	1	0.005	5.0E-3	1	0	0.005	5.0E-3	1.0
		1	≥ 1,208,446,991	T. O.	1.5E-6	≥ 7,009,856	≥ 47,008	T. O.	255.1E-6	171.2
		2	27,312,856	965.131	35.3E-6	≥ 337,989	≥ 3,593,865	T. O.	458.1E-6	13.0
C00270		3	156,073	391.611	2.5E-3	≥ 1,546	≥ 234,187	T. O.	7.6E-3	3.0
C_11_H_19_NO_9_	21	4	0	299.393	–	≥ 0	≥ 165	T. O.	10.9E+0	–
		5	0	208.268	–	≥ 0	≥ 7	T. O.	257.1E+0	–
		6	0	19.361	–	0	2	85.109	42.6E+0	–
		7	0	9.720	–	0	2	30.301	15.2E+0	–
		1	≥ 664,049,939	T. O.	2.7E-6	≥ 4,621,297	≥ 33,216,732	T. O.	47.6E-6	17.5
		2	164,885	34.357	208.4E-6	164,885	1,425	213.810	1.3E-3	6.2
C03690		3	32,995	15.978	484.3E-6	32,995	179	66.612	2.0E-3	4.1
C_24_H_38_O_4_	23	4	3,884	2.265	583.2E-6	3,884	17	5.383	1.4E-3	2.4
		5	1,237	1.490	1.2E-3	1,237	13	3.466	2.8E-3	2.3
		6	559	0.773	1.4E-3	559	0	1.554	2.8E-3	2.0
		7	177	0.445	2.5E-3	177	0	0.617	3.5E-3	1.4

Note on tables: 

(1) C00062, C00095, C00270 C00645, C00837, C03343, C07178, and C03690 are the chemical compounds in the KEGG LIGAND database, respectively;

(2) in Table 1, the width for constructing upper and lower feature vectors is 1;

(3) *n* is the number of vertices without hydrogen atoms in an instance preprocessed by replacing each benzene ring with a new atom with six valences;

(4) *w* is the width for constructing upper and lower feature vectors;

(5) *K* is the level of given feature vectors;

(6) “time (s)” is the CPU time in seconds;

(7) T.O. means the “time over” (the time limit is set to be 1,800 seconds);

(8) “time/graph” is the time per enumerating one graph;

(9) “tree” is the number of all possible solutions of tree-like chemical graph in the time limit;

(10) “cycle” is the number of all possible solutions of 1-tree chemical graph in the time limit;

(11) “ratio” is a number such that “time/graph” of our algorithm is divided by that of 2-Phase algorithm;

(12) for any real numbers x and y, let *x*E*y* denote *x*×10 ^*y*^.

It is to be noted that in some instances, the number of enumerated trees by 2-Phase algorithm and that of our algorithm are different because of the time limit. Hence, the “tree” and “cycle” columns show the number of incomplete solutions in instances whose “time” column is “T.O.”. However, this is not a critical issue because we mainly want to know the “time per graph” and its “ratio” between 2-Phase algorithm and our algorithm. We can make use of them as beneficial results from “tree,” “cycle,” and “time” columns even if they are incomplete and “T.O.”.

We find that almost all instances solved within the time limit by 2-Phase algorithm are also solved by our algorithm within the time limit. Moreover, the “ratio” of instances is less than 10 except 4 out of 38 cases, and that of many instances is less than 5. This means that the time per output by our algorithm is close to that by 2-Phase algorithm. Therefore, we have demonstrated that our algorithm maintains the high computational efficiency of 2-Phase algorithm even if *K* changes. Note that our algorithm does not output any 1-augmented trees in $G_{1}(\;g_{L},g_{U})$ in “C00062,” “C03343,” “C07178,” and “C03690” when *K* is large. This is because the instances are acyclic chemical compounds: 1-augmented trees become less able to satisfy the feature vector constraint as *K* increases and only multi-trees can satisfy the feature vector constraint.

Table 2 shows the result of the comparison of 2-Phase algorithm and our algorithm for varying *w* with fixed *K*=3. Just like with Table 1, almost all instances solved within the time limit by 2-Phase algorithm are also solved by our algorithm within the time limit. The “ratio” of instances is less than 10 except 7 out of 48 cases, and that of many instances is less than 5. In particular, with respect to “C00095,” “C00645,” and “C00837,” which have 1-augmented tree structure, the “ratio” is less than 1 or close to 1. This implies that our algorithm can enumerate 1-augmented trees and multi-trees faster than 2-Phase algorithm enumerates multi-trees. These results mean that the time per output by our algorithm is close to that by 2-Phase algorithm. Therefore, we have demonstrated that our algorithm maintains the high computational efficiency of 2-Phase algorithm even if *w* changes.

**Table 2:** **Comparison of varying width *w * in chemical graphs (I)**

**Entry**				**2-Phase Algorithm**			**Our Algorithm**				
**Formula**	***n***	***w***	***K***	**Tree**	**Time**	**Time/graph**	**Tree**	**Cycle**	**Time**	**Time/graph**	**Ratio**
		1	3	95	0.020	210.5E-6	95	0	0.039	410.5E-6	2.0
		2	3	862	0.020	23.2E-6	862	100	0.130	135.1E-6	5.8
C00062		3	3	2,531	0.030	11.8E-6	2,531	894	0.213	62.2E-6	5.3
C_6_H_14_N_2_O_4_	12	4	3	3,611	0.044	12.2E-6	3,611	2,737	0.254	40.0E-6	3.3
		5	3	4,438	0.044	9.9E-6	4,438	5,454	0.265	26.8E-6	2.7
		50	3	5,138	0.045	8.8E-6	5,138	12,044	0.388	22.6E-6	2.6
		1	3	0	0.005	–	0	286	0.046	160.8E-6	–
		2	3	40	0.065	1.6E-3	40	1,569	0.065	40.4E-6	0.0
C00095		3	3	280	0.009	32.1E-6	280	4,899	0.089	17.2E-6	0.5
C_6_H_12_O_6_	12	4	3	855	0.010	11.7E-6	855	8,273	0.148	16.2E-6	1.4
		5	3	1,502	0.011	7.3E-6	1,502	12,085	0.177	13.0E-6	1.8
		50	3	4,608	0.012	2.6E-6	4,608	23,686	0.186	6.6E-6	2.5
		1	3	187	0.023	123.0E-6	187	25	0.071	334.9E-6	2.7
		2	3	2,077	0.091	43.8E-6	2,077	1,251	0.880	264.4E-6	6.0
C03343		3	3	5,345	0.201	37.6E-6	5,345	5,746	3.134	282.6E-6	7.5
C_16_H_22_O_4_	15	4	3	10,391	0.346	33.3E-6	10,391	16,912	4.041	148.0E-6	4.4
		5	3	14,531	0.482	33.2E-6	14,531	33,064	5.887	123.7E-6	3.7
		50	3	19,819	0.655	33.0E-6	19,819	94,725	7.833	68.4E-6	2.1
		1	3	491	0.562	1.1E-3	491	4,482	6.281	1.3E-3	1.1
		2	3	7,846	1.122	143.0E-6	7,846	76,261	17.199	204.5E-6	1.4
C00645		3	3	151,227	2.420	16.0E-6	151,227	716,216	39.476	45.5E-6	2.8
C_8_H_15_NO_6_	15	4	3	216,507	2.946	13.6E-6	216,507	1,270,462	33.842	22.8E-6	1.7
		5	3	272,898	3.405	12.5E-6	272,898	1,757,010	40.323	19.9E-6	1.6
		50	3	355,958	3.985	11.2E-6	355,958	2,625,154	43.002	14.4E-6	1.3
		1	3	0	0.853	–	0	4	1.206	301.5E-3	–
		2	3	389	1.569	4.0E-3	389	660	2.496	2.4E-3	0.6
C00837		3	3	2,510	1.999	796.4E-6	2,510	3,173	3.367	592.5E-6	0.7
C_8_H_18_N_6_O_4_	18	4	3	8,544	2.314	270.8E-6	8,544	12,834	3.994	186.8E-6	0.7
		5	3	13,796	2.465	178.7E-6	13,796	27,186	4.841	118.1E-6	0.7
		50	3	24,313	2.683	110.4E-6	24,313	94,089	5.540	46.8E-6	0.4
		1	3	22	0.026	1.2E-3	22	4	0.099	3.8E-3	3.2
		2	3	386	0.058	150.3E-6	386	261	0.426	658.4E-6	4.4
C07178		3	3	2,376	0.089	37.5E-6	2,376	1,288	0.735	200.6E-6	5.4
C_21_H_28_N2O_5_	18	4	3	4,092	0.102	24.9E-6	4,092	2,240	0.863	136.3E-6	5.5
		5	3	4,629	0.109	23.5E-6	4,629	2,385	1.284	183.1E-6	7.8
		50	3	5,103	0.115	22.5E-6	5,103	2,603	0.980	127.2E-6	5.6
		1	3	156,073	391.611	2.5E-3	≥ 1,546	≥ 234,187	T. O.	7.6E-3	3.0
		2	3	12,515,364	1331.770	106.4E-6	≥ 93,244	≥ 1,107,707	T. O.	1.5E-3	14.1
C00270		3	3	≥ 88,182,895	T. O.	20.4E-6	≥ 4,350,635	≥ 1,135,547	T. O.	328.3E-6	16.1
C_11_H_19_NO_9_	21	4	3	≥ 134,281,382	T. O.	13.4E-6	≥ 5,230,515	≥ 2,086,287	T. O.	246.0E-6	18.4
		5	3	≥ 169,965,948	T. O.	10.6E-6	≥ 5,213,010	≥ 2,383,696	T. O.	237.1E-6	22.4
		50	3	≥ 254,637,067	T. O.	7.1E-6	≥ 7,025,893	≥ 12,785,700	T. O.	90.9E-6	12.9
		1	3	32,995	15.978	484.3E-6	32,995	179	66.612	2.0E-3	4.1
		2	3	2,472,133	149.048	60.3E-6	≥ 1,763,123	≥ 702,493	T. O.	730.0E-6	12.1
C03690		3	3	13,120,833	509.010	38.8E-6	≥ 2,416,279	≥ 2,028,470	T. O.	405.0E-6	10.4
C_24_H_38_O_4_	23	4	3	43,379,162	1289.269	29.7E-6	≥ 1,815,035	≥ 4,297,658	T. O.	294.5E-6	9.9
		5	3	≥ 80,447,027	T. O.	22.4E-6	≥ 2,828,014	≥ 10,431,681	T. O.	135.7E-6	6.1
		50	3	≥ 111,576,848	T. O.	16.1E-6	≥ 3,580,958	≥ 55,327,406	T. O.	30.6E-6	1.9

Finally, from Table 1 and Table 2, we compare 2-Phase algorithm and our algorithm in terms of varying *n*, where *n* is the size of an instance. Note that *n* is the number of vertices without hydrogen atoms in an instance preprocessed by replacing each benzene ring with a new atom with six valences. We notice that there is no large difference in the “ratio” between all cases in spite of the fact that the instance size of C03690 is almost twice as large as that of C00062. This implies that our algorithm maintains the high computational efficiency of 2-Phase algorithm even if the instance size becomes large.

(II) Next we select four chemical compounds, prostaglandin (D08040), allobarbital (D00332), gabapentin (D00555), and histamine (D00079) as chemical graphs with 1-augmented tree structure (see Additional file 1: Figure S22 for illustrations of these chemical graphs), all of which are existing drug compounds. We conducted the same experiment as we did for (I): Table 3 shows the result of the comparison of 2-Phase algorithm and our algorithm for varying *K* with fixed *w*=1; Table 4 shows the result of the comparison of 2-Phase algorithm and our algorithm for varying *w* with fixed *K*=3. In this experiment, we observe that there still is no large difference in the “ratio” between all cases except for the instance of D00079.

**Table 3:** Comparison of 2-Phase algorithm and our algorithm in chemical graphs (II)

**Entry**			**2-Phase Algorithm**			**Our Algorithm**				
**Formula**	***n***	***K***	**Tree**	**Time**	**Time/graph**	**Tree**	**Cycle**	**Time**	**Time/graph**	**Ratio**
		1	2,609	0.006	2.3E-6	2,609	11,263	0.036	2.6E-6	1.1
		2	193	0.007	36.3E-6	193	165	0.015	41.9E-6	1.2
D08040		3	14	0.009	642.9E-6	14	5	0.014	736.8E-6	1.1
C_5_H_9_N_3_	8	4	9	0.005	555.6E-6	9	2	0.006	545.5E-6	1.0
		5	4	0.004	1.0E-3	4	1	0.005	1.0E-3	1.0
		6	4	0.004	1.0E-3	4	1	0.005	1.0E-3	1.0
		7	1	0.005	1.3E-3	4	1	0.006	1.2E-3	0.9
		1	17,470	0.127	7.2E-6	17,470	264,326	1.446	5.1E-6	0.7
		2	1,183	0.023	19.4E-6	1,183	16,233	0.294	17.9E-6	0.9
D00332		3	30	0.017	566.7E-6	30	1,318	0.170	126.1E-6	0.2
C_9_H_17_NO_2_	12	4	0	0.025	–	0	292	0.112	383.6E-6	–
		5	0	0.034	–	0	41	0.090	2.2E-3	–
		6	0	0.021	–	0	12	0.050	4.1E-3	–
		7	0	0.016	–	0	8	0.037	4.6E-3	–
		1	54,072,616	200.647	3.7E-6	≥11,645,178	≥110,673,601	T.O.	14.7E-6	4.0
		2	68,253	5.458	80.0E-6	68,253	321,853	130.500	334.2E-6	4.2
D00555		3	4,590	4.115	896.5E-6	4,590	6,511	70.821	6.4E-3	7.1
C_11_H_18_N_2_O_3_	16	4	91	2.702	29.7E-3	91	278	28.632	77.5E-3	2.6
		5	0	1.438	–	0	38	15.129	397.3E-3	–
		6	0	0.694	–	0	5	6.882	1.4E+0	–
		7	0	0.211	–	0	1	0.663	663.0E-3	–
		1	≥10,280	T.O.	175.1E-3	≥1,432	≥883,812	T.O.	2.0E-3	0.0
		2	≥19,587,838	T.O.	91.9E-6	≥0	≥0	T.O.	–	–
D00079		3	≥1,134,806	T.O.	1.6E-3	≥21,048	≥0	T.O.	85.5E-3	53.4
C_20_H_32_*O*_5_	25	4	≥17,852	T.O.	100.8E-3	≥0	≥0	T.O.	–	–
		5	≥23	T.O.	78.3E+0	≥0	≥0	T.O.	–	–
		6	≥0	T.O.	–	≥0	≥0	T.O.	–	–
		7	≥0	T.O.	–	≥0	≥0	T.O.	–	–

**Table 4:** **Comparison of varying width *w * in chemical graphs (II)**

**Entry**				**2-Phase Algorithm**			**Our Algorithm**				
**Formula**	***n***	***w***	***K***	**Tree**	**Time**	**Time/graph**	**Tree**	**Cycle**	**Time**	**Time/graph**	**Ratio**
		1	3	14	0.009	643.9E-6	14	5	0.014	736.8E-6	1.1
		2	3	49	0.009	183.7E-6	49	14	0.017	269.8E-6	1.5
D08040		3	3	58	0.009	155.2E-6	58	21	0.017	215.1E-6	1.4
C_5_H_9_N_3_	8	4	3	60	0.009	150.0E-6	60	25	0.017	200.0E-6	1.3
		5	3	60	0.009	150.0E-6	60	26	0.017	197.6E-6	1.3
		50	3	61	0.003	49.2E-6	61	28	0.017	191.0E-6	3.9
		1	3	30	0.017	566.7E-6	30	1,318	0.170	126.1E-6	0.2
		2	3	313	0.024	76.7E-6	313	8,822	0.266	29.1E-6	0.4
D00332		3	3	1,327	0.024	76.7E-6	1,327	18,010	0.285	14.7E-6	0.8
C_9_*H*17NO_2_	12	4	3	2,239	0.025	11.2E-6	2,239	24,550	0.293	10.9E-6	1.0
		5	3	4,197	0.025	6.0E-6	4,197	30,122	0.297	8.7E-6	1.5
		50	3	6,656	0.025	3.8E-6	6,656	34,145	0.309	7.6E-6	2.0
		1	3	4,590	4.115	896.5E-6	4,590	6,511	70.806	6.4E-3	3.8
		2	3	76,901	10.466	136.1E-6	76,901	186,971	221.353	838.7E-6	6.2
D00555		3	3	221,492	14.952	67.5E-6	221,492	770,625	317.488	320.0E-6	4.7
C_11_H_18_N2O_3_	16	4	3	348,335	16.381	47.0E-3	348,335	1,307,167	347.379	209.8E-6	4.5
		5	3	458,635	16.837	36.7E-3	458,635	1,976,544	357.252	146.7E-6	4.0
		50	3	556,272	17.090	30.7E-3	556,272	3,544,713	363.743	88.7E-6	2.9
		1	3	≥1,134,806	T.O.	1.6E-3	≥21,048	≥0	T.O.	85.5E-3	53.4
		2	3	≥3,917,059	T.O.	459.5E-6	≥0	≥0	T.O.	–	–
D00079		3	3	≥86,360	T.O.	20.8E-3	≥28,187	≥0	T.O.	64.6E-3	3.1
C_20_H_32_O_5_	25	4	3	≥1,469,428	T.O.	1.2E-3	≥61,929	≥229	T.O.	29.0E-3	24.2
		5	3	≥5,118,134	T.O.	351.7E-6	≥19,900	≥1,726	T.O.	83.2E-3	236.6
		50	3	≥216,008,008	T.O.	8.3E-6	≥0	≥0	T.O.	–	–

## Discussions and conclusions

We considered the problem of enumerating all chemical graphs of 1-augmented tree structure from a given set of path-frequency based feature vectors specified by upper and lower feature vectors, and proposed a new exact algorithm by extending 2-Phase algorithm [14]. The experimental results reveal that the computational efficiency of the new algorithm remains high, considering the hardness of treating 1-augmented trees compared with 0-augmented trees.

One of our future works is to introduce new bounding operations for 1-augmented trees in 2-Phase algorithm and our procedure for creating a cycle. Additionally, it is important to extend the proposed algorithm for enumeration of *k*-augmented trees with *k*≥2 because we can cover 59*%*, 79*%*, and 90*%* of chemical compounds by 2-augmented trees, 3-augmented trees, and 4-augmented trees, respectively. In this paper, we used the assumption that chemical graphs we treat contain only atoms with valence at most 4 (except benzene rings) in order to define the parent of a 1-augmented tree *G* as a 0-augmented tree *T* that is obtained by removing an edge corresponding to a single bond in *G*. However, it is not difficult to extend our enumeration algorithm for chemical graphs possibly with atoms with valence more than 4 just by modifying the definition so that the parent of a 1-augmented tree *G* is allowed to be a 0-augmented tree *T* obtained by removing an edge that corresponds to a double or triple bond in *G*. Although benzene rings have already been treated as virtual atoms of valence 6, regioisomers are ignored in the proposed algorithm. As mentioned in “Experimental and results” section, an efficient algorithm for generating all possible regioisomers of a given 0-augmented tree structure with virtual atoms b has been developed [36]. Therefore, combination of the proposed algorithm with that algorithm is left as future work as well as further extensions for including atoms with valence more than 4 and furan and more general structures.

Although we do not aim to develop enumeration algorithms that are directly applicable to drug design, this is a future target of our research. In order to apply enumeration algorithms to drug design, considering features based on the path frequency is far from sufficient. Factors such as hydrogen bond donors, hydrogen bond acceptors, positive charges, negative charges, and hydrophobic centers should be taken into account. In addition, the binding site information of the target molecule and geometric information such as the occurrence of rotatable bonds should be reflected. In order to include these factors in enumeration algorithms, we should develop efficient methods that can relate chemical graphs with such physico-chemical and geometric factors. However, such a development is not an easy task even for one type of factor and thus is long-term future work.

## Competing interests

The authors declare that they have no competing interests.

## Authors’ contributions

HN designed the algorithm and performed theoretical analysis on it. MS detailed the algorithm, implemented it, and performed computational experiments. TA participated in discussions for analyses of the algorithm and the results of computational experiments. HN and MS wrote the manuscript and TA revised it. All authors read and approved the final manuscript.

## Supplementary Material

Additional file 1 **Proofs of Lemmas and Theorems.** Proofs of Lemma 1, Theorem 2, Lemma 3, and Lemma 4, descriptions of the 2-phase algorithm and the main algorithm, and some figures for chemical graphs and upper and lower feature vectors used in the computational experiment are given in this supplemen Click here for file
