# Supplementary material for: Efficient enumeration of monocyclic chemical graphs with given path frequencies
Source: J Cheminform. 2014 May 30;6:31. doi: 10.1186/1758-2946-6-31 (PMC4049473; doi:10.1186/1758-2946-6-31)
Supplement: Additional file 1 — Proofs of Lemmas and Theorems. Proofs of Lemma 1, Theorem 2, Lemma 3, and Lemma 4, descriptions of the 2-phase algorithm and the main algorithm, and some figures for chemical graphs and upper and lower feature vectors used in the computational experiment are given in this supplemen [file 1758-2946-6-31-S1.pdf]

Supplementary information
Efficient Enumeration of Monocyclic Chemical Graphs with Given Path Frequencies

Masaki Suzuki, Hiroshi Nagamochi

Graduate School of Informatics,
Kyoto University
{m_suzuki,nag}@amp.i.kyoto-u.ac.jp

Tatsuya Akutsu

Bioinformatics Center,
Institute for Chemical Research,
Kyoto University
takutsu@kuicr.kyoto-u.ac.jp

S1 Proof of Lemmas and Theorems

S1.1 Proof of Lemma 1

Proof: Case I. C contains the root (centroid) of T: Since the centroid is in C, the 1-augmented tree $G = T + xy$ has no heavy edge incident to C and we check if $\pi(G) = T$ according to the definition of parent in Case 1. Then we compute $\sigma^*(v)$ for all vertices $v \in V(T)$ and $\sigma^*(e)$ for all simple edges e in C, as discussed in the definition of parent π in Case 1. If $xy \in E^*(C)$ then $T + xy$ is a child of T; otherwise $T + xy$ is not a child of T.

To compute σ^*, we need to know the signature $\sigma(T_w)$ of each tree T_w with $w \in N(v)$ and $v \in V(T)$. Since C contains the root of T, all these trees T_w appear as subtrees of T rooted at w and we know their signature $\sigma(T_w)$ from the codes $\delta(\tau)$ and $M(\tau)$ on the labeling dfs(τ). With an adequate data structure, we can compute $\sigma(T_w)$ of each tree T_w in $O(1)$ time and testing whether $T = \pi(T+xy)$ can be done in $O(|V(C)|^2)$ time due to verifying that $c^*(xy)$ is the lexicographically maximum code among $c^*(e)$ for at most $|V(C)|$ simple edges e in C, whose length are $2|V(C)|-1$.

Case II. C does not contain the root (centroid) of T: Since the centroid is not in C, $G = T+xy$ has a heavy edge v^*w^* incident to a vertex v^* in C and we check if $\pi(G) = T$ according to the definition of parent in Case 2. In Case 2, only one of the two edges in C incident to v^* is removed from G to define its parent $\pi(G)$. Hence $G = T + xy$ can be a child of T only when xy is incident to v^*, i.e., one of x and y (say x) is equal to v^* and the other y is a descendant of $v^* = x$ (otherwise we conclude that $T + xy$ is not a child of T). If the other edge xz in C incident to $v^* = x$ is not a simple edge, then $T + xy$ is a child of T. When xz is a simple edge, we compute $\sigma^*(xy)$ and $\sigma^*(xz)$ as discussed in the definition of parent π in Case 2. Then $T + xy$ is a child of T if and only if $\sigma^*(xy)$ is not smaller than $\sigma^*(xz)$.

We can compute σ^* in the similar manner of Case I. We here remark that after the first stage, we have not computed the signature σ of any tree T_w with $w \in N(v^*)$ since none of such tree is a subtree rooted at w in the tree T rooted at its centroid. However, such trees are ignored in computing σ^* in Case 2. We can compute $\sigma^*(xy)$ and $\sigma^*(xz)$ in $O(|V(C)|)$ time and testing whether $T = \pi(T + xy)$ can be accomplished in $O(|V(C)|)$ time due to comparing codes $c^*(xy)$ and $c^*(xz)$, whose length are $2|V(C)| - 1$.

The above procedure is described as follows.

Algorithm ChildTest
Input: A rooted left-heavy multi-tree T and a pair (x, y) of nonadjacent vertices in $V(T)$.

Output: "yes" if $T + xy$ is a child; "no" otherwise.

if The cycle C in $T + xy$ contains the root (centroid) of T **then** /* Case I */
  Compute $\sigma^*(v)$ for all vertices $v \in V(T)$ and $\sigma^*(e)$ for all simple edges e in C, as in the definition of parent π in Case 1;
  Return "yes" if $xy \in E^*(C)$ or "no" otherwise
else /* Case II: Now $G = T + xy$ has a heavy edge v^*w^* incident to a vertex v^* in C */
  if xy is not incident to v^* **then** return "no"
  else
    if the other edge xz in C incident to $v^* = x$ is not a simple edge **then** return "yes"
    else /* xz is a simple edge */
      Compute $\sigma^*(xy)$ and $\sigma^*(xz)$ as in the definition of parent π in Case 2;
      if $\sigma^*(xy)$ is not smaller than $\sigma^*(xz)$ **then** return "yes"
      else return "no"
      end if
    end if
  end if
end if □

S1.2 Proof of Theorem 2

Proof: For a tree T and two vertices $x, y \in V(T)$, let $P(x, y)$ denote the unique path in T that connects x and y.

For a vertex in the unique cycle C in a 1-augmented tree G, let G_u denote the component containing u that appears after removing the two edges in C incident to u. Hence G_u is a subtree of G, and we regard it as a subtree rooted at u. Let $\sigma^*(u; C)$ denote the signature of G_u; $\sigma^*(u; C) = \sigma^*(v; C)$ if and only if $G_u \underset{r}{\approx} G_v$. When G is obtained from a tree T by adding an edge uv, the unique cycle in G is denoted by C_{uv}.

lca(u, v) denote the least common ancestor of u and v, i.e., the highest vertex in $P(u, v)$ (in a tree T rooted at a bicentroid $c_T = rr'$, the least common ancestor lca(x, y) of two vertices such that $x \in V(T_r)$ and $y \in V(T_{r'})$ is defined to be the edge $c_T = rr'$). In a rooted tree, a pair of vertices x and y is called *comparable* if one of them is an ancestor of the other. Let dfs be the depth-first search number obtained by traversing T starting from r, where $r = c_T$ or the left endpoint r of $c_T = rr'$.

To prove this theorem, we derive a contradiction assuming that there are pairs of nonadjacent vertices $\{u, v\}, \{u', v'\} \subseteq V(T)$ such that $T + uv \sim T + u'v'$ but $T + uv \underset{r}{\not\approx} T + u'v'$. Among such pairs, we choose $\{u, v\}, \{u', v'\} \subseteq V(T)$ so that $\mathrm{dfs}(u) + \mathrm{dfs}(v) + \mathrm{dfs}(u') + \mathrm{dfs}(v')$ is minimized. Assume without loss of generality that $\mathrm{dfs}(u) < \mathrm{dfs}(v)$, $\mathrm{dfs}(u') < \mathrm{dfs}(v')$ and $\mathrm{dfs}(u) \leq \mathrm{dfs}(u')$. Note that the assumption implies that vertex lca(u, u') has no two children a and a' in T such that

$$u \in V(T_a),\ u' \in V(T_{a'}) \text{ and } T_a \underset{r}{\approx} T_{a'}, \quad (1)$$

where $T + \hat{u}v' \underset{r}{\approx} T + u'v'$ holds for the vertex $\hat{u} \in V(T_a)$ with $\xi(\hat{u}) = u'$ in the rooted-isomorphism ξ from T_a to $T_{a'}$. This is because $T + uv \underset{r}{\not\approx} T + \hat{u}v'$ if and only if $T + uv \underset{r}{\not\approx} T + u'v'$ (by $T + \hat{u}v' \underset{r}{\approx} T + u'v'$) and thereby if (1) holds, then (u, v) and $(\hat{u}, v')$ would have a smaller sum of dfs than that by (u, v) and (u', v'). Similarly lca(v, v') has no two children a and a' in T such that $v \in V(T_a)$, $v' \in V(T_{a'})$ and $T_a \underset{r}{\approx} T_{a'}$.

We first prove that $T + uv \sim T + u'v'$ implies that

$$\mathrm{lca}(u, v) = \mathrm{lca}(u', v') = c_T$$

(note that this implies that $u, u' \in V(T_r)$ and $v, v' \in V(T_{r'})$ when $c_T = rr'$).

Assume indirectly that lca$(u, v) \neq c_T$ (when $c_T = r$) and lca$(u, v) \notin \{r, r'\}$ (when $c_T = rr'$). Then the vertex $a = \mathrm{lca}(u, v)$ has a unique code $\sigma^*(a; C_{uv})$ among all vertices in C_{uv}, since the

subtree G_a of $G = T + uv$ contains c_T and has more than $n/2$ vertices. Hence $\psi(a)$ is a vertex $b \in V(C_{u'v'})$ which has the same code $\sigma^*(a; C_{uv}) = \sigma^*(b; C_{u'v'})$, indicating that the subtree G'_b of $G' = T + u'v'$ must contain c_T. Moreover $\sigma^*(a; C_{uv}) = \sigma^*(b; C_{u'v'})$ means that ψ maps $c_T = r$ (resp., $c_T = rr'$) in $T + uv$ to $c_T = r$ (resp., $c_T = rr'$) in $T + u'v'$, i.e., $T + uv \underset{r}{\approx} T + u'v'$, a contradiction to the assumption on (u, v) and (u', v'). Hence $\mathrm{lca}(u, v) = c_T$ (when $c_T = r$) and $\mathrm{lca}(u, v) \in \{r, r'\}$ (when $c_T = rr'$). Analogously, $\mathrm{lca}(u', v') = c_T$ or $\mathrm{lca}(u', v') \in \{r, r'\}$ (when $c_T = rr'$). When $c_T = r$, this proves that $\mathrm{lca}(u, v) = \mathrm{lca}(u', v') = r = c_T$. Consider the case of $c_T = rr'$ and $\mathrm{lca}(u, v) \neq \mathrm{lca}(u', v')$, i.e., $\mathrm{lca}(u, v) = r$ or $\mathrm{lca}(u', v') = r'$ (since $\mathrm{dfs}(u) \leq \mathrm{dfs}(u')$ and $\mathrm{dfs}(r) < \mathrm{dfs}(r')$). Assume $\mathrm{lca}(u, v) = r$ (the case of $\mathrm{lca}(u', v') = r'$ can be treated analogously). Then vertex $r = \mathrm{lca}(u, v)$ still has a unique code $\sigma^*(r; C_{uv})$ among all vertices in C_{uv}, where G_r contains a subtree (rooted at r') with at least $n/2$ vertices, and $\psi(r)$ must be vertex $r' \in V(C_{u'v'})$ which can have the same code $\sigma^*(r; C_{uv}) = \sigma^*(r'; C_{u'v'})$. Then $\mathrm{lca}(u', v') = r'$ holds (otherwise $G'_{r'}$ cannot contain a subtree with at least $n/2$ vertices). This means that the subtrees T_r and $T_{r'}$ of T are rooted-isomorphic, and $\psi(r) = r'$ and $\psi(r') = r$, i.e., $T + uv \underset{r}{\approx} T + u'v'$, a contradiction to the assumption on (u, v) and (u', v'). This completes the proof of property that $\mathrm{lca}(u, v) = \mathrm{lca}(u', v') = c_T$. Now $\mathrm{lca}(u, u')$ and $\mathrm{lca}(v, v')$ are both on the cycles C_{uv} and $C_{u'v'}$.

In what follows, we show that there are rooted-isomorphic subtrees T_{a^*} and T_{b^*} of T, where a^* and b^* are children of unicentroid $c_T = r$ or $a^* = r$ and $b^* = r'$ of bicentroid $c_T = rr'$, such that

$$\begin{array}{l} u, u' \in V(T_{a^*}),\ v, v' \in V(T_{b^*}) \text{ and} \\ \text{the rooted-isomorphism } \xi \text{ from } T_{a^*} \text{ to } T_{b^*} \text{ satisfies } \xi(u) = v' \text{ and } \xi(u') = v. \end{array} \tag{2}$$

Note that this implies that $T + uv$ and $T + u'v'$ admit a rooted-isomorphism, which contradicts the assumption on (u, v) and (u', v').

Visiting C_{uv} (resp., $C_{u'v'}$) in the clockwise order means to traverse the cycle starting from $\mathrm{lca}(u, u')$, visiting the ancestors of $\mathrm{lca}(u, u')$ and then visiting the rest of vertices in the cycle. Let $\sigma^*(C_{uv}) = (\sigma^*(t_1; C_{uv}), \sigma^*(t_2; C_{uv}), \ldots, \sigma^*(t_c; C_{uv}))$ be the sequence of codes of vertices in C_{uv} that appear from the first vertex $t_1 = \mathrm{lca}(u, u')$ to the last vertex t_c ($c = |V(C_{uv})| = |V(C_{u'v'})|$) in the clockwise order. Let $\sigma^*(C_{u'v'}) = (\sigma^*(t'_1; C_{u'v'}), \sigma^*(t'_2; C_{u'v'}), \ldots, \sigma^*(t'_c; C_{u'v'}))$ be the sequence of codes of vertices in $C_{u'v'}$ that appear from the first vertex $t'_1 = \mathrm{lca}(u, u')$ to the last vertex t'_c in the clockwise order.

The isomorphism ψ from $G = T + uv$ to $G' = T + u'v'$ maps $\sigma^*(C_{uv})$ to $\sigma^*(C_{u'v'})$ in one of the following ways: (i) rotate $\sigma^*(C_{uv})$ by some amount $0 \leq s < c$ in the clockwise order to get $\sigma^*(C_{uv})$; or (ii) flip $\sigma^*(C_{uv})$ at t_1 and then rotate the flipped sequence by some amount $0 \leq s < c$ in the clockwise order to get $\sigma^*(C_{uv})$. The isomorphism ψ in the former is denoted by $\psi = (1, s)$ and the latter by $\psi = (-1, s)$.

We distinguish the three cases: Case 1. Each of (u, u') and (v, v') is comparable in T; Case 2. Exactly one of (u, u') and (v, v') is comparable in T; and Case 3. Each of (u, u') and (v, v') is incomparable in T.

Case 1. Each of (u, u') and (v, v') is comparable in T: By $\mathrm{lca}(u, v) = \mathrm{lca}(u', v') = c_T$ and $\mathrm{dfs}(u) \leq \mathrm{dfs}(u')$, we see that u is an ancestor of u' and v' is an ancestor of v. In this case, the unique path $P(u', v)$ of T connecting u' and v contains vertices u', u, v' and v in this order. Denote the sequence of vertices in $P(u', v)$ by $u_0 (= u'), u_1, \ldots, u_h\ (= u), \ldots, u_{L-h}\ (= v'), \ldots, u_L\ (= v)$, where 1-augmented tree $T + uv$ (resp., $T = u'v'$) has a unique cycle $C_{uv} = (u_h, u_{h+1}, \ldots, u_L)$ (resp., $C_{u'v'} = (u_h, u_{h+1}, \ldots, u_{L-h}, u_0, u_1, \ldots, u_{h-1})$) and it holds $|V(C_{uv})| = |V(C_{u'v'})| = L - h + 1$. (See Figure S1.)

We first prove that $\sigma^*(u_h; C_{uv}) = \sigma^*(u_{L-h}; C_{u'v'})$ holds. Since $\sigma^*(u_h; C_{uv})$ is the signature of subtree G_{u_h} of 1-augmented tree $G = T + uv$, it cannot be equal to $\sigma^*(u_i; C_{u'v'})$ for any $i = 0, 1, \ldots, h$ (otherwise G_{u_h} would be a proper subtree of itself for $i < h$ or the degree of u_h in $T + uv$ would be equal to that of u_h in $T + u'v'$ for $i = h$). Hence $\psi(u_h) \in \{u_{h+1}, u_{h+2}, \ldots, u_{L-h}\}$. Note that $\sigma^*(x; C_{uv}) = \sigma^*(x; C_{u'v'})$ for all $x \in \{u_{h+1}, u_{h+2}, \ldots, u_{L-h-1}\} = V(C_{uv}) \cap V(C_{u'v'}) - \{u_h, u_{L-h}\}$. Hence $\psi(\psi(u_h)) \in \{u_{h+1}, u_{h+2}, \ldots, u_{L-h}\}$ and there is an integer $p \geq 1$ such that $\psi^p(u_h) = u_{L-h}$ since ψ gives a bijection between $V(C_{uv})$ and $V(C_{u'v'})$. This proves that

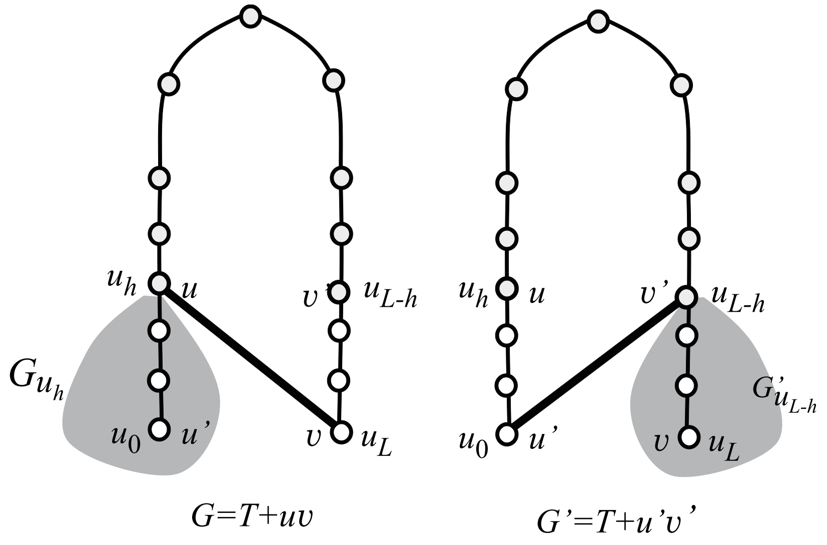

Figure S1: Case 1 (Each of (u, u') and (v, v') is comparable in T).

$\sigma^*(u_h; C_{uv}) = \sigma^*(u_{L-h}; C_{u'v'})$, i.e., the subtree G_{u_h} of 1-augmented tree $G = T + uv$ is rooted-isomorphic to the subtree $G'_{u_{L-h}}$ of 1-augmented tree $G' = T + u'v'$.

We further prove that $\sigma^*(u_{L-j}; C_{uv}) = \sigma^*(u_j; C_{u'v'})$ holds for all $j = 0, 1, \ldots, h-1$. To derive a contradiction, assume that there is an integer $k \leq h-1$ such that $\sigma^*(u_{L-k}; C_{uv}) \neq \sigma^*(u_k; C_{u'v'})$ and $\sigma^*(u_{L-j}; C_{uv}) = \sigma^*(u_j; C_{u'v'})$ holds for all $j = k+1, k+2, \ldots, h$. (See Figure S2.) Consider $\psi(u_{L-k})$. Note that $\psi(u_{L-k}) \neq u_{L-h}$, since $G_{u_{L-k}}$ of G is a proper subtree of $T_{u_{L-k}}$. If $\psi(u_{L-k}) = u_p \in \{u_{k+1}, u_{k+2}, \ldots, u_h\}$, then $\sigma^*(u_p; C_{u'v'}) = \sigma^*(u_{L-p}; C_{uv})$ holds by assumption on k and we regards that u_{L-k} is further mapped to $u_{L-p} \in V(C_{uv})$ to apply ψ to u_{L-p}. More formally we consider a mapping ξ such that $\xi(x) = \psi(x)$ for $x \in \{u_{h+1}, u_{h+2}, \ldots, u_{L-h-1}\}$ and $\xi(u_p) = \psi(u_{L-p})$ for $u_p \in \{u_{k+1}, u_{k+2}, \ldots, u_h\}$. Hence by repeatedly mapping with ξ as long as it is mapped to a vertex in $\{u_{k+1}, u_{k+2}, \ldots, u_h\} \cup \{u_{h+1}, u_{h+2}, \ldots, u_{L-h-1}\}$, we see that there is a vertex u_q with $0 \leq q < k$ such that $\sigma^*(u_{L-k}; C_{uv}) = \sigma^*(u_q; C_{u'v'})$ (recall that $\sigma^*(u_{L-k}; C_{uv}) \neq \sigma^*(u_k; C_{u'v'})$). This means that the subtree $G_{u_{L-k}}$ of 1-augmented tree $G = T + uv$ is adjacent to a proper descendant of u_k in T, which contradicts that $G_{u_h} \underset{r}{\approx} G'_{u_{L-h}}$ (since $\sigma^*(u_{L-j}; C_{uv}) = \sigma^*(u_j; C_{u'v'})$ holds for all $j = k, k+1, \ldots, h-1$). Therefore

$$\sigma^*(u_{L-j}; C_{uv}) = \sigma^*(u_j; C_{u'v'}) \text{ holds for all } j = 0, 1, \ldots, h. \tag{3}$$

We now distinguish two cases: (i) $\psi = (1, s)$; and (ii) $\psi = (-1, s)$.

(i) $\psi = (1, s)$: As we have observed that there is an integer $p \geq 1$ such that $\psi^p(u_h) = u_{L-h}$. Let $a_0 = u_h$, $a_i = \psi^i(u_h)$ for $i = 1, 2, \ldots, p$, where vertices $a_0 = u_h, a_1, \ldots, a_p = u_{L-h}$ belong to $V(C_{uv}) \cap V(C_{u'v'})$, but they may not appear along C_{uv} in this order. In particular, $\sigma^*(a_i; C_{uv}) = \sigma^*(a_i; C_{u'v'})$ holds for all $1 \leq i \leq p-1$. Let $\{u_{\ell_0}, u_{\ell_1}, \ldots, u_{\ell_p}\} = \{a_0, a_1, \ldots, a_p\}$, where $h = \ell_0 < \ell_1 < \cdots < \ell_p = L-h$. Let $S_1, S_2, \ldots, S_p$ be the sequences of vertices between two consecutive vertices u_{ℓ_i} and $u_{\ell_{i+1}}$; i.e., $S_i = (u_{\ell_{i-1}+1}, u_{\ell_{i-1}+2}, \ldots, u_{\ell_i-1})$ for each $i = 1, 2, \ldots, p$. Let $S'_1 = (u_0, u_1, \ldots, u_{\ell_1-1}, u_{\ell_1})$ and $S'_p = (u_{\ell_{p-1}}, u_{\ell_{p-1}+1}, \ldots, u_L)$. Let $\sigma^*(S_i)$ denote the sequence of codes $\sigma^*(x)$ of vertices x along S_i (note that $\sigma^*(x; C_{uv}) = \sigma^*(x; C_{u'v'})$ for each of such x). Let $\sigma^*(S'_1)$ denote the sequence of codes $\sigma^*(x; C_{u'v'})$ of vertices x along S'_1 and $\sigma^*(S'_p)$ the sequence of codes $\sigma^*(x; C_{uv})$ of vertices x along S'_p. (See Figure S3.)

We will show that (a) each of S_i, S'_1 and S'_p is symmetric in the sense that it is identical with its reverse sequence; and (b) $\sigma^*(S'_1) = \sigma^*(S'_p)$ and for each $S_i = (u_j, \ldots, u_{j'})$, there is a sequence $S_{i'}$ in its symmetric position (i.e., $S_{i'} = (u_{L-j'}, \ldots, u_{L-j})$). Note that conditions (a) and (b) indicate that T has two subtrees T_{a^*} and T_{b^*} satisfying condition (2), and hence $T + uv$ and $T + u'v'$ admit

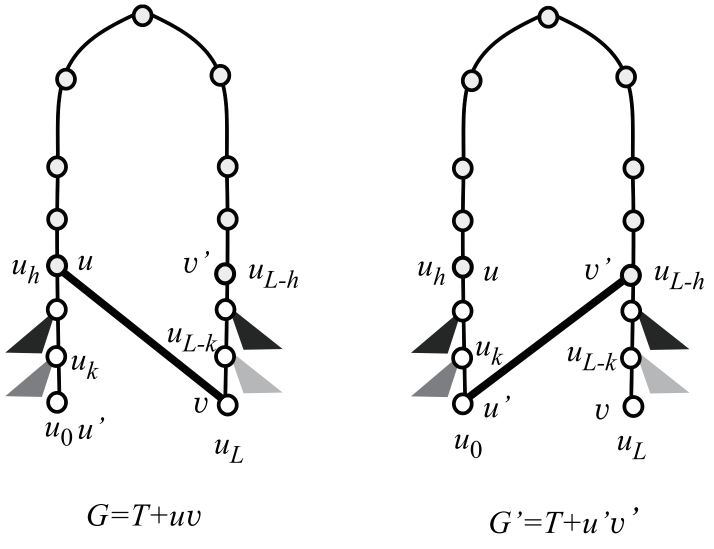

Figure S2: Case 1 (Each of (u, u') and (v, v') is comparable in T).

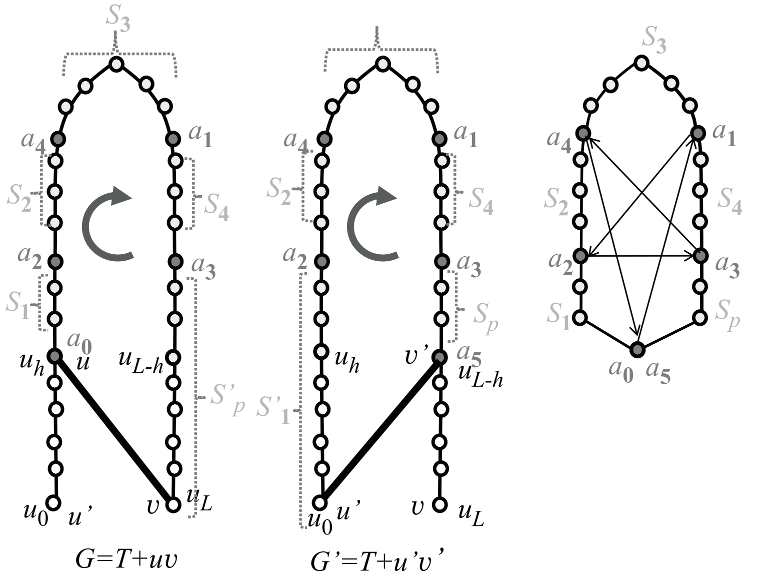

Figure S3: Case (i) $\psi = (1; s)$.

a rooted-isomorphism, which contradicts the assumption on (u, v) and (u', v').

Since $\psi = (1, s)$ maps a vertex u_k in C_{uv} to the vertex u_{k+s} (when $k+s \le L$) or u_{k+s-L} (when $k + s > L$), we see that each sequence S_i (or S'_p) in G is entirely mapped to another sequence S_j for some j (or S'_1) in G'. Let us trace this starting from sequence S'_p in C_{uv}, where we call S'_p a *long* sequence. By $\psi = (1, s)$, sequence S'_p is mapped to some sequence S_{i_1} in $C_{u'v'}$. Then sequence S_{i_1} in C_{uv} is mapped to some sequence S_{i_2} in $C_{u'v'}$. In this way, we have a series of sequences $S_{i_1}, S_{i_2}, \dots, S_{i_k}$ such that S_{i_j} is mapped to $S_{i_{j+1}}$ until $S_{i_k} = S'_1$ holds for some k. We call $S_{i_1}, S_{i_2}, \dots, S_{i_{k-1}}$ long sequences. Note that $S_{i_k} = S'_1$ is not in C_{uv} and S_1 is a subsequence of S'_1. Let us continue to trace how S_1 will be mapped by $\psi = (1, s)$. The sequence S_1 will be mapped to some sequence $S_{i_{k+1}}$ in $C_{u'v'}$. Similarly with long sequences $S_{i_1}, S_{i_2}, \dots, S_{i_{k-1}}$, we have a series of sequences $S_{i_{k+1}}, S_{i_{k+2}}, \dots, S_{i_p} = S_p$ such that S_{i_j} is mapped to $S_{i_{j+1}}$, where we call $S_{i_{k+1}}, S_{i_{k+2}}, \dots, S_{i_p} = S_p$ *short* sequences.

First we prove property (a). Let $g = \lfloor |S'_1|/h \rfloor$ and $d = |S'_1| - hg$, where $|S'_1| = hg + d$ and $d < h$. By (3) and $\sigma^*(S_{i_k}) = \sigma^*(S'_1)$, the subsequence $A = (\sigma^*(u_0; C_{u'v'}), \sigma^*(u_1; C_{u'v'}), \dots, \sigma^*(u_{h-1}; C_{u'v'}))$ of the first h elements of $\sigma^*(S'_1)$ is symmetric with the subsequence of the last h elements of $\sigma^*(S'_1)$. Note that $\sigma^*(S_1)$ is obtained from $\sigma^*(S'_1)$ by deleting the last h elements. Since $\sigma^*(S_1) = \sigma^*(S_p)$ is equal to the sequence obtained from $\sigma^*(S'_1)$ by deleting the first h elements, which implies that $\sigma^*(S'_1)$ is $g - 1$ repetitions of A, finishing with the sequence $B = (\sigma^*(u_0; C_{u'v'}), \sigma^*(u_1; C_{u'v'}), \dots, \sigma^*(u_{d-1}; C_{u'v'}))$ of the first d element of $\sigma^*(S'_1)$ followed by the reverse of sequence A (recall that the subsequence A of the first h elements of $\sigma^*(S'_1)$ is symmetric with the subsequence of the last h elements of $\sigma^*(S'_1)$). Then each of A and B is symmetric to itself, and $\sigma^*(S'_1)$ is represented by an alternating sequence $B, A, B, \dots, A, B$ of them. This proves that $\sigma^*(S)$ of a short sequence S is symmetric and $\sigma^*(S)$ of a long sequence S is symmetric.

Next we prove property (b). Since ψ is a rotational mapping with a fixed shift s, the positions of vertices in $U^* = \{u_{\ell_0}, u_{\ell_1}, \dots, u_{\ell_p}\} = \{a_0, a_1, \dots, a_p\}$ are symmetric; i.e., $u_{\ell_i} \in U^*$ if and only $u_{L-\ell_i} \in U^*$. We first flip the positions of all vertices u_i in $P(u', v)$, i.e., consider the mapping from u_i to u_{L-i}. Let S''_p denote the long sequence obtained from S'_p by flipping the positions of the vertices in S'_p. Then consider the reverse ψ^{-1} of mapping ψ, and trace how the sequence S''_p will be mapped to other sequences in the flipped positions. Let $S_{i'_1}, S_{i'_2}, \dots, S_{i'_{k-1}}$ be the resulting series of long sequences. We have another interpretation of the series; $S_{i'_1}, S_{i'_2}, \dots, S_{i'_{k-1}}$ is the reverse of series $S_{i_1}, S_{i_2}, \dots, S_{i_{k-1}}$ obtained in the above. Since the position of $S_{i'_1}, S_{i'_2}, \dots, S_{i'_{k-1}}$ is flipped, the positions of $S_{i_1}, S_{i_2}, \dots, S_{i_{k-1}}$ form a symmetric distribution over the path $P(u_h, u_{L-h})$ in T. The rest of short sequences also admits symmetric positions, proving property (b).

(ii) $\psi = (-1, s)$: We first prove that $\psi(u_h) = u_{L-h}$. As we have observed, $\psi(u_h) \notin \{u_0, u_1, \dots, u_h\}$. Let $\psi(u_h) = u_k$ with $h + 1 \le k \le L - h - 1$. (See Figure S4.) Recall that $\sigma^*(C_{u'v'})$ is obtained by flipping $\sigma^*(C_{uv})$ at $t_1 = \mathrm{lca}(u, u') = u_h$ and then rotating the flipped sequence by some amount $0 \le s < c = |V(C_{uv})|$ in the clockwise order. We see that $s = k - h$, since the position of u_h will not change by the flipping and will change to u_k by the rotation by s. Hence ψ maps the code $\sigma^*(u_k, C_{uv})$ of u_k (u_{h+s}) to that of a vertex in G' by first picking the position of its flipped vertex u_{L+1-s} and by shifting it by $s = k - h$; i.e., $\psi(u_k) = u_{(L+1-s)+s}$, which is vertex u_h in $C_{u'v'}$. Thus $\psi(\psi(u_h)) = u_h$ and $\sigma^*(u_h, C_{uv}) = \sigma^*(u_h, C_{u'v'})$. This, however, contradicts that the degree of u_h in G is larger than that of u_h in G'. Hence $\psi(u_h) = u_{L-h}$. Analogously we have $\psi(u_{L-h}) = u_h$. This means that $\sigma^*(C_{u'v'})$ and $\sigma^*(C_{uv})$ are symmetric with respect to the axis passing through the centroid c_T. Therefore T has two subtrees T_{a^*} and T_{b^*} satisfying condition 2, and hence $T + uv$ and $T + u'v'$ admit a rooted-isomorphism, which contradicts the assumption on (u, v) and (u', v').

Case 2. Exactly one of (u, u') and (v, v') is comparable in T: Assume that neither of u and u' is an ancestor of the other and v is an ancestor of v' (the other case can be treated symmetrically). Denote the sequence of vertices in path $P(u, v')$ in T by u_0 $(= u), u_1, \dots, u_h$ $(= \mathrm{lca}(u, u')), \dots, u_{L-\ell}$ $(= v), \dots, u_L$ $(= v')$, and that in $P(u', \mathrm{lca}(u, u'))$ by u'_0 $(= u'), u'_1, \dots, u'_{h'}$ $(= u_h)$, where $h - h' = \ell \ge 0$. (See Figure S5.) In this case, we prove that $\sigma^*(u_h; C_{uv}) = \sigma^*(u_h; C_{u'v'})$, which indicates that the two children $a = u_{h-1}$ and $a' = u'_{h'-1}$ of $\mathrm{lca}(u, u')$ satisfy the condition (1), a contradiction to the assumption on choice of (u, v) and (v, v').

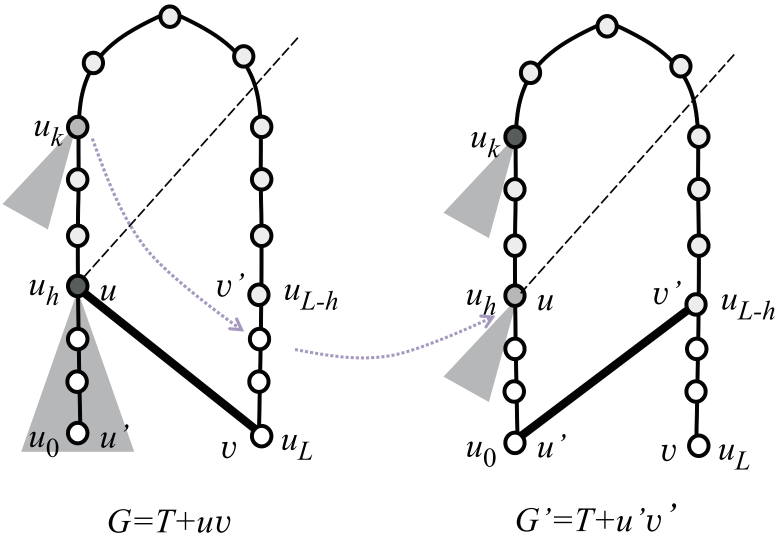

Figure S4: Case (ii) $\psi = (-1; s)$.

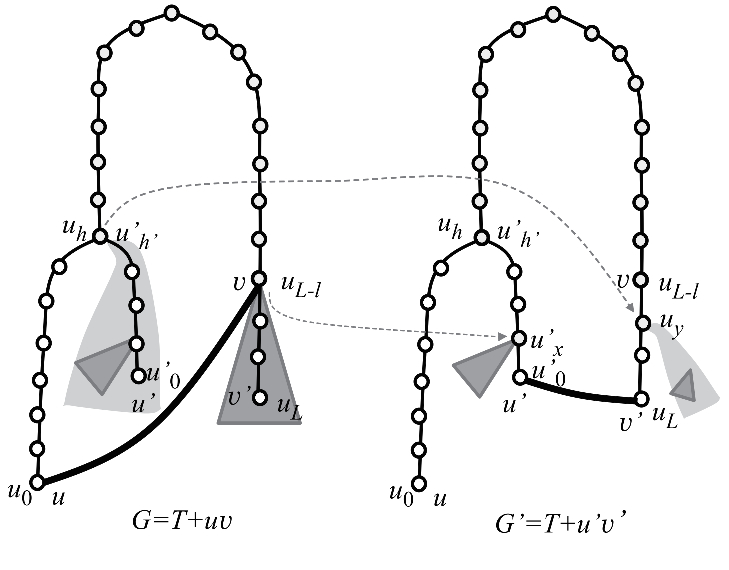

Figure S5: (a) $x < h'$ in Case 2 (Exactly one of (u, u') and (v, v') is comparable in T).

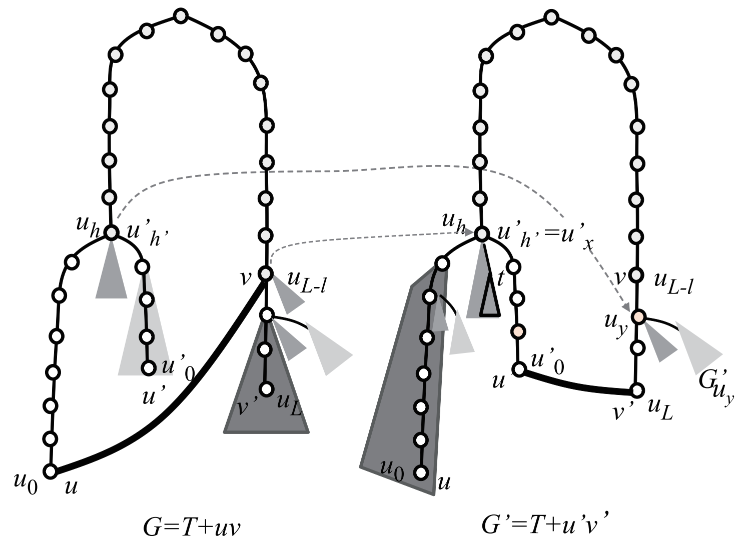

Figure S6: (b) $x = h'$ in Case 2 (Exactly one of (u, u') and (v, v') is comparable in T).

Note that $\psi(u_{L-\ell}) \notin \{u_{L-\ell}, u_{L-\ell+1}, \ldots, u_L\}$ holds by the same reason observed in Case 1. Since $\sigma^*(z; C_{uv}) = \sigma^*(z; C_{u'v'})$ for all $z \in \{u_{h+1}, u_{h+2}, \ldots, u_{L-\ell-1}\}$, there is an integer $p \geq 1$ such that $\psi^p(u_{L-\ell}) = u'_x \in \{u'_0, u'_1, \ldots, u'_{h'}\}$. Similarly, there are integers q and y such that $\psi^q(u_h) = u_y \in \{u_{L-\ell}, u_{L-\ell+1}, \ldots, u_L\}$. By the assumption of $\sigma^*(u_h; C_{uv}) \neq \sigma^*(u_h; C_{u'v'})$, we have $\psi^q(u_h) \neq u_h$. We distinguish two subcases.

(a) $x < h'$: In this case, the subtree $T_{u_{L-\ell}}$ of T is rooted-isomorphic to the subtree $G'_{u'_x}$ of $G' = T + u'v'$, and the subtree G_{u_h} of $G = T + uv$ properly contains a subtree rooted-isomorphic to $G'_{u'_x} \underset{r}{\approx} T_{u_{L-\ell}}$. (See Figure S5.) This means that $\psi^q(u_h) = u_y \in \{u_{L-\ell}, u_{L-\ell+1}, \ldots, u_L\}$ cannot hold, since otherwise G'_{u_y} of $G' = T + u'v'$ would properly contain a subtree rooted-isomorphic to $T_{u_{L-\ell}}$. Hence case (a) cannot occur.

(b) $x = h'$: In this case, the degree of u_h in G' (also in G) is equal to that degree of $u_{L-\ell}$ in $G = T + uv$, which is larger than that of $u_{L-\ell}$ in $G' = T + u'v'$. This means that $\sigma^*(u_h; C_{uv}) = \sigma^*(u_{L-\ell}; C_{u'v'})$ and $y \neq L - \ell$. (See Figure S6.) Now $G_{u_h} \underset{r}{\approx} G'_{u_y}$ and $G_{u_{L-\ell}} \underset{r}{\approx} G'_{u_h}$. Since $y \neq L - \ell$, $G_{u_{L-\ell}}$ (and hence G'_{u_h}) properly contains a subtree T' rooted-isomorphic to G'_{u_y} ($\underset{r}{\approx} G_{u_h}$). Moreover such a subtree T' cannot appear as a subtree of T_t of any other child t of u_h than $t = u_{h-1}$ in G'_{u_h} (otherwise T' would contain a subtree rooted-isomorphic to T_t). This means that two subtrees $T_{u_{h-1}}$ and $T_{u_{L-\ell+1}}$ of T are rooted-isomorphic.

We further prove that

$$\text{for each } i = 1, 2, \ldots, \ell, \text{ it holds } \sigma^*(u_{h-i}; C_{uv}) = \sigma^*(u_{L-\ell+i}; C_{u'v'}), \text{ i.e., } G_{u_{h-i}} \underset{r}{\approx} G'_{u_{L-\ell+i}}. \quad (4)$$

Assume that there is an integer $1 \leq k \leq \ell$ such that $\sigma^*(u_{h-i}; C_{uv}) = \sigma^*(u_{L-\ell+i}; C_{u'v'})$ for all $i = 1, 2, \ldots, k-1$ and $\sigma^*(u_{h-k}; C_{uv}) \neq \sigma^*(u_{L-\ell+k}; C_{u'v'})$. Since $G_{u_{h-i}} \underset{r}{\approx} G'_{u_{L-\ell+i}}$ $(1 \leq i < k)$, we see from $G_{u_{h-k}} \underset{r}{\not\approx} G'_{u_{L-\ell+k}}$ that u_{h-k} has a child w such that the subtree T_w in T is rooted-isomorphic to the subtree $T_{u_{L-\ell+k+1}}$ in T. (See Figure S7.) This means that $\sigma^*(u_{h-k}; C_{uv}) \neq \sigma^*(z; C_{u'v'})$ for all $z \in \{u_{L-\ell+k+1}, u_{L-\ell+k+2}, \ldots, u_L\}$. Let us trace how u_{h-k} in $G = T + uv$ will be mapped to vertices in $G' = T + u'v'$ by ψ. Also $\sigma^*(u_{h-k}; C_{uv}) \neq \sigma^*(u_{L-\ell}; C_{u'v'})$ since the subtree $G_{u_{h-k}}$ of $G = T + uv$ is a proper subtree of $T_{u_{L-\ell}}$. As we have observed, if $\psi(u_{h-k}) = t \in \{u_{h+1}, u_{h+2}, \ldots u_{L-\ell-1}\}$, where $\sigma^*(t; C_{uv}) = \sigma^*(t; C_{u'v'})$ for all $t \in \{u_{h+1}, u_{h+2}, \ldots u_{L-\ell-1}\}$, then we continue to consider $\psi(t)$ of $t \in V(C_{uv})$. Similarly, if $\psi(u_{h-k}) = u_{L-\ell+i} \in \{u_{L-\ell+1}, u_{L-\ell+2}, \ldots u_{L-\ell+k-1}\}$, where $\sigma^*(u_{h-i}; C_{uv}) = \sigma^*(u_{L-\ell+i}; C_{u'v'})$

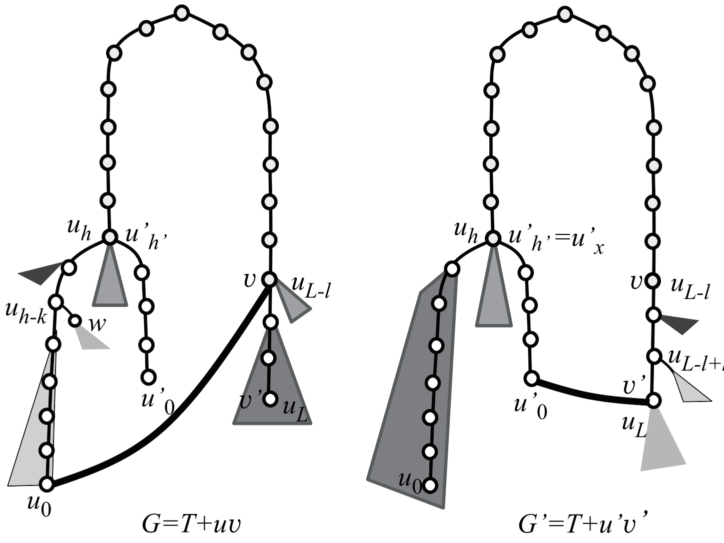

Figure S7: Case 2 (Exactly one of (u, u') and (v, v') is comparable in T).

$(1 \leq i < k)$, then we continue to consider $\psi(u_{h-i})$ of $u_{h-i} \in V(C_{uv})$. Since ψ is a bijection, by repeating this we see that u_{h-k} will be mapped to a vertex $z \in \{u_{L-\ell+k+1}, u_{L-\ell+k+2}, \ldots, u_L\}$, which contradicts that $\sigma^*(u_{h-k}; C_{uv}) \neq \sigma^*(z; C_{u'v'})$ for all $z \in \{u_{L-\ell+k+1}, u_{L-\ell+k+2}, \ldots, u_L\}$. This proves (4).

Now let us trace how u_h in $G = T + uv$ will be mapped to vertices in $G' = T + u'v'$. Note that $\sigma^*(u_h; C_{uv}) \neq \sigma^*(u_{L-\ell}; C_{u'v'})$, as already observed. Also $\sigma^*(u_h; C_{uv}) \neq \sigma^*(u'_i; C_{u'v'})$ for all $i = 0, 1, \ldots, h'$ since G_{u_h} cannot be rooted-isomorphic to its proper subtree (recall that $\sigma^*(u_h; C_{uv}) \neq \sigma^*(u'_{h'}; C_{u'v'})$ is already assumed in Case 2). As we have observed, if $\psi(u_h) = t \in \{u_{h+1}, u_{h+2}, \ldots u_{L-\ell-1}\}$, where $\sigma^*(t; C_{uv}) = \sigma^*(t; C_{u'v'})$ for all $t \in \{u_{h+1}, u_{h+2}, \ldots u_{L-\ell-1}\}$, then we continue to consider $\psi(t)$ of $t \in V(C_{uv})$. Similarly, if $\psi(u_h) = u_{L-\ell+i} \in \{u_{L-\ell+1}, u_{L-\ell+2}, \ldots u_L\}$, where $\sigma^*(u_{h-i}; C_{uv}) = \sigma^*(u_{L-\ell+i}; C_{u'v'})$ $(1 \leq i \leq \ell)$ by (4), then we continue to consider $\psi(u_{h-i})$ of $u_{h-i} \in V(C_{uv})$. Since ψ is a bijection, this can be repeated infinitely many time, contradicting the finiteness of graphs. This proves that (b) cannot occur either.

Case 3. Each of (u, u') and (v, v') is incomparable in T: Denote the sequence of vertices in the path $P(u, v)$ by u_0 $(= u), u_1, \ldots, u_h$ $(= \text{lca}(u, u')), \ldots, u_{L-\ell}$ $(= \text{lca}(v, v')), \ldots, u_L$ $(= v)$, and those in $P(u', v')$ by u'_0 $(= u'), u'_1, \ldots, u'_{h'}$ $(= \text{lca}(u, u')), \ldots, u'_{L-\ell'}$ $(= \text{lca}(v, v')), \ldots, u'_L$ $(= v')$, where $h + \ell = h' + \ell'$. (See Figure S8.)

We first claim that

$$\sigma^*(u_h; C_{uv}) \neq \sigma^*(t'; C_{u'v'}) \text{ for all } t' \in \{u'_0, u'_1, \ldots, u'_{h'}\} \cup \{u'_{L-\ell'+1}, u'_{L'-\ell'+2}, \ldots, u'_L\}. \quad (5)$$

By assumption on (u, v) and (u', v'), we know that $\sigma^*(u_h; C_{uv}) \neq \sigma^*(u'_{h'}; C_{u'v'})$, where $u_h = u'_{h'} = \text{lca}(u, u')$ (otherwise two children $a = u_{h-1}$ and $a' = u'_{h'-1}$ in T of $\text{lca}(u, u')$ would satisfy the condition (1)). Clearly $\sigma^*(u_h; C_{uv}) \neq \sigma^*(t; C_{u'v'})$ for all $t' \in \{u'_0, u'_1, \ldots, u'_{h'-1}\}$, since otherwise the subtree G_{u_h} of $G = T + uv$ would be a proper subtree of itself.

Hence to prove (5) by deriving a contradiction, it suffices to assume that $\sigma^*(u_h; C_{uv}) = \sigma^*(z; C_{u'v'})$ for some $z \in \{u'_{L-\ell'+1}, u'_{L-\ell'+2}, \ldots, u'_L\}$. Hence the subtree G'_z of $G' = T + u'v'$ is rooted-isomorphic to the subtree G_{u_h} of $G = T + uv$. Let us trace how $u_{L-\ell} = \text{lca}(v, v')$ is mapped to vertices in $C_{u'v'}$. As we have observed, if $\psi(u_{L-\ell}) = t \in \{u_{h+1}, u_{h+2}, \ldots u_{L-\ell-1}\}$, where $\sigma^*(t; C_{uv}) = \sigma^*(t; C_{u'v'})$ for all $t \in \{u_{h+1}, u_{h+2}, \ldots u_{L-\ell-1}\}$, then we continue to consider $\psi(t)$ of $t := \psi(t)$. Hence $u_{L-\ell}$ will be mapped to a vertex $t^* \in \{u'_0, u'_1, \ldots, u'_{h'}\} \cup \{u'_{L-\ell'}, u'_{L-\ell'+1}, \ldots, u'_L\}$, where $\sigma^*(u_{L-\ell}; C_{uv}) = \sigma^*(t^*; C_{u'v'})$ holds. Since $G_{u_{L-\ell}}$ properly contains a subtree rooted-isomorphic to G_{u_h}, the vertex t^* cannot be in $\{u'_0, u'_1, \ldots, u'_{h'-1}\}$. Similarly if $t^* \in \{u'_{L-\ell'+1}, u'_{L-\ell'+2},$

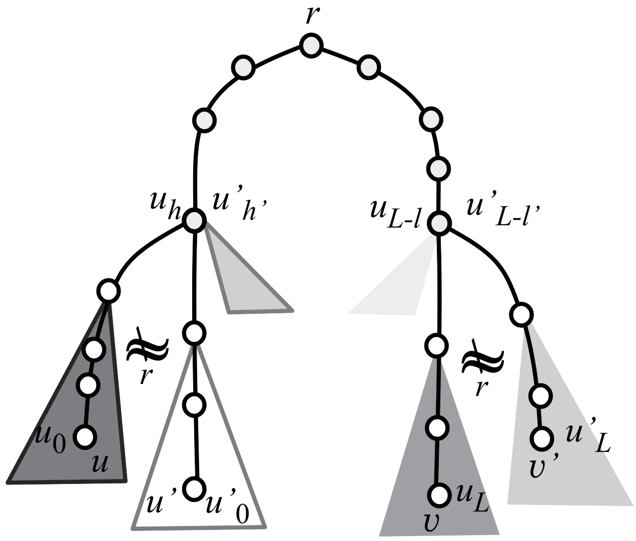

Figure S8: Case 3 (Each of (u, u') and (v, v') is incomparable in T).

$\ldots, u'_L\}$, then the subtree G'_{t^*} of $G' = T + u'v'$ would be a proper subtree of itself $G_{u_{L-\ell}} \underset{r}{\approx} G'_{t^*}$, a contradiction. Therefore there are only two cases: (a) $t^* = \mathrm{lca}(v, v') = u_{L-\ell} = u'_{L-\ell'}$; and (b) $t^* = \mathrm{lca}(u, u') = u_h = u'_{h'}$.

(a) $t^* = \mathrm{lca}(v, v') = u_{L-\ell} = u'_{L-\ell'}$: Let us trace how vertex $u'_{L-\ell'}$ $(= \mathrm{lca}(v, v') = u_{L-\ell})$ in $C_{u'v'}$ will be mapped to vertices in C_{uv} by ψ^{-1}. By assumption on (u, v) and (u', v'), we know that $\sigma^*(u_{L-\ell}; C_{uv}) \neq \sigma^*(u_{L-\ell}; C_{u'v'})$ (otherwise two children $a = u_{L-\ell+1}$ and $a' = u'_{L-\ell'+1}$ in T of $\mathrm{lca}(v, v')$ would satisfy the condition (1)). Also $\sigma^*(t; C_{uv}) \neq \sigma^*(u'_{L-\ell'}; C_{u'v'})$ for all $t \in \{u_{L-\ell+1}, u_{L-\ell+2}, \ldots, u_L\}$, since otherwise $G'_{u_{L-\ell}}$ would be a proper subtree of itself. We see that $\sigma^*(u_h; C_{uv}) \neq \sigma^*(u'_{L-\ell'}; C_{u'v'})$ since $G'_{u_{L-\ell}}$ properly contains a subtree rooted-isomorphic to G_{u_h}. Hence there is a vertex u_x with $0 \leq x \leq h-1$ such that $\sigma^*(u_x; C_{uv}) = \sigma^*(u'_{L-\ell'}; C_{u'v'})$.

Next consider how vertex $u'_{h'}$ $(= \mathrm{lca}(u, u') = u_h)$ in $C_{u'v'}$ will be mapped to vertices in C_{uv} by ψ^{-1}. Again by assumption on (u, v) and (u', v'), we know that $\sigma^*(u'_{h'}; C_{uv}) \neq \sigma^*(u'_{h'}; C_{u'v'})$. Also $\sigma^*(t; C_{uv}) \neq \sigma^*(u'_{h'}; C_{u'v'})$ for all $t \in \{u_0, u_1, \ldots, u_{h-1}\}$, since otherwise $G'_{u'_{h'}}$ would be a proper subtree of itself. We see that $\sigma^*(u_h; C_{uv}) \neq \sigma^*(u'_{h'}; C_{u'v'})$ since $u_x \in V(G'_{u'_{h'}})$ means that $G'_{u'_{h'}}$ properly contains a subtree rooted-isomorphic to G_{u_h}. Hence the remaining possibility is that $\sigma^*(u_{L-\ell}; C_{uv}) = \sigma^*(u'_{h'}; C_{u'v'})$. However, this is also impossible because $G'_{u'_{h'}}$ properly contains a subtree rooted-isomorphic to $G_{u_{L-\ell}} \underset{r}{\approx} G'_{u_{L-\ell}}$. Therefore case (a) cannot occur.

(b) $t^* = \mathrm{lca}(u, u') = u_h = u'_{h'}$: Now $G_{u_{L-\ell}} \underset{r}{\approx} G'_{u_h}$, which properly contains a subtree rooted-isomorphic to $G'_z \underset{r}{\approx} G_{u_h}$ by assumption. Let us trace how vertex $u'_{h'}$ $(= \mathrm{lca}(u, u') = u_h)$ in $C_{u'v'}$ will be mapped to vertices in C_{uv} by ψ^{-1}. Now $\sigma^*(u_h; C_{uv}) \neq \sigma^*(u'_{h'}; C_{u'v'})$. Also $\sigma^*(u_i; C_{uv}) \neq \sigma^*(u'_{h'}; C_{u'v'})$ for all $i = 0, 1, \ldots, h-1$ since otherwise $G_{u_{L-\ell}}$ would be a proper subtree of itself. By assumption on (u, v) and (u', v'), $\sigma^*(u_h; C_{uv}) \neq \sigma^*(u'_{h'}; C_{u'v'})$. Hence the remaining possibility is that there is a vertex $u_y \in \{u_{L-\ell+1}, u_{L-\ell+2}, \ldots, u_L\}$ such that $\sigma^*(u_y; C_{uv}) = \sigma^*(u'_{h'}; C_{u'v'})$.

Now consider how vertex $u'_{L-\ell'}$ $(= \mathrm{lca}(v, v') = u_{L-\ell})$ in $C_{u'v'}$ will be mapped to vertices in C_{uv} by ψ^{-1}. Since ψ is a bijection, there is a vertex $w \in \{u_0, u_1, \ldots, u_h\} \cup \{u_{L-\ell}, u_{L-\ell+1}, \ldots, u_L\}$ such that $\sigma^*(w; C_{uv}) = \sigma^*(u'_{L-\ell'}; C_{u'v'})$. Note that the subtree $G'_{u'_{L-\ell'}}$ has the vertex u_y and properly contains a subtree rooted-isomorphic to $G'_{u'_{h'}}$. Clearly $\sigma^*(u_i; C_{uv}) \neq \sigma^*(u'_{L-\ell'}; C_{u'v'})$ for all $i = L-\ell+1, L-\ell+2, \ldots, L$ since otherwise $G'_{u'_{L-\ell'}}$ would be a proper subtree of itself. Hence $w \in \{u_0, u_1, \ldots, u_h\} \cup \{u_{L-\ell}\}$. By assumption on (u, v) and (u', v'), $\sigma^*(u_{L-\ell}; C_{uv}) \neq \sigma^*(u'_{L-\ell'}; C_{u'v'})$.

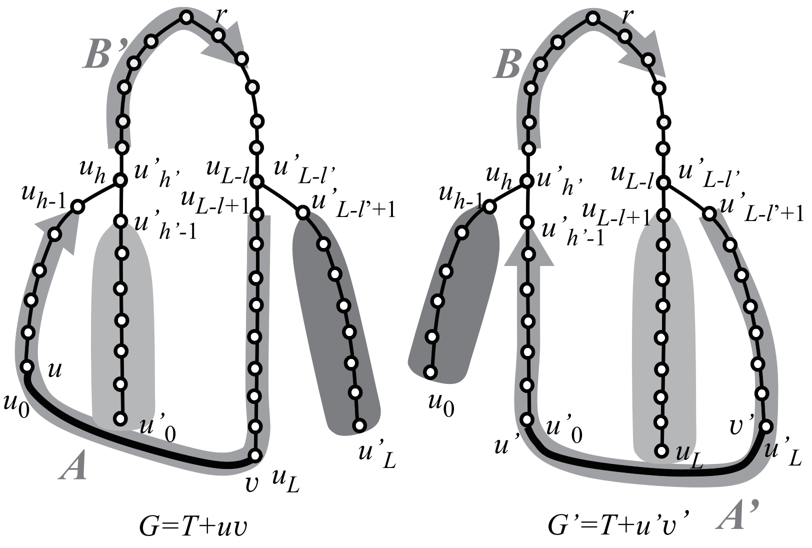

Figure S9: Sequences A, A', B and B'.

Also $\sigma^*(u_i; C_{uv}) \neq \sigma^*(u'_{L-\ell'}; C_{u'v'})$ for all $i = 0, 1, \ldots, h-1$ since $G'_{u'_{L-\ell'}}$ properly contains a subtree rooted-isomorphic to G'_{u_h}. Finally $\sigma^*(u_h; C_{uv}) \neq \sigma^*(u'_{L-\ell'}; C_{u'v'})$ since $G'_{u'_{L-\ell'}}$ properly contains a subtree rooted-isomorphic to G_{u_h}. Therefore such a vertex w cannot exist and (b) does not occur either, proving (5).

Symmetrically we also obtain $\sigma^*(t'; C_{u'v'}) \neq \sigma^*(u_{L-\ell}; C_{uv})$ for all $t' \in \{u'_0, u'_1, \ldots, u'_{h'}\} \cup \{u'_{L-\ell'+1}, u'_{L'-\ell'+2}, \ldots, u'_L\}$, and $\sigma^*(u_h; C_{u'v'}) \neq \sigma^*(t; C_{uv}) \neq \sigma^*(u_{L-\ell}; C_{u'v'})$ for all $t \in \{u_0, u_1, \ldots, u_h\} \cup \{u_{L-\ell+1}, u_{L-\ell+2}, \ldots, u_L\}$.

Then (5) implies $\sigma^*(u_h; C_{uv}) = \sigma^*(u_{L-\ell}; C_{u'v'})$, by repeatedly taking $t' := \psi(t')$ as long as $t' \in \{u_{h+1}, u_{h+2}, \ldots u_{L-\ell-1}\}$, where $\sigma^*(t'; C_{uv}) = \sigma^*(t'; C_{u'v'})$ for all $t' \in \{u_{h+1}, u_{h+2}, \ldots u_{L-\ell-1}\}$. Analogously we also have $\sigma^*(u_{L-\ell}; C_{uv}) = \sigma^*(u_h; C_{u'v'})$. Hence we have the following properties.

$$\begin{aligned}
&\sigma^*(u_h; C_{uv}) = \sigma^*(u_{L-\ell}; C_{u'v'}),\ \sigma^*(u_{L-\ell}; C_{uv}) = \sigma^*(u_h; C_{u'v'}),\\
&\sigma^*(u_h; C_{uv}) \neq \sigma^*(t'; C_{u'v'}) \neq \sigma^*(u_{L-\ell}; C_{uv})\\
&\text{for all } t' \in \{u'_0, u'_1, \ldots, u'_{h'}\} \cup \{u'_{L-\ell'+1}, u'_{L'-\ell'+2}, \ldots, u'_L\}, \text{ and}\\
&\sigma^*(u_h; C_{u'v'}) \neq \sigma^*(t; C_{uv}) \neq \sigma^*(u_{L-\ell}; C_{u'v'})\\
&\text{for all } t \in \{u_0, u_1, \ldots, u_h\} \cup \{u_{L-\ell+1}, u_{L-\ell+2}, \ldots, u_L\}.
\end{aligned} \tag{6}$$

In (6), $\sigma^*(u_h; C_{uv}) = \sigma^*(u_{L-\ell}; C_{u'v'})$ and $\sigma^*(u_{L-\ell}; C_{uv}) = \sigma^*(u_h; C_{u'v'})$ mean that $G_{u_h} \underset{r}{\approx} G'_{u_{L-\ell}}$ and $G_{u_{L-\ell}} \underset{r}{\approx} G'_{u_h}$. Note that G_{u_h} (resp., G'_{u_h}) is obtained from T_{u_h} by removing the subtree $T_{u_{h-1}}$ (resp., $T_{u'_{h'-1}}$), while $G_{u_{L-\ell}}$ (resp., $G'_{u_{L-\ell}}$) is obtained from $T_{u_{L-\ell}}$ by removing the subtree $T_{u_{L-\ell+1}}$ (resp., $T_{u'_{L-\ell'+1}}$). (See Figure S9.) Since $T_{u_{h-1}} \underset{r}{\not\approx} T_{u'_{h'-1}}$ and $T_{u_{L-\ell+1}} \underset{r}{\not\approx} T_{u'_{L-\ell'+1}}$ by assumption, we see that

$$T_{u'_{h'-1}} \underset{r}{\approx} T_{u_{L-\ell+1}} \text{ and } T_{u_{h-1}} \underset{r}{\approx} T_{u'_{L-\ell'+1}}. \tag{7}$$

We distinguish two subcases: (i) $\psi = (-1, s)$; and (ii) $\psi = (1, s)$.

(i) $\psi = (-1; s)$: In this case, we can prove that $\psi(\mathrm{lca}(u, u')) = \mathrm{lca}(v, v')$ and $\psi(\mathrm{lca}(v, v')) = \mathrm{lca}(u, u')$ in the same manner of Case 1. This means that $\sigma^*(C_{u'v'})$ and $\sigma^*(C_{uv})$ are symmetric with respect to the axis passing through the centroid c_T. Therefore T has two subtrees T_{a^*} and T_{b^*} satisfying condition (2), and hence $T + uv$ and $T + u'v'$ admit a rooted-isomorphism, which contradicts the assumption on (u, v) and (u', v').

(ii) $\psi = (1, s)$: In this case, the cyclic sequence $\sigma^*(C_{uv})$ will match with $\sigma^*(C_{u'v'})$ after taking $\mathrm{lca}(u, u')$ as the common start position and rotating $\sigma^*(C_{uv})$ by s in the clockwise order. By $|V(C_{uv})| = |V(C_{u'v'})|$, we have $h + \ell = h' + \ell'$ and $L - h - \ell = L - h' - \ell'$. By (6), vertex $\mathrm{lca}(u, u') = u_h = u'_{h'}$ (resp., $\mathrm{lca}(v, v') = u_{L-\ell} = u'_{L-\ell'}$) in C_{uv} will never be mapped to any vertex u'_i with $i \in \{0, 1, \ldots, h'\} \cup \{L - \ell' + 1, L - \ell' + 2, \ldots, L\}$ after repeating the rotation by any number of times. This means that $s \le L - h - \ell = L - h' - \ell'$ (resp., $h + \ell = h' + \ell' < s$).

Let A be the subsequence of $\sigma^*(C_{uv})$ from $u_{L-\ell+1}$ to u_{h-1}; i.e.,

$$A = (\sigma^*(u_{L-\ell+1}; C_{uv}), \sigma^*(u_{L-\ell+2}; C_{uv}), \ldots, \sigma^*(u_L; C_{uv}), \sigma^*(u_0; C_{uv}), \sigma^*(u_1; C_{uv}), \ldots, \sigma^*(u_{h-1}; C_{uv})),$$

where the length $|A|$ of A is $h + \ell - 2$ $(< s)$. Let B be the subsequence of $\sigma^*(C_{u'v'})$ from u_{h+1} to $u_{2h+\ell-1}$; i.e.,

$$B = (\sigma^*(u_{h+1}; C_{u'v'}), \sigma^*(u_{h+2}; C_{u'v'}), \ldots, \sigma^*(u_{2h+\ell-1}; C_{u'v'})),$$

where $|A| = |B|$. (See Figure S9.) Since vertex $u_{L-\ell}$ will match with u_h by repeating the rotation of $\sigma^*(C_{uv})$ by s some number of times, we see that A matches with B after repeating the rotation by some number of times.

Let B' be the subsequence of $\sigma^*(C_{uv})$ from u_{h+1} to $u_{2h+\ell-1}$; i.e.,

$$B' = (\sigma^*(u_{h+1}; C_{uv}), \sigma^*(u_{h+2}; C_{uv}), \ldots, \sigma^*(u_{2h+\ell-1}; C_{uv})),$$

where B is equal to B' since $\{u_{h+1}, u_{h+2}, \ldots, u_{2h+\ell-1}\} \subseteq V(C_{uv}) \cap V(C_{u'v'}) - \{u_h, u_{L-\ell}\}$. Let A' be the subsequence of $\sigma^*(C_{u'v'})$ from $u'_{L-\ell'+1}$ to $u'_{h'-1}$; i.e.,

$$\begin{aligned} A' \;=\; & (\sigma^*(u'_{L-\ell'+1}; C_{u'v'}), \sigma^*(u_{L-\ell'+2}; C_{u'v'}), \ldots, \\ & \sigma^*(u'_L; C_{u'v'}), \sigma^*(u'_0; C_{u'v'}), \sigma^*(u'_1; C_{u'v'}), \ldots, \sigma^*(u'_{h'-1}; C_{u'v'})), \end{aligned}$$

Similarly A' matches with B' $(= B)$. Therefore we have $A = A'$.

Note that $h \ne h'$ since otherwise $A = A'$ would imply that $a = u_{h-1}$ and $a' = u'_{h'-1}$ in T satisfy (1). Assume $h < h'$ (the other case of $h > h'$ can be treated analogously). Since $A = A'$, we know that

$$\sigma^*(u_{h-i}; C_{uv}) = \sigma^*(u'_{h'-i}; C_{u'v'}) \text{ for } i = 1, 2, \ldots, h \ (< h'). \tag{8}$$

Symmetrically we also see that

$$\sigma^*(u_{L-\ell+i}; C_{uv}) = \sigma^*(u'_{L-\ell'+i}; C_{u'v'}) \text{ for } i = 1, 2, \ldots, \ell' \ (< \ell). \tag{9}$$

Recall that $T_{u'_{h'-1}} \underset{r}{\approx} T_{u_{L-\ell+1}}$ and $T_{u_{h-1}} \underset{r}{\approx} T_{u'_{L-\ell'+1}}$ by (7). From this, (8) and (9), we here show that

$$\sigma^*(u_{h-i}; C_{uv}) = \sigma^*(u'_{h'-i}; C_{u'v'}) = \sigma^*(u_{L-\ell+i}; C_{uv}) = \sigma^*(u'_{L-\ell'+i}; C_{u'v'}) \text{ for } i = 1, 2, \ldots, \min\{h, \ell'\}. \tag{10}$$

To prove this, we indirectly assume that there is an index j $(\le \min\{h, \ell'\})$ such that $\sigma^*(u_{h-j}; C_{uv}) \ne \sigma^*(u'_{L-\ell'+j}; C_{u'v'})$ (i.e., $G_{u_{h-j}} \underset{r}{\not\approx} G'_{u'_{L-\ell'+j}}$) and $\sigma^*(u_{h-i}; C_{uv}) = \sigma^*(u'_{L-\ell'+i}; C_{u'v'})$ for all $i = 1, 2, \ldots, j - 1$, where the latter indicates that

$$T_{u_{h-j}} \underset{r}{\approx} T_{u'_{L-\ell'+j}} \text{ and } T_{u'_{h'-j}} \underset{r}{\approx} T_{u_{L-\ell+j}}$$

since (7) holds. (See Figure S10.) This and $G_{u_{h-j}} \underset{r}{\not\approx} G'_{u'_{L-\ell'+j}}$ mean that $T_{u_{h-j-1}} \underset{r}{\not\approx} T_{u'_{L-\ell'+j+1}}$. Let k be the number of subtrees T_t rooted at a child t of $u'_{L-\ell'+j}$ such that $T_t \underset{r}{\approx} T_{u'_{L-\ell'+j+1}}$, where $k \ge 1$ (possibly $t = u'_{L-\ell'+j+1}$). Since $T_{u_{h-j}} \underset{r}{\approx} T_{u'_{L-\ell'+j}}$ but $T_{u_{h-j-1}} \underset{r}{\not\approx} T_{u'_{L-\ell'+j+1}}$, we see that the number of subtrees T_x rooted at a child x $(\ne u_{h-j-1})$ of u_{h-j} such that $T_x \underset{r}{\approx} T_{u'_{L-\ell'+j+1}}$ is also k. Since $\sigma^*(u_{h-j}; C_{uv}) = \sigma^*(u'_{h'-j}; C_{u'v'})$, we see that the number of subtrees T_y rooted at a child y $(\ne u'_{h'-j-1})$ of $u'_{h'-j}$ such that $T_y \underset{r}{\approx} T_{u'_{L-\ell'+j+1}}$ is also k. By $T_{u'_{h'-j}} \underset{r}{\approx} T_{u_{L-\ell+j}}$, we see that

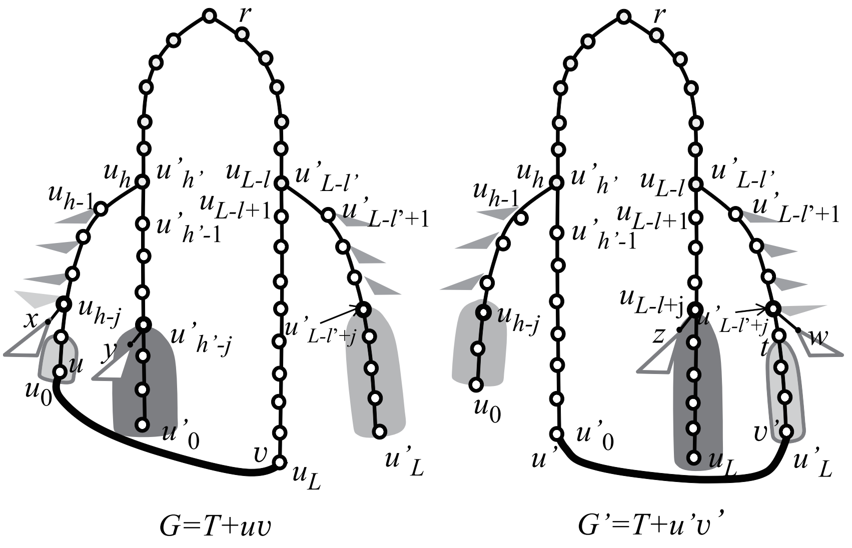

Figure S10: $T + uv$ and $T + u'v'$ satisfying equation (10).

the number of subtrees T_z rooted at a child z ($\neq u_{L-\ell+j+1}$) of $u_{L-\ell+j}$ such that $T_z \underset{r}{\approx} T_{u'_{L-\ell'+j+1}}$ is also k (note that $T_{u_{L-\ell+j+1}} \underset{r}{\not\approx} T_{u'_{L-\ell'+j+1}}$). Since $\sigma^*(u_{L-\ell+j}; C_{uv}) = \sigma^*(u'_{L-\ell'+j}; C_{u'v'})$, this means that the number of subtrees T_w rooted at a child w ($\neq u'_{L-\ell'+j+1}$) of $u'_{L-\ell'+j}$ such that $T_w \underset{r}{\approx} T_{u'_{L-\ell'+j+1}}$ is also k. This contradicts that the number of such subtrees must be $k-1$, since $T_{u'_{L-\ell'+j+1}}$ is counted in the original k. This proves (10). This also implies that $h = \ell'$, $h' = \ell$ and $T_{u'_{h'-h-1}} \underset{r}{\approx} T_{u_{L-h'+h+1}}$ (since $h \neq \ell'$ means $T_{u_{h-j-1}} \underset{r}{\not\approx} T_{u'_{L-\ell'+j+1}}$ for $j = \min\{h, \ell'\}$, which leads to a contradiction in the same manner).

We next prove that subsequence

$$C = (\sigma^*(u_{L-h'+h+1}; C_{uv}), \sigma^*(u_{L-h'+h+2}; C_{uv}), \ldots, \sigma^*(u_L; C_{uv}))$$

of A is symmetric (i.e., it matches with its reverse sequence). Note that C is equal to subsequence

$$C' = (\sigma^*(u'_0; C_{u'v'}), \sigma^*(u'_1; C_{u'v'}), \ldots, \sigma^*(u'_{h'-h-1}; C_{u'v'}))$$

of A', since each of C and C' corresponds to the same subsequence of $A = A'$. (See Figure S11.) Note that C passes through tree $T_{u_{L-h'+h+1}}$ ($\underset{r}{\approx} T_{u'_{h'-h-1}}$) from $u_{L-h'+h+1}$ to u_L, while C' ($= C$) passes through tree $T_{u'_{h'-h-1}}$ ($\underset{r}{\approx} T_{u_{L-h'+h+1}}$) from u'_0 to $u'_{h'-h-1}$. If $\sigma^*(u_{L-h+h'+1}; C_{uv}) \neq \sigma^*(u_L; C_{uv})$ ($= \sigma^*(u'_{h'-h-1}; C_{u'v'})$) then $T_{u_{L-h+h'+2}} \underset{r}{\not\approx} T_{u'_{h'-h-2}}$ and $u'_{h'-h-1}$ has some other child t ($\neq u'_{h'-h-2}$) such that $T_t \underset{r}{\approx} T_{u_{L-h+h'+2}}$. In this case, the code $\sigma^*(u_L; C_{uv})$ ($= \sigma^*(u'_{h'-h-1}; C_{u'v'})$) of vertex u_L means that there is a child x of u_L such that $T_x \underset{r}{\approx} T_{u_{L-h+h'+2}}$, which is a contradiction (since T_x is now a proper subtree of $T_{u_{L-h+h'+2}}$). Hence $\sigma^*(u_{L-h+h'+1}; C_{uv}) = \sigma^*(u_L; C_{uv})$. More generally assume that there is an index k ($\leq \frac{h'-h}{2}$) such that $\sigma^*(u_{L-h+h'+1+k}; C_{uv}) \neq \sigma^*(u_{L-k}; C_{uv})$ and $\sigma^*(u_{L-h+h'+1+i}; C_{uv}) = \sigma^*(u_{L-i}; C_{uv})$ for all $i = 0, 1, \ldots, k-1$. By the second condition, we have $T_{u_{L-h+h'+k}} \underset{r}{\approx} T_{u'_{h'-h-k}}$. By the first condition, $T_{u_{L-h+h'+k+1}} \underset{r}{\not\approx} T_{u'_{h'-h-k-1}}$ holds and vertex $u'_{h'-h-k}$ has some other child t ($\neq u'_{h'-h-k-1}$) such that $T_t \underset{r}{\approx} T_{u_{L-h+h'+k+1}}$. In this case, the code $\sigma^*(u_{L-k}; C_{uv})$ ($= \sigma^*(u'_{h'-h-k}; C_{u'v'})$) of vertex u_{L-k} means that there is a child x of u_{L-k} such that $T_x \underset{r}{\approx} T_{u_{L-h+h'+k+1}}$, which is a contradiction (since T_x is now a proper subtree of $T_{u_{L-h+h'+k+1}}$). For indices k ($> \frac{h'-h}{2}$), we can apply the same argument vertices from $u'_{h'-h-1}$ to u'_0 to get a contradiction. This proves that C ($= C'$) is symmetric.

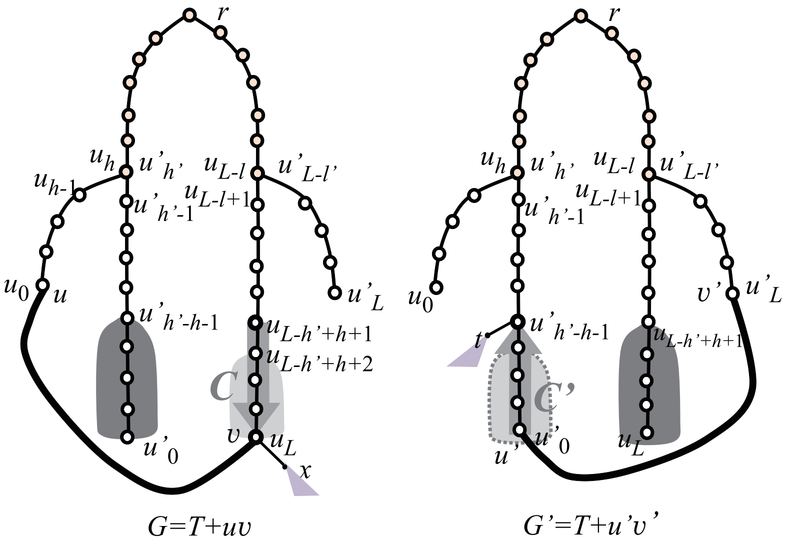

Figure S11: Sequences C and C'.

By applying a similar argument in Case 1(i), we see that T has two subtrees T_{a^*} and T_{b^*} satisfying condition (2), and hence $T + uv$ and $T + u'v'$ admit a rooted-isomorphism, which contradicts the assumption on (u, v) and (u', v').

This completes a proof of this theorem. □

S1.3 Proof of Lemma 3

Proof: For a vertex u in the unique cycle C in a 1-augmented tree G, let G_u denote the component containing u that appears after removing the two edges in C incident to u. Hence G_u is a subtree of G, and we regard it as a subtree rooted at u. Let $\sigma^*(u; C)$ denote the signature of G_u; $\sigma^*(u; C) = \sigma^*(v; C)$ if and only if $G_u \underset{r}{\approx} G_v$. When G is obtained from a tree T by adding an edge uv, the unique cycle in G is denoted by C_{uv}. In a rooted tree, a pair of vertices x and y is called *comparable* if one of them is an ancestor of the other. Let dfs be the depth-first search number obtained by traversing T starting from r, where $r = c_T$ or the left endpoint r of $c_T = rr'$.

Intuitively admissible edges are defined so that each admissible pair (u, v) is lexicographically smaller than any pair (u', v') such that $T + uv \underset{r}{\approx} T + u'v'$.

To prove the lemma, it suffices to show that (I) For any nonadmissible pair of nonadjacent vertices $u, v \in V(T)$, there is an admissible pair (u', v') such that $T + u'v' \underset{r}{\approx} T + uv$; and (II) For two admissible pairs (u, v) and (u', v'), if $T + uv \underset{r}{\approx} T + u'v'$ then $u = u'$ and $v = v'$.

(I) Let (u, v) be a nonadmissible pair of nonadjacent vertices with $\mathrm{dfs}(u) < \mathrm{dfs}(v)$. Hence (u, v) does not satisfy one of conditions (1)-(3) in the definition of admissible pairs. Let $G = T + uv$. To show (I), we assume that (u, v) is lexicographically minimum among all nonadmissible pairs (x, y) such that $T + xy \underset{r}{\approx} G$.

(i) (u, v) does not satisfy condition (1), i.e., there is a vertex $w \in V(P(\mathrm{lca}(u, v), r)) - \{r\}$ such that $\mathrm{copy}(w) = 1$ (possibly $u = w$ when $u = \mathrm{lca}(u, v)$): Then the subtree T_w rooted at w is rooted-isomorphic to the subtree T_x rooted at the left sibling $x = \mathrm{left}(w)$ of w, implying that T_x contains a pair (u', v') such that $T + u'v'$ is rooted-isomorphic to $T + uv$. Then (u', v') must be admissible, since otherwise it would contradict the choice of (u, v).

(ii) (u, v) does not satisfy condition (2), i.e., there is a vertex w with $\mathrm{copy}(w) = 1$ such that (a) $w \in V(P(u, \mathrm{gua}(u, v)) - \{\mathrm{lca}(u, v), \mathrm{gua}(v, u)\}$; or (b) $w \in V(P(v, \mathrm{gua}(v, u)) - \{\mathrm{lca}(u, v), \mathrm{gua}(v, u)\}$: First consider case (a), where $u \neq \mathrm{lca}(u, v)$ (since otherwise $u = \mathrm{lca}(u, v)$, $\mathrm{gua}(u, v) = \mathrm{gua}(v, u)$,

and $V(P(u,\text{gua}(u,v)) - \{\text{lca}(u,v), \text{gua}(v,u)\} = \emptyset)$. As in (i), T_w is rooted-isomorphic to T_x rooted at the left sibling $x = \text{left}(w)$ of w, and T_x contains a vertex u' such that $T + u'v \underset{r}{\approx} T + uv$. Then (u', v) must be admissible by the lexicographical minimality in the choice of (u, v). In (b) (possibly $u = \text{lca}(u, v)$), $T_w \underset{r}{\approx} T_x$ for the left sibling $x = \text{left}(w)$ of w, and T_x contains a vertex v' such that $T + uv' \underset{r}{\approx} T + uv$. Then (u, v') must be admissible by the lexicographical minimality of (u, v).

(iii) (u, v) with $\text{copy}(\text{gua}(v, u)) = 1$ does not satisfy condition (3); i.e., (a) $u = \text{lca}(u, v)$, which violates condition (3-i) $\text{gua}(u, v) = \text{left}(\text{gua}(v, u))$; (b) $u \neq \text{lca}(u, v)$ and $\text{gua}(u, v) \neq \text{left}(\text{gua}(v, u))$; or (c) $u \neq \text{lca}(u, v)$, $\text{gua}(u, v) = \text{left}(\text{gua}(v, u))$, and $\text{dfs}(v) < \text{dfs}(\hat{u})$ for the copy $\hat{u}$ of vertex u in $T_{\text{gua}(v,u)}$.

In (a) and (b), vertex $x = \text{left}(\text{gua}(v, u))$ satisfies $T_x \underset{r}{\approx} T_{\text{gua}(v,u)}$ and T_x contains a vertex v' $(\neq v)$ such that $T + uv' \underset{r}{\approx} T + uv$. Then (u, v') must be admissible by the lexicographical minimality of (u, v). In (c), there is a vertex $\hat{v} \in V(T_{\text{gua}(u,v)})$ such that $T + \hat{v}\hat{u} \underset{r}{\approx} T + uv$. Since $\text{dfs}(\hat{v}) = \text{dfs}(v) - |V(T_{\text{gua}(u,v)})|$ and $\text{dfs}(u) = \text{dfs}(\hat{u}) - |V(T_{\text{gua}(u,v)})|$ hold, $\text{dfs}(v) < \text{dfs}(\hat{u})$ implies $\text{dfs}(\hat{v}) < \text{dfs}(u)$. Then $(\hat{v}, \hat{u})$ must be admissible by the lexicographical minimality of (u, v).

(II) Let (u, v) and (u', v') be two admissible pairs such that $T + uv \underset{r}{\approx} T + u'v'$. Assume without loss of generality that (u, v) is lexicographically smaller than (u', v'), i.e., $\text{dfs}(u) \leq \text{dfs}(u')$, and if $\text{dfs}(u) = \text{dfs}(u')$ then $\text{dfs}(v) \leq \text{dfs}(v')$. We show that $u = u'$ and $v = v'$.

We first claim

$$\text{lca}(u, v) = \text{lca}(u', v'). \tag{11}$$

Note that if $\text{lca}(u, v)$ is not the unicentroid $c_T = r$ then among the codes in $\sigma^*(C_{uv})$ the vertex $\text{lca}(u, v) \in V(C_{uv})$ has the unique code $\sigma^*(\text{lca}(u, v); C_{uv})$, since only subtree $G_{\text{lca}(u,v)}$ of 1-augmented tree $G = T + uv$ contains at least $n/2 + 1$ vertices. Similarly, if $\text{lca}(u', v')$ is not the unicentroid $c_T = r$ the vertex $\text{lca}(u', v') \in V(C_{u'v'})$ has the unique code $\sigma^*(\text{lca}(u', v'); C_{u'v'})$. If $\text{lca}(u, v) = r = \text{lca}(u', v')$ then we are done. The remaining case is that $\text{lca}(u, v) \neq r$ or $\text{lca}(u', v') \neq r$. In this case, we assume that $\text{lca}(u, v) \neq \text{lca}(u', v')$ to derive a contradiction. Without loss generality, we assume that $\text{lca}(u, v) \neq r$. Then $\text{lca}(u', v') \neq r$ also holds, since otherwise for any vertex t in $C_{u'v'}$ (such as $t = \text{lca}(u', v')$), $\sigma^*(C_{u'v'})$ cannot contain a code $\sigma^*(t; C_{u'v'})$ that indicates that the subgraph G'_t of $G' = T + u'v'$ rooted at t has at least $n/2$ vertices. This and $T + uv \underset{r}{\approx} T + u'v'$ mean that $\sigma^*(\text{lca}(u, v); C_{uv}) = \sigma^*(\text{lca}(u', v'); C_{u'v'})$, i.e., the subtrees $G_{\text{lca}(u,v)}$ and $G'_{\text{lca}(u',v')}$ are rooted-isomorphic. This implies that $T_{\text{gua}(x,y)} \underset{r}{\approx} T_{\text{gua}(y,x)}$ for two vertices $x = \text{lca}(u, v)$ and $y = \text{lca}(u', v')$ and $\text{copy}(\text{gua}(x, y)) = 1$ or $\text{copy}(\text{gua}(y, x)) = 1$, contradicting that both (u, v) and (u', v') satisfy the condition (1) in the definition of admissible pairs. This proves (11). Now $\text{gua}(u, v)$, $\text{gua}(v, u)$, $\text{gua}(u', v')$ and $\text{gua}(v', u')$ are children of $\text{lca}(u, v) = \text{lca}(u', v')$ in T.

We next claim

$$u \neq \text{lca}(u, v) = \text{lca}(u', v') \neq u' \tag{12}$$

Assume that $u = \text{lca}(u, v)$ or $u' = \text{lca}(u', v')$, say $u = \text{lca}(u, v)$ (the case of $u' = \text{lca}(u', v')$ can be handled symmetrically). In this case, $u' = \text{lca}(u', v')$ also holds by $\text{lca}(u, v) = \text{lca}(u', v')$, since otherwise the degree of $\text{lca}(u, v)$ in $G = T + uv$ would be larger than that of it in $G' = T + u'v'$, contradicting that $\sigma^*(\text{lca}(u, v); C_{uv}) = \sigma^*(\text{lca}(u', v'); C_{u'v'})$ holds by $T + uv \underset{r}{\approx} T + u'v'$. Now $u = \text{lca}(u, v) = \text{lca}(u', v') = u'$. Assume $v \neq v'$ (otherwise we are done with $u = u'$ and $v = v'$), where $\text{dfs}(v) < \text{dfs}(v')$ is assumed without loss of generality. Clearly v is not an ancestor of v'. Then by $\sigma^*(\text{lca}(u, v); C_{uv}) = \sigma^*(\text{lca}(u', v'); C_{u'v'})$, we have $T_{\text{gua}(v,u)} \underset{r}{\approx} T_{\text{gua}(v',u')}$ and hence $\text{copy}(\text{gua}(v', u')) = 1$. However, in this case, (u', v') with $u' = \text{lca}(u', v')$ does not satisfy condition (3-i) in the definition of admissible pairs, a contradiction. This proves (12).

Thirdly we claim

$$\text{gua}(u, v) = \text{gua}(u', v') \text{ and } \text{gua}(v, u) = \text{gua}(v', u'). \tag{13}$$

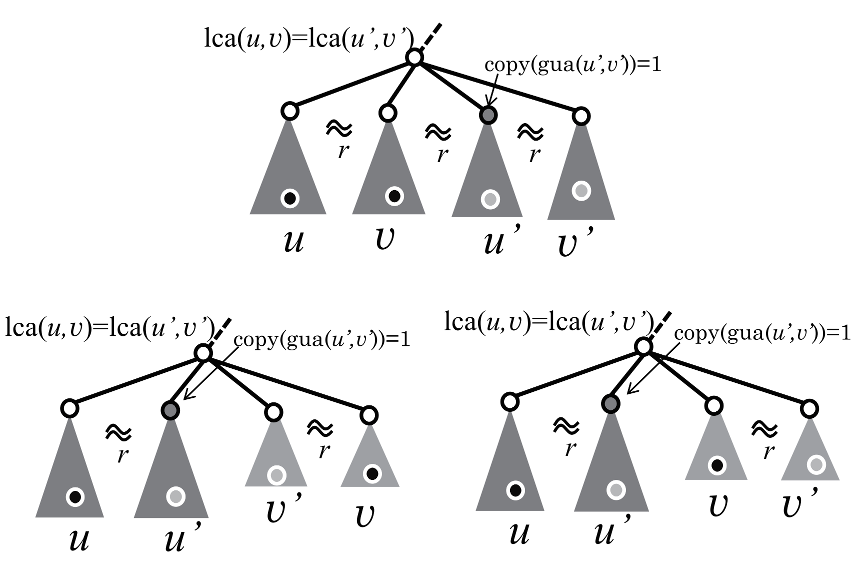

Figure S12: Three cases (c-i), (c-ii) and (c-iii).

Let $a = \mathrm{gua}(u, v)$, $a' = \mathrm{gua}(u', v')$, $b = \mathrm{gua}(v, u)$ and $b' = \mathrm{gua}(v', u')$.

(a) $a \neq a'$ and $b = b'$ (where $\mathrm{dfs}(u) < \mathrm{dfs}(u')$ is assumed without loss of generality): In the isomorphism ψ for $G = T + uv \underset{r}{\approx} G' = T + u'v'$, $\mathrm{lca}(u, v)$ in G corresponds to $\mathrm{lca}(u', v')$ in G', and $\sigma^*(\mathrm{lca}(u, v); C_{uv}) = \sigma^*(\mathrm{lca}(u', v'); C_{u'v'})$ implies that $T_a \underset{r}{\approx} T_{a'}$ and $\mathrm{copy}(a') = \mathrm{copy}(\mathrm{gua}(u', v')) = 1$. Hence (u', v') does not satisfy the condition (2) in the definition of admissible pairs, a contradiction.

(b) $a = a'$ and $b \neq b'$ (where $\mathrm{dfs}(v) < \mathrm{dfs}(v')$ is assumed without loss of generality): As in (a), we see that $T_b \underset{r}{\approx} T_{b'}$ and $\mathrm{copy}(b') = \mathrm{copy}(\mathrm{gua}(v', u')) = 1$. Hence (u', v') does not satisfy the condition (3-i) in the definition of admissible pairs, a contradiction.

(c) $a \neq a'$ and $b \neq b'$ (where $\mathrm{dfs}(u) < \mathrm{dfs}(u')$ is assumed without loss of generality): There are three cases: (c-i) $\mathrm{dfs}(u) < \mathrm{dfs}(v) < \mathrm{dfs}(u') < \mathrm{dfs}(v')$; (c-ii) $\mathrm{dfs}(u) < \mathrm{dfs}(u') < \mathrm{dfs}(v') < \mathrm{dfs}(v)$; and (c-iii) $\mathrm{dfs}(u) < \mathrm{dfs}(u') < \mathrm{dfs}(v) < \mathrm{dfs}(v')$. (See Figure S12.) In (c-i), the subtrees $T_1 = T_a$, $T_2 = T_b$, $T_3 = T_{a'}$ and $T_4 = T_{b'}$ appear in this order from left to right in T, where $T_i \underset{r}{\approx} T_j$ $(i < j)$ implies $T_i \underset{r}{\approx} T_{i'} \underset{r}{\approx} T_j$ for any $i < i' < j$ since T is a left-heavy tree. Hence the isomorphism ψ for $G = T + uv \underset{r}{\approx} G' = T + u'v'$ implies $T_a \underset{r}{\approx} T_{a'}$ and $T_b \underset{r}{\approx} T_{b'}$. Hence we have $T_a \underset{r}{\approx} T_b \underset{r}{\approx} T_{a'} \underset{r}{\approx} T_{b'}$ in (c-i). With a similar argument from (c-i), we have $T_a \underset{r}{\approx} T_{a'}$ and $T_{b'} \underset{r}{\approx} T_b$ in (c-ii); and $T_a \underset{r}{\approx} T_{a'}$ and $T_b \underset{r}{\approx} T_{b'}$ in (c-iii). In any of these three cases, $\mathrm{copy}(\mathrm{gua}(u', v')) = 1$ and (u', v') does not satisfy the condition (2) in the definition of admissible pairs, a contradiction. This proves that $a = a'$ and $b = b'$; i.e., (13).

In a rooted-isomorphism ψ between $G = T + uv$ and $G' = T + u'v'$, vertex $\mathrm{lca}(u, v) = \mathrm{lca}(u', v')$ in G corresponds to itself in G', meaning that there are only two possible rooted-isomorphisms ψ: (i) one preserves the direction of cycles, i.e., $\psi(\mathrm{gua}(u, v)) = \mathrm{gua}(u, v)$; and (ii) the other reverse the direction, i.e., $\psi(\mathrm{gua}(u, v)) = \mathrm{gua}(v, u)$.

We first consider the rooted-isomorphism ψ in (i). When u is an ancestor of u', the degree $\deg(u; G)$ of u in $G = T + uv$ is larger than the degree $\deg(u; G')$ of u in $G' = T + u'v'$, and it cannot hold that $\sigma^*(u; C_{uv}) = \sigma^*(u; C_{u'v'})$, a contradiction to ψ. When neither of u and u' is an ancestor of the other (where $\mathrm{dfs}(u) < \mathrm{dfs}(u')$ is assumed without loss of generality), we have $\sigma^*(\mathrm{lca}(u, u'); C_{uv}) = \sigma^*(\mathrm{lca}(u, u'); C_{u'v'})$, implying that $T_{\mathrm{gua}(u,u')} \underset{r}{\approx} T_{\mathrm{gua}(u',u)}$ and $\mathrm{copy}(\mathrm{gua}(u', u)) = 1$. (See Figure S13.) This contradicts that (u', v') satisfies the condition (2) in the definition of admissible pairs. Finally consider the case where neither of v and v' is an ancestor

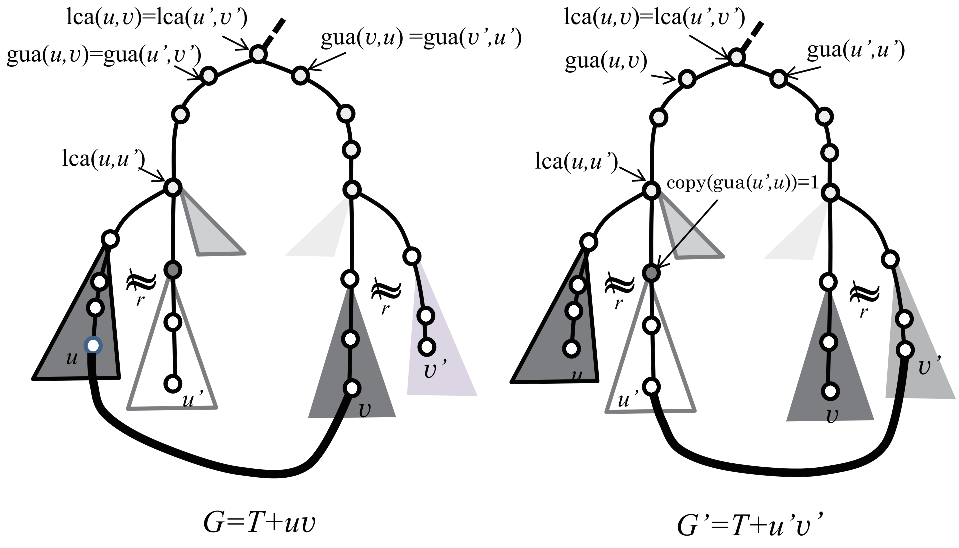

Figure S13: Neither of u and u' is an ancestor of the other.

of the other (where $\mathrm{dfs}(v) < \mathrm{dfs}(v')$ is assumed without loss of generality). As in the above case, we have $T_{\mathrm{gua}(v,v')} \underset{r}{\approx} T_{\mathrm{gua}(v',v)}$ and $\mathrm{copy}(\mathrm{gua}(v',v)) = 1$. This contradicts that (u',v') satisfies the condition (2) in the definition of admissible pairs.

In what follows, we consider the rooted-isomorphism ψ in (ii). Since we know that $G = T + uv$ and $G' = T + u'v'$ are rooted-isomorphic, we have $\psi(\mathrm{gua}(u,v)) = \mathrm{gua}(v,u)$ and $\psi(\mathrm{gua}(v,u)) = \mathrm{gua}(u,v)$. We distinguish the three cases: Case 1. Each of (u,u') and (v,v') is comparable in T; Case 2. Exactly one of (u,u') and (v,v') is comparable in T; and Case 3. Each of (u,u') and (v,v') is incomparable in T.

Case 1. Each of (u,u') and (v,v') is comparable in T: By $\mathrm{dfs}(u) \leq \mathrm{dfs}(u')$, u is an ancestor of u' and v' is an ancestor of v: In this case, the unique path $P(u',v)$ of T connecting u' and v contains vertices u', u, v' and v in this order. Denote the sequence of vertices in $P(u',v)$ by $u_0(= u'), u_1, \ldots, u_h\ (= u), \ldots, u_g\ (= \mathrm{lca}(u,v)), \ldots, u_{L-h}\ (= v'), \ldots, u_L\ (= v)$, where 1-augmented tree $T + uv$ (resp., $T = u'v'$) has a unique cycle $C_{uv} = (u_h, u_{h+1}, \ldots, u_L)$ (resp., $C_{u'v'} = (u_h, u_{h+1}, \ldots, u_{L-h}, u_0, u_1, \ldots, u_{h-1})$) and it holds $|V(C_{uv})| = |V(C_{u'v'})| = L - h + 1$.

(a) $g - h = L - h - g$ (the depth of u in T is equal to that of v' in T): Since $\sigma^*(u_i; C_{uv}) = \sigma^*(u_{L-i}; C_{u'v'})$ for all $i = h, h+1, \ldots, g$, we see that $T_{\mathrm{gua}(u,u')} \underset{r}{\approx} T_{\mathrm{gua}(v,v')}$ and $\mathrm{copy}(\mathrm{gua}(v',u')) = 1$. (See Figure S14.) This contradicts that (u',v') satisfies the condition (3-ii) in the definition of admissible pairs.

(b) $g - h \neq L - h - g$: Consider the case of $g - h < L - h - g$, i.e., the depth of u in T is smaller than that of v' in T (the other case can be treated analogously). (See Figure S15.) In this case, u_h (resp., u_{2g-h}) in $G = T + uv$ is mapped to u_{2g-h} (resp., u_h) in $G' = T + u'v'$ by ψ, and we have $\sigma^*(u_h; C_{uv}) = \sigma^*(u_{2g-h}; C_{u'v'})$ and $\sigma^*(u_{2g-h}; C_{uv}) = \sigma^*(u_h; C_{u'v'})$. By noting that $u_{2g-h} \in V(C_{uv})$, $\sigma^*(u_{2g-h}; C_{uv}) = \sigma^*(u_{2g-h}; C_{u'v'})$, indicating that $\sigma^*(u_h; C_{uv}) = \sigma^*(u_h; C_{u'v'})$. This, however, is impossible because the degree of u_h in G is larger than that in G'.

Case 2. Exactly one of (u,u') and (v,v') is comparable in T: Assume that neither of u and u' is an ancestor of the other and v is an ancestor of v' (the other case can be treated symmetrically). Denote the sequence of vertices in path $P(u,v')$ in T by $u_0\ (= u), u_1, \ldots, u_h\ (= \mathrm{lca}(u,u')), \ldots, u_g\ (= \mathrm{lca}(u,v)), \ldots, u_{L-\ell}\ (= v), \ldots, u_L\ (= v')$, and that in $P(u', \mathrm{lca}(u,u'))$ by $u'_0\ (= u'), u'_1, \ldots, u'_{h'}\ (= u_h)$.

(a) $v \neq v'$ (where $\mathrm{dfs}(v) < \mathrm{dfs}(v')$ is assumed without loss of generality) and $g - h > L - \ell - g$ (the depth of u_h in T is larger than that of v in T): (See Figure S16.) In this case, $u_{L-\ell}$ (resp., $u_{2g-L+\ell}$) in $G = T + uv$ is mapped to $u_{2g-L+\ell}$ (resp., $u_{L-\ell}$) in $G' = T + u'v'$ by ψ, and we

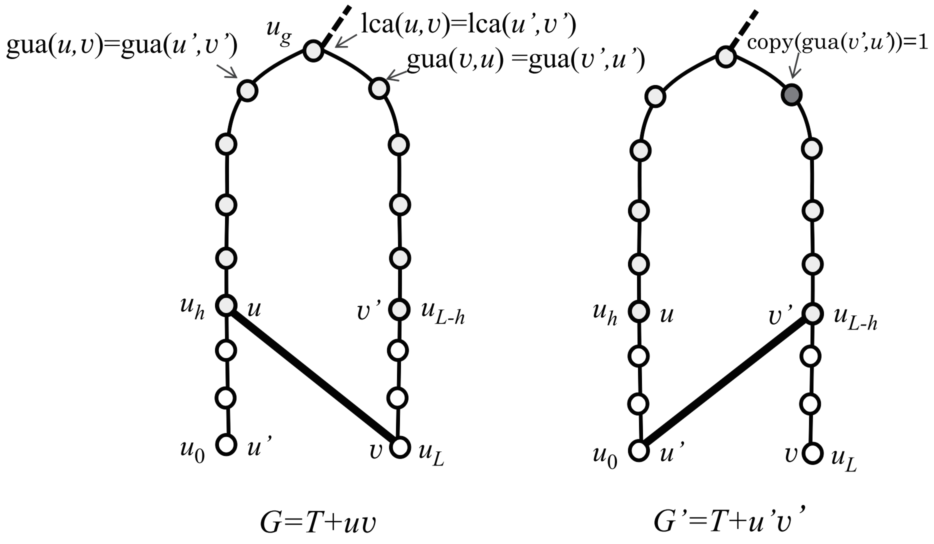

Figure S14: (a) in Case 1.

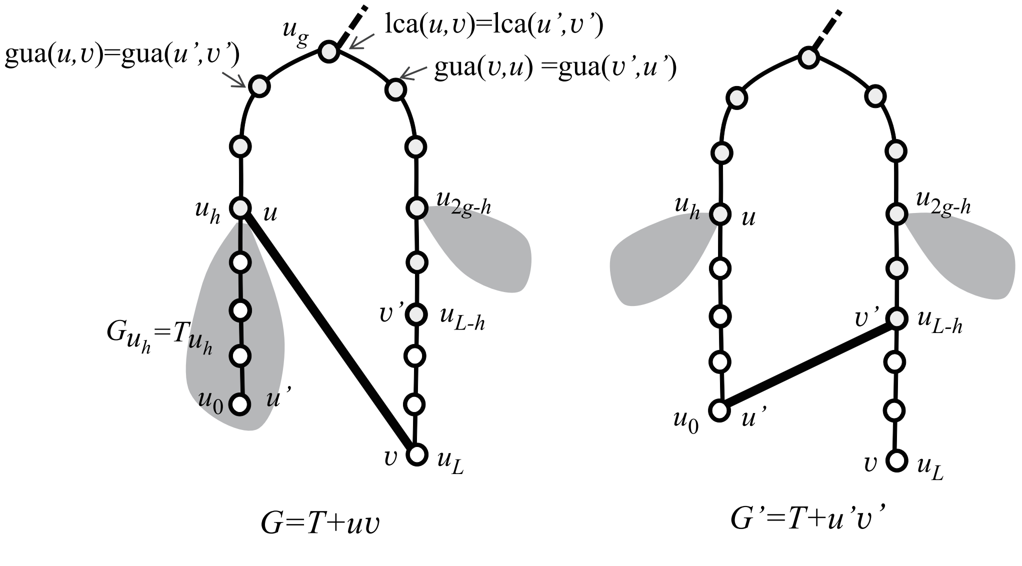

Figure S15: (b) in Case 1.

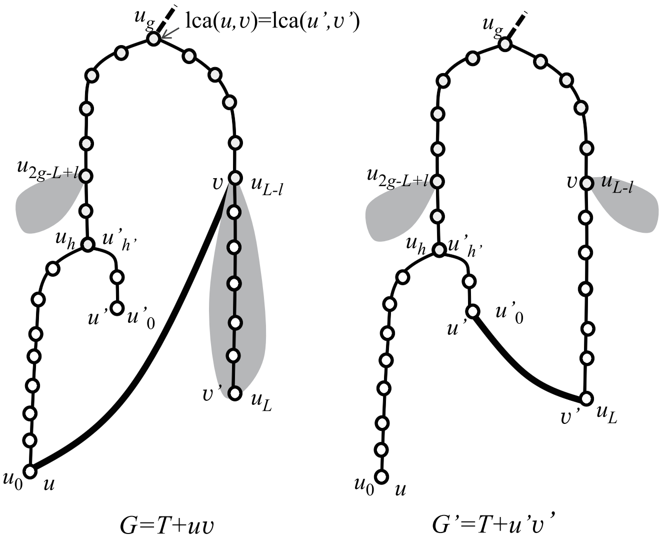

Figure S16: (a) in Case 2.

have $\sigma^*(u_{L-\ell}; C_{uv}) = \sigma^*(u_{2g-L+\ell}; C_{u'v'})$ and $\sigma^*(u_{2g-L+\ell}; C_{uv}) = \sigma^*(u_{L-\ell}; C_{u'v'})$. By noting that $u_{2g-L+\ell} \in V(C_{uv})$, $\sigma^*(u_{2g-L+\ell}; C_{uv}) = \sigma^*(u_{2g-L+\ell}; C_{u'v'})$, indicating that $\sigma^*(u_{L-\ell}; C_{uv}) = \sigma^*(u_{L-\ell}; C_{u'v'})$. This, however, is impossible because the degree of $u_{L-\ell}$ in G is larger than that in G'.

(b) $v \neq v'$ (where $\text{dfs}(v) < \text{dfs}(v')$ is assumed without loss of generality) and $g - h \leq L - \ell - g$ (the depth of u_h in T is not smaller than that of v in T): (See Figure S17.) In this case, u_h (resp., u_{2g-h}) in $G = T + uv$ is mapped to u_{2g-h} (resp., u_h) in $G' = T + u'v'$ by ψ, and we have $\sigma^*(u_h; C_{uv}) = \sigma^*(u_{2g-h}; C_{u'v'})$ and $\sigma^*(u_{2g-h}; C_{uv}) = \sigma^*(u_h; C_{u'v'})$. By noting that $u_{2g-h} \in V(C_{uv})$, it holds $\sigma^*(u_{2g-h}; C_{uv}) = \sigma^*(u_{2g-h}; C_{u'v'})$, indicating that $\sigma^*(u_h; C_{uv}) = \sigma^*(u_h; C_{u'v'})$. This, however, means that $T_{\text{gua}(u,u')} \underset{r}{\approx} T_{\text{gua}(u',u)}$ and $\text{copy}(\text{gua}(u',u)) = 1$, and that (u', v') does not satisfy the condition (2) in the definition of admissible pairs.

(c) $v = v'$: Let us trace how vertex u_h in G will be mapped to vertices in $G' = T + u'v'$ by ψ. Since the code $\sigma^*(u_h; C_{uv})$ represents the set $\mathcal{T}$ of subtrees T_t rooted at children t ($\neq u_{h-1}$) of u_h, vertex u_h cannot be mapped to any vertex in $\{u'_{h'-1}, u'_{h'-2}, \ldots, u'_0\}$ (since these vertices are contained in $T_{u'_{h'-1}} \in \mathcal{T}$). Hence $\psi(u_h) \in \{u_h, u_{h+1}, \ldots, u_{L-\ell}\ (= v = v')\}$. Since $\sigma^*(u_i; C_{uv}) = \sigma^*(u_i; C_{u'v'})$ for these vertices $u_i \in \{u_{h+1}, \ldots, u_{L-\ell}\ (= v = v')\}$, there is an integer $j \geq 1$ such that $\psi^j(u_h) = u_h$. This, however, means that $T_{\text{gua}(u,u')} \underset{r}{\approx} T_{\text{gua}(u',u)}$ and $\text{copy}(\text{gua}(u',u)) = 1$, and that (u', v') does not satisfy the condition (2) in the definition of admissible pairs.

Case 3. Each of (u, u') and (v, v') is incomparable in T: Assume that $u \neq u'$ and $\text{dfs}(u) < \text{dfs}(u')$ without loss of generality. Denote the sequence of vertices in the path $P(u, v)$ by $u_0\ (= u), u_1, \ldots, u_h\ (= \text{lca}(u, u')), \ldots, u_g\ (= \text{lca}(u, v)), \ldots, u_{L-\ell}\ (= \text{lca}(v, v')), \ldots, u_L\ (= v)$, and those in $P(u', v')$ by $u'_0\ (= u'), u'_1, \ldots, u'_{h'}\ (= \text{lca}(u, u')), \ldots, u'_{g'}\ (= u_g = \text{lca}(u, v)), \ldots, u'_{L-\ell'}\ (= \text{lca}(v, v')), \ldots, u'_L\ (= v')$, where $h + \ell = h' + \ell'$.

(a) $g - h \neq L - \ell - g$: Consider the case of $g - h < L - \ell - g$, i.e., the depth of $\text{lca}(u, u')$ in T is larger than that of $\text{lca}(v, v')$ in T (the other case can be treated analogously). As in Case 2(b), u_h (resp., u_{2g-h}) in $G = T + uv$ is mapped to u_{2g-h} (resp., u_h) in $G' = T + u'v'$ by ψ, and we have $\sigma^*(u_h; C_{uv}) = \sigma^*(u_{2g-h}; C_{u'v'})$ and $\sigma^*(u_{2g-h}; C_{uv}) = \sigma^*(u_h; C_{u'v'})$. By noting that $u_{2g-h} \in V(C_{uv})$, $\sigma^*(u_{2g-h}; C_{uv}) = \sigma^*(u_{2g-h}; C_{u'v'})$, indicating that $\sigma^*(u_h; C_{uv}) = \sigma^*(u_h; C_{u'v'})$. This meas that $T_{\text{gua}(u,u')} \underset{r}{\approx} T_{\text{gua}(u',u)}$ and $\text{copy}(\text{gua}(u',u)) = 1$, and that (u', v') does not satisfy the condition (2) in the definition of admissible pairs.

(b) $g - h = L - \ell - g$ (the depth of $\text{lca}(u, u')$ in T is equal to that of $\text{lca}(v, v')$ in T): Let

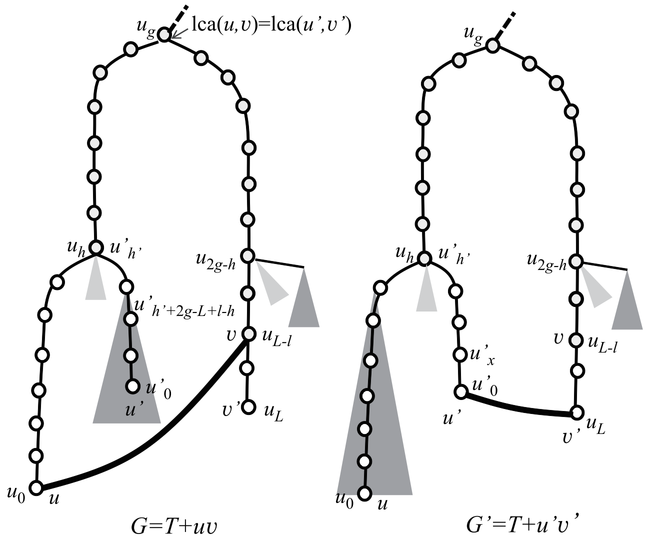

Figure S17: (b) in Case 2.

$\mathcal{T}_{u_h}$ (resp., $\mathcal{T}_{u_{L-\ell}}$) denote the set of subtrees T_t of T rooted at a child t of u_h (resp., $u_{L-\ell}$). Let $a = \text{gua}(u, u')$, $a' = \text{gua}(u', u)$, $b = \text{gua}(v, v')$ and $b' = \text{gua}(v', v)$, where $T_a \not\approx_r T_{a'}$ and $T_b \not\approx_r T_{b'}$ (otherwise (u, v) or (u', v') would not satisfy the condition (2) in the definition of admissible pairs). (See Figure S18.) Let A be the subsequence of $\sigma^*(C_{uv})$ from $b = u_{L-\ell+1}$ to $a = u_{h-1}$, i.e., $A = (\sigma^*(u_{L-\ell+1}; C_{uv}), \sigma^*(u_{L-\ell+2}; C_{uv}), \ldots, u_L, u_0, \ldots, \sigma^*(u_{h-1}; C_{uv}))$, and let A' be the subsequence of the reversal of $\sigma^*(C_{u'v'})$ from $a' = u'_{h'-1}$ to $b' = u'_{L-\ell'+1}$, i.e., $A' = (\sigma^*(u'_{h'-1}; C_{u'v'}), \sigma^*(u'_{h'-2}; C_{u'v'}), \ldots, u'_0, u'_L, \ldots, \sigma^*(u'_{L-\ell'+1}; C_{u'v'}))$. Then $A = A'$ by the isomorphism ψ. Since u_h (resp., u_{2g-h}) in $G = T+uv$ is mapped to $u_{L-\ell}$ (resp., u_h) in $G' = T+u'v'$ by ψ, we have $\sigma^*(u_h; C_{uv}) = \sigma^*(u_{L-\ell}; C_{u'v'})$ and $\sigma^*(u_{L-\ell}; C_{uv}) = \sigma^*(u_h; C_{u'v'})$. This means that the set $\mathcal{T}_{u_h} - \{T_a\}$ of subtrees is rooted-isomorphic to $\mathcal{T}_{u_{L-\ell}} - \{T_{b'}\}$, while $\mathcal{T}_{u_h} - \{T_{a'}\}$ of subtrees is rooted-isomorphic to $\mathcal{T}_{u_{L-\ell}} - \{T_b\}$. Since $T_a \not\approx_r T_{a'}$ and $T_b \not\approx_r T_{b'}$, this implies that $T_a \approx_r T_{b'}$, $T_b \approx_r T_{a'}$, and $T_{\text{gua}(u,v)} \approx_r T_{\text{gua}(v,u)}$.

Hence by assumption of $\text{dfs}(u) < \text{dfs}(u')$, we have $\text{dfs}(u) < \text{dfs}(u') < \text{dfs}(v') < \text{dfs}(v)$. Let $\hat{v} \in V(T_{a'})$ be the symmetric copy of $v \in V(T_b)$ and $\hat{u} \in V(T_{b'})$ be the symmetric copy of $u \in V(T_a)$. Let $\hat{A}$ be the subsequence of the reversal of $\sigma^*(C_{\hat{v}\hat{u}})$ from $a' = u'_{h'-1}$ to $b = u_{L-\ell+1}$. Then $T + uv \approx_r T + \hat{v}\hat{u}$ and $\hat{A} = A = A'$. Now we show that $u' = \hat{v}$ and $v' = \hat{u}$, which indicates that (u', v') does not satisfy the condition (3-ii) in the definition of admissible pairs.

Assume $u' \neq \hat{v}$ (the case of $v' \neq \hat{u}$ can be treated analogously). (See Figure S19.) Note that $u', \hat{v} \in V(T_{a'})$. When one of u' and $\hat{v}$ is an ancestor of the other in $T_{a'}$ (say $\hat{v}$ is an ancestor of u'), $\hat{v} \in V(C_{\hat{v}\hat{u}}) \cap V(C_{u'v'})$ holds but $\sigma^*(\hat{v}; C_{\hat{v}\hat{u}}) \neq \sigma^*(\hat{v}; C_{u'v'})$ (since the degree of $\hat{v}$ in $T + \hat{v}\hat{u}$ is larger than that in $T + u'v'$), contradicting $\hat{A} = A'$. Hence the remaining case is that neither of u' and $\hat{v}$ is an ancestor of the other. Let $t_1 = \text{gua}(u', \hat{v})$ and $t_2 = \text{gua}(\hat{v}, u')$, which are children of $\text{lca}(u', \hat{v})$. Then by $T + u'v' \approx_r T + \hat{v}\hat{u}$, we have $\sigma^*(\text{gua}(u', \hat{v}); C_{u'v'}) = \sigma^*(\text{gua}(\hat{v}, u'); C_{\hat{v}\hat{u}})$ by $\hat{A} = A'$, which implies that $T_{t_1} \approx_r T_{t_2}$. If $\text{dfs}(\hat{v}) < \text{dfs}(u')$ then $\text{copy}(\text{gua}(u', \hat{v})) = 1$ holds, indicating that (u', v') does not satisfy the condition (2) in the definition of admissible pairs. On the other hand ($\text{dfs}(u') < \text{dfs}(\hat{v})$) $\text{copy}(\text{gua}(\hat{v}, u')) = 1$ holds. Then the symmetric copy of $w \in V(T_b)$ of $\text{gua}(\hat{v}, u')$ also satisfies $\text{copy}(w) = 1$, indicating that (u', v') does not satisfy the condition (2) in the definition of admissible pairs. □

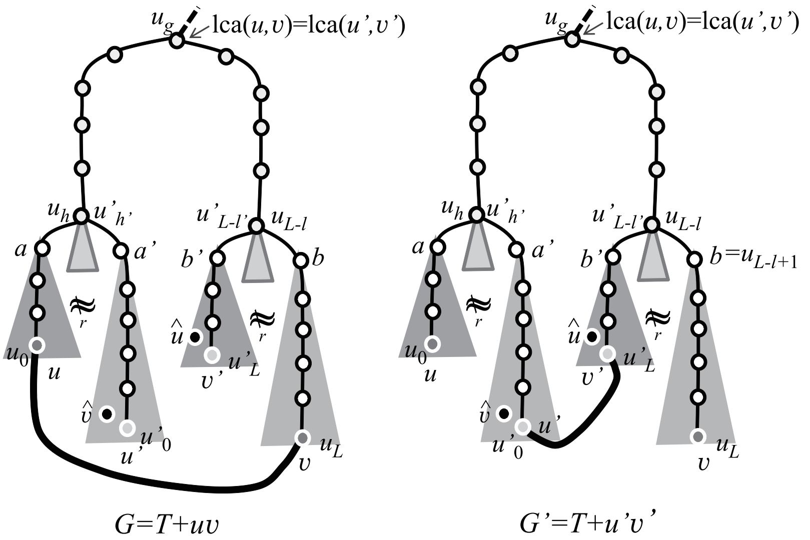

Figure S18: (b) in Case 3.

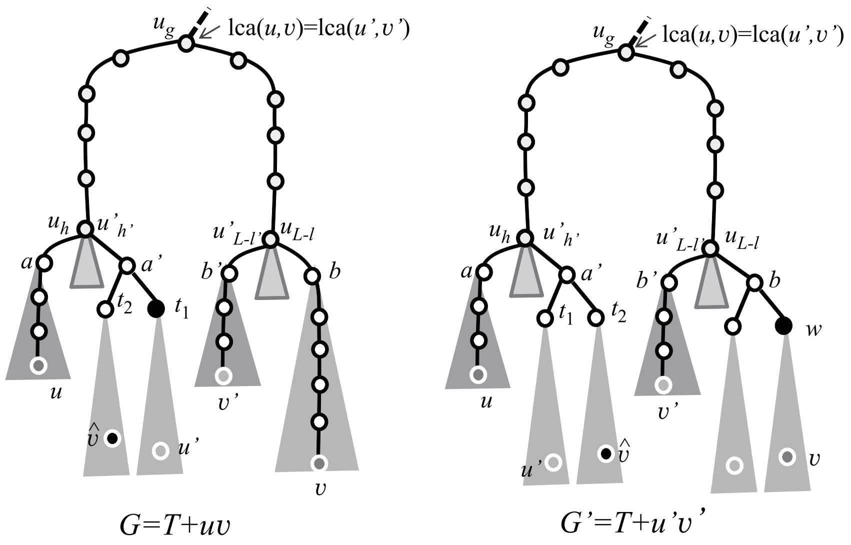

Figure S19: (b) in Case 3 (assuming that $u' \neq \hat{u}$)

S1.4 Proof of Lemma 4

Proof: For a given rooted left-heavy multi-tree T, we prove that all candidate pairs of T can be found in $O(n^2)$ time. Recall that a vertex pair (u, v) is a candidate pair if it is an admissible pair and "no heavy edge is created in $T + uv$ or u is an ancestor of v." For the given T, we denote the vertex set by $V = \{v_0, v_1, \dots, v_{n-1}\}$, indexed by depth-first-search started at the root r from left to right. To find all candidate pairs in $O(n^2)$ time, we examine all vertex pairs in the dfs order; i.e., we select all vertex pairs (u, v) $(\mathrm{dfs}(u) < \mathrm{dfs}(v))$ in T by choosing each vertex u $(= v_i)$ from v_0 to v_{n-1} and choosing another vertex v $(= v_j)$ from v_{i+1} to v_{n-1}. For each vertex pair (u, v), we first check whether it is a pair such that "no heavy edge is created in $T + uv$ or u is an ancestor of v." We will see that a test of admissible conditions for such a pair (u, v) can be done easily without preparing data gua().

Note that for a given vertex pair (u, v), it holds $\mathrm{lca}(u, v) = u$ if and only if u is an ancestor of v. Also $\mathrm{lca}(u, v) \neq u$ holds if and only if there is a vertex w such that $\mathrm{dfs}(u) < \mathrm{dfs}(w) \leq \mathrm{dfs}(v)$ and $\mathrm{depth}(u) \leq \mathrm{depth}(w)$. As will be shown later, we can compute whether $\mathrm{lca}(u, v) = u$ or not for all vertex pairs (u, v) in T in $O(n^2)$ time in total.

To facilitate testing whether $T + uv$ has a heavy edge or not, we introduce some more notions. For each vertex v in T, we define

$$\mathrm{numCopy}[v] = \text{“the number of vertices } u \text{ in path } P(r, v) \text{ such that } \mathrm{copy}(u) = 1.\text{”}$$

We define R to be the set of children of the root when T has the unicentroid and to be the set of the bicentroids otherwise (see Figure S20). Let $d_R(T)$ denote the depth of the vertices in R in T; i.e.,

$$d_R(T) = \begin{cases} 1 & T \text{ has the unicentroid} \\ 0 & T \text{ has the bicentroid.} \end{cases}$$

Then we index the vertices in R as $r_0, r_1, \dots, r_{k-1}$ $(k = |W|)$ so that $\mathrm{dfs}(r_i) < \mathrm{dfs}(r_j)$ $0 \leq i < j < k$. For each vertex v in T, we define $r[v]$ to be the vertex $w \in W$ that is an ancestor of v or v itself (where we define $r[v_0] = v_0$ $(= r)$ if T has the unicentroid), and let $i_R[v]$ to be the integer h such that $r_h = r[v]$, i.e., v is in T_{r_h}, where $r[v] = r_{i_R[v]}$. Observe that for a given vertex pair (u, v), it holds $r[u] \neq r[v]$ if and only if $T + uv$ has no heavy edge.

The following procedure Preprocess computes $\mathrm{numCopy}[v]$, $r[v]$ and $i_R[v]$ for all vertices $v \in V(T)$ in $O(n)$ time in total.

Algorithm Preprocess
Input: A rooted left-heavy multi-tree T consisting of vertices $V = \{v_0, v_1, \dots, v_{n-1}\}$ (indexed by depth-first-search started at r from left to right) and depth $\mathrm{depth}(v)$ and $\mathrm{copy}(v) \in \{0, 1\}$ for all vertices $v \in V(T)$.
Output: $\mathrm{numCopy}[v]$, $r[v]$ and $i_R[v]$ for all vertices $v \in V(T)$.

Set $i_R[v_0] := 0$ if T has the bicentroid and $i_R[v_0] := -1$ otherwise;
$r[v_0] := v_0$;
for $i := 1, \dots, n-1$ **do**
  Let $p(v_i)$ be the parent of v_i in T (where we let $p(v_{n/2}) = v_0$ if T has the bicentroid);
  $\mathrm{numCopy}[v_i] := \mathrm{numCopy}[p(v_i)] + \mathrm{copy}(v_i)$
  if depth $\mathrm{depth}(v_i)$ of v_i is equal to $d_R(T)$ **then**
    $i_R[v_i] := i_R[v_{i-1}] + 1$;
    $r[v_i] := v_i$
  else
    $i_R[v_i] := i_R[v_{i-1}]$;
    $r[v_i] := r[v_{i-1}]$
  end if
end for

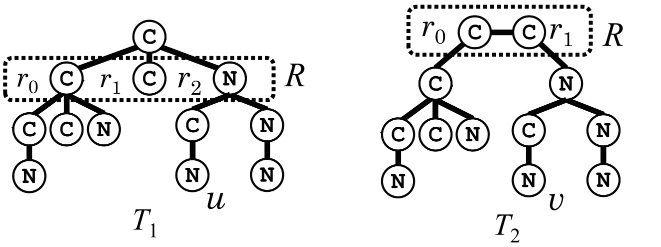

(a) T_1 has the unicentroid (b) T_2 has the bicentroid

Figure S20: Illustration for sets R of vertices, enclosed by dotted lines, where $i_R[u] = 2$ and $r[u] = r_2$ in T_1, and $i_R[v] = 1$ and $r[v] = r_1$ in T_2.

Suppose that we know whether $\mathrm{lca}(u, v) = u$ or not for all vertex pairs (u, v) in T. Let (u, v) be a vertex pair that appears during our examination of all vertex pairs in T in a dfs order. By the dfs order, it holds $\mathrm{dfs}(u) < \mathrm{dfs}(v)$. It is easy to check whether u and v are not adjacent each other in T (i.e., (u, v) is an edge in T) in $O(1)$ time. Now we can test whether $T + uv$ has no heavy edge or not in $O(1)$ time by checking whether $r[u] \neq r[v]$ holds or not. Also we can test whether u is an ancestor of v or not in $O(1)$ time by checking whether $\mathrm{lca}(u, v) = u$ holds or not. In what follows, we consider the case where a vertex pair (u, v) to be examined satisfies $\mathrm{dfs}(u) < \mathrm{dfs}(v)$ and "$T + uv$ has no heavy edge or u is an ancestor of v." We distinguish two cases.

(I) $T + uv$ has no heavy edge (i.e., $r[u] < r[v]$): The admissible conditions (1) and (2) can be restated as $\mathrm{copy}(w) = 0$ for all vertices w in $V(P(u, r)) \cup V(P(v, r)) - \{r\}$ except $w = \mathrm{gua}(v, u)$. This is equivalent with "$\mathrm{numCopy}[u] = \mathrm{numCopy}[v] = 0$" or "$\mathrm{numCopy}[u] = 0$, $\mathrm{numCopy}[v] = 1$ and $\mathrm{copy}(\mathrm{gua}(v, u)) = 1$." Hence we only need to know the value of $\mathrm{copy}(\mathrm{gua}(v, u))$ in order to test whether (u, v) satisfies admissible conditions (1) and (2). In this case, by $r[u] < r[v]$, we have $\mathrm{gua}(u, v) = r[u]$ and $\mathrm{gua}(v, u) = r[v]$. Hence we obtain $\mathrm{copy}(\mathrm{gua}(v, u))$ by $\mathrm{copy}(r[v])$. Suppose that (u, v) satisfies admissible conditions (1) and (2), and consider admissible condition (3). We easily see that admissible condition (3)-(i) holds if and only if $i_R[u] - i_R[v] = 1$; and admissible condition (3)-(ii) holds if and only if $\mathrm{dfs}(v) \geq \mathrm{dfs}(u) + |V(T_{r[u]})|$, where $|V(T_{r[u]})|$ can be determined in $O(1)$ time using the dfs number on vertices. Thus, we can test whether (u, v) satisfies admissible condition (3) or not in $O(1)$ time.

(II) u is an ancestor of v (i.e., $\mathrm{lca}(u, v) = u$): Recall that (3)-(i) implies that $\mathrm{copy}(\mathrm{gua}(v, u))$ in (2) needs to be 0 when $\mathrm{lca}(u, v) = u$, and that we do not need to check admissible condition (3). Now admissible conditions (1) and (2) can be restated as $\mathrm{numCopy}[u] = \mathrm{numCopy}[v] = 0$, and hence we can test whether (u, v) satisfies admissible conditions (1) and (2) in $O(1)$ time.

From the above argument, we can test each of the $O(n^2)$ vertex pairs in T in $O(1)$ time to leave only candidate pairs, and the total time complexity is $O(n^2)$. We describe the entire $O(n^2)$-time algorithm that finds all candidate pairs for a multi-tree T in the following, where whether $\mathrm{lca}(u, v) = u$ or not is tested for all vertex pairs (u, v) in T.

Algorithm CandidatePair
Input: A rooted left-heavy multi-tree T consisting of vertices $V = \{v_0, v_1, \ldots, v_{n-1}\}$ (indexed by depth-first-search started at r from left to right), and data $\mathrm{numCopy}[v]$, $i_R[v]$ and $r[v]$ for all vertices $v \in V(T)$.
Output: All candidate nonadjacent pairs.

/* $lca = 1$ if $\mathrm{lca}(v_i, v_j) = v_i$; $lca = 0$ if $\mathrm{lca}(v_i, v_j) \neq v_i$ */
for $i := 0, \ldots, n-1$ **do**
  $lca := 1$;
  if $\mathrm{numCopy}[v_i] = 0$ **then**
    for $j := i+1, \ldots, n-1$ **do**
      $lca := 1$;

if depth(v_i) $\leq$ depth(v_j) **then**
  $lca := 0$
end if;
/*
  (i) "$lca = 1$ or $i_R[v_i] \neq i_R[v_j]$"; and v_i and v_j are not adjacent in T.
  (ii) numCopy$[v_j] = 0$.
  (iii) copy$(r[v_j])$ = numCopy$[v_j] = i_R[v_j] - i_R[v_i] = 1$; and dfs$(v_j) \geq$ dfs$(v_i) + |T_{r[v_i]}|$.
*/
if (v_i, v_j) satisfies (i) and one of (ii) and (iii) **then**
  Output (v_i, v_j) as a candidate pair
end if
end for
end if
end for

□

Now we can generate all children of T as follows.

for each candidate nonadjacent pair $x, y \in V(T)$ generated by Algorithm CandidatePair **do**
  Check whether T is the parent $\pi(T + xy)$ of $T + xy$ (i.e., $T + xy$ is a child of T)
  by using Algorithm ChildTest.

By Lemma 1 and Lemma 4, the above procedure takes $O(n^4)$ time, as stated in Lemma 5. Note that some of the generated children may not be feasible, i.e., violating the required condition $g_L \leq f_K(G) \leq g_U$. A feasible child $G = T + xy$ is a 1-augmented multi-graph in $\mathcal{G}_1(g_L, g_U)$.

S2 2-phase algorithm for generating multi-trees

In this section, we describe the 2-Phase Algorithm [4] for generating all multi-trees in $\mathcal{G}_0(g'_L, g_U)$. The algorithm consists of two phases: The first phase enumerates a set $\mathcal{S}$ of simple trees that contains the underlying simple graphs of all multi-trees in $\mathcal{G}_0(g'_L, g_U)$, and the second phase assigns multiplicities to all edges in each of the simple trees in $\mathcal{S}$ so that all the multi-trees in $\mathcal{G}_0(g'_L, g_U)$ will be obtained. The idea of the 2-phase algorithm for generating multi-trees T that satisfies a single feature vector g (i.e., $T \in \mathcal{G}_0(g, g)$) is due to Ishida et al. [2], and the extension of a problem with a single feature vector to that with a set of feature vectors between lower and upper vectors is first introduced by Shimizu et al. [3].

First Phase To obtain such a set $\mathcal{S}$ of simple trees, we modify given lower and upper vectors g'_L and g_U for a multi-tree generation problem into lower and upper vectors g'_l and g_u for a problem of generating simple trees in $\mathcal{S} = \mathcal{G}_0(g'_l, g_u)$ as follows. Let $g'_L, g_U \in \mathcal{F}_K(\Sigma, d)$ be given lower and upper vectors on the feature vectors of multi-trees T to be generated, where $g'_L[t] = g_U[t]$ for all $t \in \Sigma^{0,d}$ and $n = \sum_{t\in\Sigma^{0,d}} g'_L[t]$ is the number of vertices in T. Then we prepare lower and upper feature vectors $g'_l, g_u \in \mathcal{F}_K(\Sigma, 1)$ so that the underlying simple graph of any multi-tree in $\mathcal{G}_0(g'_L, g_U)$ is contained in $\mathcal{G}_0(g'_l, g_u)$. Formally, g'_l and g_u will be constructed as follows. Given an alternating sequence $s = (c_0, m_1, c_1, \ldots, m_k, c_k) \in \Sigma^{\leq K,d}$, let $\widetilde{s}$ denote the sequence obtained by replacing all multiplicities with 1s; i.e., $\widetilde{s} = (c_0, 1, c_1, \ldots, 1, c_k)$. Then for each sequence $t \in \Sigma^{\leq K,1}$, let

$$g'_l[t] = \textstyle\sum_{s\in\Sigma^{\leq K,d}:\widetilde{s}=t} g'_L[s] \text{ and } g_u[t] = \sum_{s\in\Sigma^{\leq K,d}:\widetilde{s}=t} g_U[s].$$

The first phase generates simple trees by appending a new vertex starting with a tree of a single vertex. Not to output the same simple tree more than once, we use a left-heavy ordered tree τ of a simple tree T as the canonical form of T. For an unrooted tree T and a vertex $v_0 \in V(T)$ (resp., an edge $vv' \in E(T)$), let (T, v_0) (resp., (T, vv')) denote the tree T rooted at v_0 (resp., vv'), where both v and v' will be the highest vertices in (T, vv'). Any tree T has either a centroid or a bicentroid. We always choose the centroid v_0 (or bicentroid vv') of a tree T as the root, since this establishes a one-to-one correspondence between unrooted trees and rooted trees. For notational convenience, we only describe the case of enumerating trees with centroids. Let λ be an order that determines an ordering among each set of siblings (the set $\mathrm{Ch}(v)$ of children of each non-leaf vertex v) in a rooted tree (T, v_0). Thus there are $\Pi_{v\in V(T)}|\mathrm{Ch}(v)|!$ way of choosing such a sibling order λ. The ordered tree τ of a rooted tree (T, v_0) with a sibling order λ is denoted by (T, v_0, λ). Let $\Lambda(T, v_0)$ denote the set of ordered trees $\tau = (T, v_0, \lambda)$ over all sibling orders λ. An ordered tree $\tau \in \Lambda(T, v_0)$ is called a *left-heavy tree* of a rooted tree (T, v_0) when the color-depth sequence $\delta(\tau)$ is lexicographically maximum among all ordered trees in $\Lambda(T, v_0)$.

Given lower and upper vectors $g'_l, g_u, \in \mathcal{F}_K(\Sigma, 1)$ such that $g'_l[t] = g_u[t]$ for all $t \in \Sigma^{0,d}$, we know that the number of vertices of a simple tree to be generated is $n = \sum_{t\in\Sigma^{0,d}} g'_l[t]$.

We start with a rooted tree τ_c with a single vertex v_0 with a color $c(v_0) = c \in \Sigma$, and try to enumerate all left-heavy trees τ^* rooted at v_0 with n vertices such that $\tau^* \in \Lambda(T, v_0)$ for some simple tree $T \in \mathcal{G}_0(g'_l, g_u)$. We construct all such trees τ^* by appending new leafs to τ_c. Suppose that we have a left-heavy tree τ rooted at v_0 with less than n vertices. For a vertex $r \in V(\tau)$ and a color $c \in \Sigma$, let $\tau + (r, c)$ denote the ordered tree τ' obtained from τ by appending a new vertex p with $c(p) = c$ to r; i.e., $V(\tau') = V(\tau) \cup \{p\}$, $E(\tau') = E(\tau) \cup \{rp\}$ and $c(p) = c$. We design a recursive procedure $\mathrm{Gen}(\tau)$ that generates all ordered trees $\tau + (r, c)$ which remain left-heavy, where we may discard some ordered trees $\tau + (r, c)$ if they have no chance to be extended to any of the target left-heavy trees τ^*. More specifically, we discard any left-heavy tree $\tau' = \tau + (r, c)$ if it violates one of the following conditions.

(i) For each child u of the root v_0 in τ', the number of descendants of u (including u) is at most $(n - 1)/2$ (otherwise the root v_0 of τ cannot be the centroid of any tree with n vertices obtained by appending vertices to τ');

(ii) $\deg(v; \tau') \leq \mathrm{val}(c(v))$ for all $v \in V(\tau')$;

(iii) $f_K(\tau') \leq g_u$; and

(iv) $g'_l \leq f_K(\tau')$ when $|V(\tau')| = n$.

We call a left-heavy tree τ' *valid* if it satisfies all the above conditions. Procedure Gen(τ) recursively calls Gen(τ') for each valid left-heavy tree $\tau' = \tau + (r, c)$. It is known [5] that each pair (r, c) such that $\tau + (r, c)$ remains left-heavy can be found in $O(1)$ time. The entire description of the first phase is given as follows.

Algorithm FirstPhase
Input: A color set Σ, a maximum path length $K \in \mathbb{Z}_+$, lower and upper vectors $g'_l, g_u, \in \mathcal{F}_K(\Sigma, 1)$ such that $g'_l[t] = g_u[t]$ for all $t \in \Sigma^{0,d}$. Let $n = \sum_{t\in\Sigma^{0,d}} g'_l[t]$.
Output: All simple trees $T \in \mathcal{G}_0(g'_l, g_u)$, which are required to be generated without duplication as left-heavy ordered trees. Each simple tree $T \in \mathcal{G}_0(g'_l, g_u)$ is output as the left-heavy ordered tree $\tau \in \Lambda(T, v_0)$ rooted at the centroid v_0 of T.

for each color $c \in \Sigma$ **do**
  Let τ_c be the left-heavy tree consisting of a single vertex v with $c(v) = c$;
  if τ_c is valid **then**
    Gen(τ_c)
  end if
end for

Procedure Gen(τ)
if τ has n vertices **then**
  Output τ
else
  for each pair of a vertex $r \in V(\tau)$ and a color $c \in \Sigma$ such that $\tau + (r, c)$ is a left-heavy tree **do**
    Append a new leaf p with $c(p) = c$ to r to obtain a left-heavy tree $\tau' := \tau + (r, c)$;
    if τ' is valid **then**
      Gen(τ')
    end if
  end for
end if

Second Phase Now we explain the second phase. Let T be a simple tree which is represented by a left-heavy tree rooted at its centroid v_0, and let $p(v)$ denote the parent of a non-root vertex v in T. We let the vertices in T be indexed as $v_0, v_1, v_2, \ldots, v_{n-1}$ in the depth first search order, where $n = |V(T)|$ and v_0 is the root of T, and let e_i, $i = 1, 2, \ldots, n-1$ denote the edge $v_i p(v_i)$. Let $\overline{m}$ be the maximum multiplicity given by the input feature vector. A multi-tree obtained from T by assigning a multiplicity $m_i \in [1, \overline{m}]$ to each edge e_i, $i = 1, 2, \ldots, n-1$ is denoted by a pair (T, M) of T and a *multiplicity sequence* $M = (m_1, m_2, \ldots, m_{n-1})$.

Note that for some two distinct multiplicity sequences M and M', two multi-trees (T, M) and (T, M') may be isomorphic as unordered trees rooted at v_0; i.e., T has another left-heavy representation T' such that M' will correspond to M in the transformation from T to T'. We call a multi-tree (T, M) a *canonical form* if M is the lexicographically largest among all multiplicity sequences M' such that (T, M) and (T, M') are isomorphic as unordered trees rooted at v_0.

We can test whether a given multi-tree (T, M) is a canonical form or not without comparing with any other multi-trees (T, M').

For this, we define some more notions on subtrees of multi-trees. For a vertex u in a multi-tree (T, M), let $T(u)$ denote the simple left-heavy subtree of T rooted at vertex u, and $M(u)$ denote the subsequence of M induced by the edges in $T(u)$. Similarly, for an edge e joining a non-root vertex u and its parent $p(u)$, let $T(e)$ denote the simple left-heavy tree rooted at $p(u)$ which consists of e and $T(u)$, and $M(e)$ denote the subsequence of M induced by the edges in $T(e)$. For a non-root vertex $u \in V(T)$, let left(u) denote the immediate left sibling of u (if any), and e_u denote the edge between

vertex u and its parent $p(u)$. Notice that $DL(T(\text{left}(u))) = DL(T(v))$ holds if and only if $T(u)$ is isomorphic to $T(\text{left}(u))$ as unordered trees rooted at u. When $DL(T(\text{left}(u))) = DL(T(u))$, any two vertices $v_j \in V(\text{left}(u))$ and $v_i \in V(T(u))$ corresponding each other in the isomorphism satisfy $j = i - |T(u)|$. For such vertices u, v_i and v_j, we denote v_j by $\psi(v_i, u)$.

Then we observe that a multi-tree (T, M) with a left-heavy tree T rooted at v_0 is a canonical form if and only if every vertex $u \in V(T)$ with a left sibling left(u) such that $DL(T(\text{left}(u))) = DL(T(u))$ (i.e., $T(\text{left}(u))$ and $T(u)$ are isomorphic left-heavy trees) satisfies

$$M(\text{left}(u)) \text{ is not lexicographically smaller than } M(u). \tag{14}$$

Thus we can test whether a given multi-tree (T, M) is a canonical form or not only by checking a condition at each vertex $u \in V(T)$.

Now our task is to generate all canonical forms (T, M) for a given left-heavy tree T. We design a procedure that assignss multiplicities $m_i \geq 1$ to edges e_i in the depth first search order not to violate the required condition at each vertex $u \in V(T)$ so that a full set $M = (m_1, m_2, \ldots, m_{n-1})$ of multiplicities of all edges will give rise to a canonical form (T, M). After the k-th edge e_k receives a multiplicity $m_k \geq 1$ during an execution of the procedure, the first k edges e_i, $1 \leq i \leq k$ have received multiplicity $m_i \geq 1$ and the remaining $n-1-k$ edges e_i, $k < i \leq n-1$ have not yet received multiplicity. For notational convenience, we let $m_i = 0$ mean that the edge e_i has not yet received any multiplicity during an execution of the procedure. The multiplicity sequence of a tree T immediately after a multiplicity is assigned to the k-th edge is denoted by $M = (m_1, m_2, \ldots, m_k, 0, \ldots, 0)$.

We are supposed to assign a multiplicity m_k of the k-th edge e_k, where the current multiplicity sequence is given by $M = (m_1, m_2, \ldots, m_{k-1}, 0, \ldots, 0)$ ($1 \leq m_i \leq \overline{m}$ for $i = 1, 2, \ldots, k-1$). There may be a value $x \in [1, \overline{m}]$ such that assigning multiplicity $m_k = x$ results in a multiplicity sequence $(m_1, m_2, \ldots, m_{k-1}, m_k = x, \ldots, 0)$ that cannot lead to a canonical form (T, M') no matter how multiplicities of the remaining edges $e_{k+1}, e_{k+2}, \ldots, e_{n-1}$ are chosen to construct a full set $M' = (m_1, m_2, \ldots, m_{k-1}, m_k = x, m_{k+1}, m_{k+1}, \ldots, m_{n-1})$ of multiplicities in T. Note that for any larger value $x' > x$, assigning multiplicity $m_k = x'$ cannot lead to a canonical form either.

For two subsequences M_1 and M_2 of consecutive $(a+1)$ elements in M, where $M_1 = (m_i, m_{i+1}, \ldots, m_{i+a})$ ($m_j \geq 0$ for $i \leq j \leq i+a$) and $M_2 = (m_{i'}, m_{i'+1} \ldots, m_{i'+a})$ ($m'_j \geq 0$ for $i' \leq j' \leq i'+a$), we denote $M_1 \supseteq M_2$ if there is an integer $b \in [0, a]$ such that $m_{i+j} = m_{i'+j}$ for $0 \leq j < b$ and $m_{i+j'} = 0$ for $b \leq j' \leq a$.

When we assign a multiplicity m_k to the k-th edge e_k, where the current multiplicity sequence is $M = (m_1, \ldots, m_{k-1}, 0, \ldots, 0)$, $m_i \in [1, \overline{m}]$ for $1 \leq i \leq k-1$, we identify all vertices $u \in V(T)$ such that choosing a large value for m_k may violate condition (14). We define mcopy$[u; k] = 1$ for such a vertex $u \in V(T)$ and mcopy$[u; k] = 0$ otherwise; i.e.,

$$\text{mcopy}[u; k] = \begin{cases} 1 & DL(T(\text{left}(u))) = DL(T(u)) \text{ and} \\ & M(e_{\text{left}(u)}) \supseteq M(e_u) \\ 0 & \text{otherwise,} \end{cases}$$

where $M(e_{\text{left}(u)}) \supseteq M(e_u)$ means that currently $M(e_{\text{left}(u)})$ is not lexicographically larger than $M(e_u)$, but it can be larger than $M(e_u)$ if $T(u)$ contains the k-th edge and a large value is chosen for m_k. Note that $M(e_u)$ contains the entry m_k if and only if vertex u is on the path from edge e_k and the root v_0. Hence to find the largest value that can be chosen for m_k, we check the vertices u with mcopy$[u; k] = 1$ along the path from edge $e_k = p(v_k)v_k$ and the root v_0. The next lemma tells that we only need to use the highest vertex among such vertices.

Lemma S1. [2] *For a multiplicity sequence $M = (m_1, \ldots, m_{k-1}, 0, \ldots, 0)$, $m_i \in [1, \overline{m}]$ for $1 \leq i \leq k-1$ in a left-heavy tree T rooted at v_0, assume that vertex v_k of the k-th edge $e_k = p(v_k)v_k$ has two ancestors u and u' such that* mcopy$[u; k] =$ mcopy$[u'; k] = 1$. *For $v_j = \psi(v_k, u)$ and $v_h = \psi(v_k, u')$, let m_j and m_k be the multiplicities of edges $e_j = v_j p(v_j)$ and $e_h = v_h p(v_h)$, respectively, where*

$j < h$ (*i.e., u is an ancestor of u'*). *Then when we fix a value for m_k, the following holds:*

$$m_j \geq m_k \Longrightarrow m_h \geq m_k;$$
$$m_j > m_k \Longrightarrow m_h > m_k.$$

The lemma says that if a fixed value for m_k does not violate condition (14) for vertex u then it will not violate condition (14) for vertex u' either. We let a variable cd to store the depth of the highest ancestor u of v_k with $\mathrm{mcopy}[u;k] = 1$, where cd is set to be an integer such that $cd \geq \mathrm{depth}(v_i)$ if no such ancestor exits.

Procedure AssignMultiplicity(T, M, k, cd) assigns a multiplicity $m_k \in [1, \overline{m}]$ to the k-th edge $e_k = v_k p(v_k)$, where $M = (m_1, \ldots, m_{k-1}, 0, \ldots, 0)$, i.e., each edge e_i with $i = 1, 2, \ldots, k-1$ has received a multiplicity $m_i \in [1, \overline{m}]$. Let $M' = (m_1, \ldots, m_k, 0, \ldots, 0)$ be a multiplicity sequence obtained immediately after assigning a multiplicity $m_k \in [1, \overline{m}]$. Then (T, M') is called *valid* if it satisfies the following conditions:

(i) $\deg(v; (T, M')) \leq \mathrm{val}(c(v))$ for all $v \in V(T)$;

(ii) In the multi-tree $T^{(k)} = (T, M') - \{e_{k+1}, e_{k+2}, \ldots, e_{n-1}\}$ induced from (T, M') by the edges $e_1, e_2, \ldots, e_k$ with determined multiplicities, the number of paths corresponding to each sequence $t \in \Sigma^{\leq K,d}$ does not exceed its upper bound $g_U[t]$ (i.e., $f_K(T^{(k)}) \leq g_U$); and

(iii) $g'_L \leq f_K((T, M')) \leq g_U$ when $m_{n-1} \geq 1$.

For each valid (T, M'), AssignMultiplicity(T, M, k, cd) recursively calls AssignMultiplicity$(T, M', k+1, cd)$ after updating variable cd. The entire description of the second phase is given as follows.

Algorithm SECONDPHASE
Input: A color set Σ, a maximum path length $K \in \mathbb{Z}_+$, a simple left-heavy tree $T \in \mathcal{G}_0(g'_l, g_u)$ rooted at the centroid, and lower and upper vectors $g'_L, g_U, \in \mathcal{F}_K(\Sigma, d)$ such that $g'_l[t] = g'_L[t] = g_u = g_U[t]$ for all $t \in \Sigma^{0,d}$. Let $n = \sum_{t \in \Sigma^{0,d}} g'_L[t]$, and let $\overline{m}$ be the maximum multiplicity of an edge in the path corresponding to a vector $t \in \Sigma^{\leq K,d}$ such that $g'_U[t] \geq 1$.
Output: All multi-trees in $\mathcal{G}_0(g'_L, g_U)$ obtained by assigning a multiplicity to T, which are required to be generated without duplication.

Let $M = (m_1, m_2, \ldots, m_{n-1})$ with $m_1 := m_2 := \cdots := m_{n-1} := 0$;
Compute $\mathrm{mcopy}[v_i; 1]$, $i = 1, 2, \ldots, n-1$ according to the definition of mcopy;
AssignMultiplicity$(T, M, 1, 1)$;

Procedure AssignMultiplicity(T, M, k, cd);
if $cd < \mathrm{depth}(v_k)$ **then**
  r_{cd} := (the ancestor of vertex v_i whose depth is cd);
  $\mathrm{Mul}_{\max} := m_j$ for $v_j = \psi(v_k, r_{cd})$
else if $\mathrm{mcopy}[v_k; k] = 1$ **then**
    $\mathrm{Mul}_{\max} := m_j$ for $v_j = \psi(v_k, v_k)$; $cd := \mathrm{depth}(v_k)$
 else
    $\mathrm{Mul}_{\max} := \overline{m}$; $cd := \mathrm{depth}(v_k) + 1$
end if;
for $x := 1$ to $\mathrm{Mul}_{\max}$ **do**
  $m_k := x$; $M' := (m_1, \ldots, m_k, 0, \ldots, 0)$;
  if $cd \leq \mathrm{depth}(v_k)$ **then** $cd := \mathrm{depth}(v_k) + 1$ **end if**;
  if (T, M') is valid **then**
    if $k = n$ **then**
      Output (T, M')
    else
      AssignMultiplicity$(T, M', k+1, cd)$
    end if
  end if
end for

S3 An entire algorithm for EULF

We are now ready to describe our algorithm for enumerating all multigraphs $G \in \mathcal{G}_1(g_L, g_U)$ as a collection of procedures we have explained in the paper.

Algorithm Enumerate
Input: A maximum path length $K \in \mathbb{Z}_+$ and feature vectors $g_L, g_U \in \mathcal{F}_K(\Sigma, d)$ such that $g_L[t] = g_U[t]$ for all $t \in \Sigma^{0,d}$.

Output: All 1-augmented multigraphs $G \in \mathcal{G}_1(g_L, g_U)$.

Construct a modified lower vector g'_L from g_L as follows:
  For each $t \in \Sigma^{k,d}$ with $k \geq 2$, let $g'_L[t] := 0$; and
  for each $t \in \Sigma^{1,d}$, let
    $g'_L[t] := \max\{g_L[t] - 1, 0\}$ if t is symmetric (i.e., it is identical with its reversal);
    $g'_L[t] := \max\{g_L[t] - 2, 0\}$ otherwise;
  /* It holds that $\mathcal{G}'_0 \subseteq \mathcal{G}_0(g'_L, g_U)$ for the set $\mathcal{G}'_0$ of 0-augmented $T = G - e$ obtained from each 1-augmented tree $G \in \mathcal{G}_1(g_L, g_U)$ by removing a simple edge e in the unique cycle of G. */

  /* First Phase */
Construct lower and upper feature vectors $g'_l, g_u \in \mathcal{F}_K(\Sigma, 1)$ as follows:
  For each sequence $t \in \Sigma^{\leq K,1}$, let
  $g'_l[t] := \sum_{s \in \Sigma^{\leq K,d}: \widetilde{s} = t} g'_L[s]$ and $g_u[t] := \sum_{s \in \Sigma^{\leq K,d}: \widetilde{s} = t} g_U[s]$;
  /* The underlying simple graph of any multi-tree in $\mathcal{G}_0(g'_L, g_U)$ is contained in $\mathcal{G}_0(g'_l, g_u)$ */

for each simple tree $\tau \in \mathcal{G}_0(g'_l, g_u)$ generated by Algorithm FirstPhase **do**

    /* Second Phase */
  for each multi-tree $T \in \mathcal{G}_0(g'_L, g_U)$ obtained by assigning a multiplicity M to τ by Algorithm SecondPhase **do**

      /* Creating a cycle with a new edge xy */
    Compute numCopy$[v]$, $r[v]$ and $i_R[v]$ for all vertices $v \in V(T)$ by Algorithm Preprocess;
    for each candidate pair $x, y \in V(T)$ listed by Algorithm CandidatePair **do**
      Check whether T is the parent $\pi(T + xy)$ of $T + xy$ (i.e., $T + xy$ is a child of T) or not by Algorithm ChildTest;
      if $G = T + xy$ is a child of T and is feasible (i.e., $g_L \leq f_K(G) \leq g_U$) **then**
        Output G as a 1-augmented multi-graph in $\mathcal{G}_1(g_L, g_U)$
    end for
  end for
end for

S4 Some figures for chemical graphs and upper and lower feature vectors used in the computational experiment

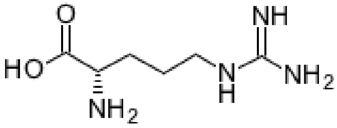

C00062

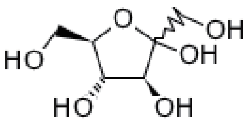

C00095

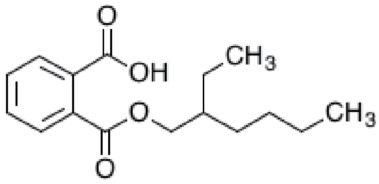

C03343

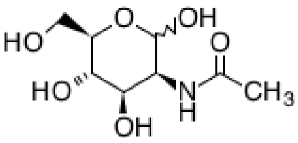

C00645

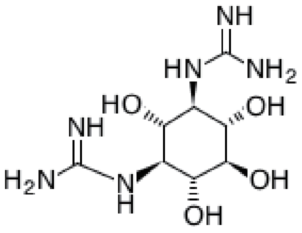

C00837

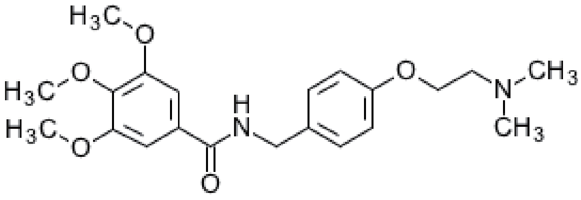

C07178

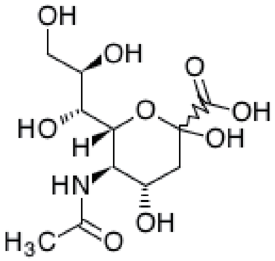

C00270

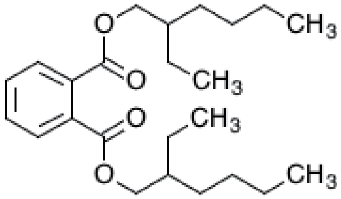

C03690

Figure S21: Illustration for chemical graphs used in computational experiment (I).

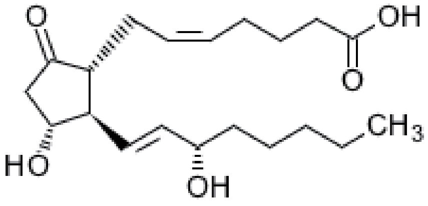

D00079

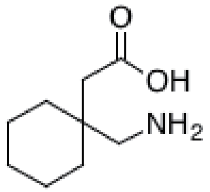

D00332

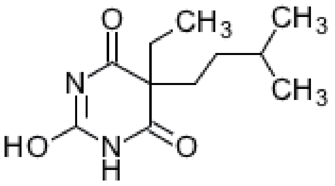

D00555

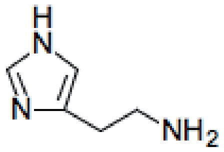

D08040

Figure S22: Illustration for chemical graphs used in computational experiment (II).

component	g_L	g_U
O	2	2
N	4	4
C	6	6
O-C	0	2
O=C	0	2
C-N	3	5
C=N	0	2
C-C	6	10

Figure S23: The lower and upper feature vectors g_L and g_U with $K = 1$ and $w = 1$ created from C00062, where $g_L[t] = g_U[t] = 0$ for any sequence $t \in \Sigma^{\leq K,d}$ not in the table.

component	g_L	g_U
O	2	2
N	4	4
C	6	6
O-C	0	2
O=C	0	2
C-N	3	5
C=N	0	2
C-C	6	10
O-C=O	0	2
C-C-O	0	2
N-C-N	0	4
N-C=N	1	3
C-N-C	0	4
O-C=C	0	2
O=C-C	0	2
N-C-C	2	4
C-C-C	4	8

Figure S24: The lower and upper feature vectors g_L and g_U with $K = 2$ and $w = 1$ created from C00062, where $g_L[t] = g_U[t] = 0$ for any sequence $t \in \Sigma^{\leq K,d}$ not in the table.

References

[1] Fujiwara, H., Wang, J., Zhao, L., Nagamochi, H., Akutsu, T., Enumerating Tree-like Chemical Graphs with Given Path Frequency, *J. Chem. Inf. Model.*, 48, 1345–1357, 2008. 47, 1734–1746, 2007.

[2] Ishida Y, Zhao L, Nagamochi H, Akutsu T: **Improved algorithms for enumerating tree-like chemical graphs with given path frequency.** *Genome Informatics* 2008, **21**:53-64.

[3] Shimizu M, Nagamochi H, Akutsu T: **Enumerating tree-like chemical graphs with given upper and lower bounds on path frequencies.** *BMC Bioinformatics* 2011, **12**(Suppl 14):53.

[4] Suzuki M, Nagamochi H, Akutsu T: **A 2-phase algorithm for enumerating tree-like chemical graphs satisfying given upper and lower bounds.** *IPSJ SIG Technical Reports* 2012, 2012-BIO-28(17):1-8.

[5] Nakano S, Uno T: **Generating colored trees.** *Lecture Notes in Computer Science* 2005, **3787**:249-260.
